# Publisher Correction: Stroke genetics informs drug discovery and risk prediction across ancestries

**DOI:** 10.1038/s41586-022-05492-5

**Published:** 2022-11-14

**Authors:** Aniket Mishra, Rainer Malik, Tsuyoshi Hachiya, Tuuli Jürgenson, Shinichi Namba, Daniel C. Posner, Frederick K. Kamanu, Masaru Koido, Quentin Le Grand, Mingyang Shi, Yunye He, Marios K. Georgakis, Ilana Caro, Kristi Krebs, Yi-Ching Liaw, Felix C. Vaura, Kuang Lin, Bendik Slagsvold Winsvold, Vinodh Srinivasasainagendra, Livia Parodi, Hee-Joon Bae, Ganesh Chauhan, Michael R. Chong, Liisa Tomppo, Rufus Akinyemi, Gennady V. Roshchupkin, Naomi Habib, Yon Ho Jee, Jesper Qvist Thomassen, Vida Abedi, Jara Cárcel-Márquez, Marianne Nygaard, Hampton L. Leonard, Chaojie Yang, Ekaterina Yonova-Doing, Maria J. Knol, Adam J. Lewis, Renae L. Judy, Tetsuro Ago, Philippe Amouyel, Nicole D. Armstrong, Mark K. Bakker, Traci M. Bartz, David A. Bennett, Joshua C. Bis, Constance Bordes, Sigrid Børte, Anael Cain, Paul M. Ridker, Kelly Cho, Zhengming Chen, Carlos Cruchaga, John W. Cole, Phil L. de Jager, Rafael de Cid, Matthias Endres, Leslie E. Ferreira, Mirjam I. Geerlings, Natalie C. Gasca, Vilmundur Gudnason, Jun Hata, Jing He, Alicia K. Heath, Yuk-Lam Ho, Aki S. Havulinna, Jemma C. Hopewell, Hyacinth I. Hyacinth, Michael Inouye, Mina A. Jacob, Christina E. Jeon, Christina Jern, Masahiro Kamouchi, Keith L. Keene, Takanari Kitazono, Steven J. Kittner, Takahiro Konuma, Amit Kumar, Paul Lacaze, Lenore J. Launer, Keon-Joo Lee, Kaido Lepik, Jiang Li, Liming Li, Ani Manichaikul, Hugh S. Markus, Nicholas A. Marston, Thomas Meitinger, Braxton D. Mitchell, Felipe A. Montellano, Takayuki Morisaki, Thomas H. Mosley, Mike A. Nalls, Børge G. Nordestgaard, Martin J. O’Donnell, Yukinori Okada, N. Charlotte Onland-Moret, Bruce Ovbiagele, Annette Peters, Bruce M. Psaty, Stephen S. Rich, Jonathan Rosand, Marc S. Sabatine, Ralph L. Sacco, Danish Saleheen, Else Charlotte Sandset, Veikko Salomaa, Muralidharan Sargurupremraj, Makoto Sasaki, Claudia L. Satizabal, Carsten O. Schmidt, Atsushi Shimizu, Nicholas L. Smith, Kelly L. Sloane, Yoichi Sutoh, Yan V. Sun, Kozo Tanno, Steffen Tiedt, Turgut Tatlisumak, Nuria P. Torres-Aguila, Hemant K. Tiwari, David-Alexandre Trégouët, Stella Trompet, Anil Man Tuladhar, Anne Tybjærg-Hansen, Marion van Vugt, Riina Vibo, Shefali S. Verma, Kerri L. Wiggins, Patrik Wennberg, Daniel Woo, Peter W. F. Wilson, Huichun Xu, Qiong Yang, Kyungheon Yoon, The COMPASS Consortium, Joshua C. Bis, Jin-Moo Lee, Yu-Ching Cheng, James F. Meschia, Wei Min Chen, Michèle M. Sale, Alan B. Zonderman, Michele K. Evans, James G. Wilson, Adolfo Correa, Matthew Traylor, Cathryn M. Lewis, Cara L. Carty, Alexander Reiner, Jeffrey Haessler, Carl D. Langefeld, Rebecca F. Gottesman, Kristine Yaffe, Yong Mei Liu, Charles Kooperberg, Leslie A. Lange, Karen L. Furie, Donna K. Arnett, Oscar R. Benavente, Raji P. Grewal, Leema Reddy Peddareddygari, The INVENT Consortium, Charles Kooperberg, Kristian Hveem, Sara Lindstrom, Lu Wang, Erin N. Smith, William Gordon, Astrid van Hylckama Vlieg, Mariza de Andrade, Jennifer A. Brody, Jack W. Pattee, Jeffrey Haessler, Ben M. Brumpton, Pierre Suchon, Ming-Huei Chen, Kelly A. Frazer, Constance Turman, Marine Germain, James MacDonald, Sigrid K. Braekkan, Sebastian M. Armasu, Nathan Pankratz, Rebecca D. Jackson, Jonas B. Nielsen, Franco Giulianini, Marja K. Puurunen, Manal Ibrahim, Susan R. Heckbert, Theo K. Bammler, Bryan M. McCauley, Kent D. Taylor, James S. Pankow, Alexander P. Reiner, Maiken E. Gabrielsen, Jean-François Deleuze, Chris J. O’Donnell, Jihye Kim, Barbara McKnight, Peter Kraft, John-Bjarne Hansen, Frits R. Rosendaal, John A. Heit, Weihong Tang, Pierre-Emmanuel Morange, Andrew D. Johnson, Christopher Kabrhel, The Dutch Parelsnoer Initiative (PSI) Cerebrovascular Disease Study Group, Ewoud J. van Dijk, Peter J. Koudstaal, Gert-Jan Luijckx, Paul J. Nederkoorn, Robert J. van Oostenbrugge, Marieke C. Visser, Marieke J. H. Wermer, L. Jaap Kappelle, The Estonian Biobank, Tõnu Esko, Andres Metspalu, Reedik Mägi, Mari Nelis, The PRECISE4Q Consortium, The FinnGen Consortium, The NINDS Stroke Genetics Network (SiGN), Marguerite R. Irvin, Frank-Erik de Leeuw, Christopher R. Levi, Jane Maguire, Jordi Jiménez-Conde, Pankaj Sharma, Cathie L. M. Sudlow, Kristiina Rannikmäe, Reinhold Schmidt, Agnieszka Slowik, Joanna Pera, Vincent N. S. Thijs, Arne G. Lindgren, Andreea Ilinca, Olle Melander, Gunnar Engström, Kathryn M. Rexrode, Peter M. Rothwell, Tara M. Stanne, Julie A. Johnson, John Danesh, Adam S. Butterworth, Laura Heitsch, Giorgio B. Boncoraglio, Michiaki Kubo, Alessandro Pezzini, Arndt Rolfs, Anne-Katrin Giese, David Weir, Rebecca D. Jackson, Owen A. Ross, Robin Lemmons, Martin Soderholm, Mary Cushman, Katarina Jood, Caitrin W. McDonough, Steven Bell, Birgit Linkohr, Tsong-Hai Lee, Jukka Putaala, The MEGASTROKE Consortium, Christopher D. Anderson, Oscar L. Lopez, Xueqiu Jian, Ulf Schminke, Natalia Cullell, Pilar Delgado, Laura Ibañez, Jerzy Krupinski, Vasileios Lioutas, Koichi Matsuda, Joan Montaner, Elena Muiño, Jaume Roquer, Chloe Sarnowski, Naveed Sattar, Gerli Sibolt, Alexander Teumer, Loes Rutten-Jacobs, Masahiro Kanai, Anne-Katrin Giese, Solveig Gretarsdottir, Natalia S. Rost, Salim Yusuf, Peter Almgren, Hakan Ay, Steve Bevan, Robert D. Brown, Caty Carrera, Julie E. Buring, Wei-Min Chen, Ioana Cotlarciuc, Paul I. W. de Bakker, Anita L. DeStefano, Marcel den Hoed, Qing Duan, Stefan T. Engelter, Guido J. Falcone, Rebecca F. Gottesman, Stefan Gustafsson, Ahamad Hassan, Elizabeth G. Holliday, George Howard, Fang-Chi Hsu, Erik Ingelsson, Tamara B. Harris, Brett M. Kissela, Dawn O. Kleindorfer, Claudia Langenberg, Robin Lemmens, Didier Leys, Wei-Yu Lin, Erik Lorentzen, Patrik K. Magnusson, Patrick F. McArdle, Sara L. Pulit, Kenneth Rice, Saori Sakaue, Bishwa R. Sapkota, Christian Tanislav, Gudmar Thorleifsson, Unnur Thorsteinsdottir, Christophe Tzourio, Cornelia M. van Duijn, Matthew Walters, Nicholas J. Wareham, Najaf Amin, Hugo J. Aparicio, John Attia, Alexa S. Beiser, Claudine Berr, Mariana Bustamante, Valeria Caso, Seung Hoan Choi, Ayesha Chowhan, Jean-François Dartigues, Hossein Delavaran, Marcus Dörr, Ian Ford, Wander S. Gurpreet, Anders Hamsten, Atsushi Hozawa, Martin Ingelsson, Motoki Iwasaki, Sara Kaffashian, Lalit Kalra, Olafur Kjartansson, Manja Kloss, Daniel L. Labovitz, Cathy C. Laurie, Linxin Li, Lars Lind, Cecilia M. Lindgren, Hirata Makoto, Naoko Minegishi, Andrew P. Morris, Martina Müller-Nurasyid, Bo Norrving, Soichi Ogishima, Eugenio A. Parati, Nancy L. Pedersen, Markus Perola, Pekka Jousilahti, Silvana Pileggi, Raquel Rabionet, Iolanda Riba-Llena, Marta Ribasés, Jose R. Romero, Anthony G. Rudd, Antti-Pekka Sarin, Ralhan Sarju, Mamoru Satoh, Norie Sawada, Ásgeir Sigurdsson, Albert Smith, O. Colin Stine, David J. Stott, Konstantin Strauch, Takako Takai, Hideo Tanaka, Emmanuel Touze, Shoichiro Tsugane, Andre G. Uitterlinden, Einar M. Valdimarsson, Sven J. van der Lee, Kenji Wakai, Stephen R. Williams, Charles D. A. Wolfe, Quenna Wong, Taiki Yamaji, Dharambir K. Sanghera, Kari Stefansson, Kent D. Taylor, Nicolas Martinez-Majander, Kenji Sobue, Carolina Soriano-Tárraga, Henry Völzke, The SIREN Consortium, Onoja Akpa, Fred S. Sarfo, Albert Akpalu, Reginald Obiako, Kolawole Wahab, Godwin Osaigbovo, Lukman Owolabi, Morenikeji Komolafe, Carolyn Jenkins, Oyedunni Arulogun, Godwin Ogbole, Abiodun M. Adeoye, Joshua Akinyemi, Atinuke Agunloye, Adekunle G. Fakunle, Ezinne Uvere, Abimbola Olalere, Olayinka J. Adebajo, The China Kadoorie Biobank Collaborative Group, Junshi Chen, Robert Clarke, Rory Collins, Yu Guo, Chen Wang, Jun Lv, Richard Peto, Yiping Chen, Zammy Fairhurst-Hunter, Michael Hill, Alfred Pozarickij, Dan Schmidt, Becky Stevens, Iain Turnbull, Canqing Yu, The VA Million Veteran Program, The International Stroke Genetics Consortium (ISGC), Quentin Le Grand, Leslie E. Ferreira, The Biobank Japan, Akiko Nagai, Yoishinori Murakami, The CHARGE Consortium, Mirjam I. Geerlings, Natalie C. Gasca, Vilmundur Gudnason, Marion van Vugt, Rebecca F. Gottesman, Eric J. Shiroma, Sigurdur Sigurdsson, Mohsen Ghanbari, Eric Boerwinkle, Alexa S. Beiser, Bernard Fongang, Ruiqi Wang, Mohammad K. Ikram, Uwe Völker, The GIGASTROKE Consortium, Phil L. de Jager, Rafael de Cid, Børge G. Nordestgaard, Muralidharan Sargurupremraj, Shefali S. Verma, Iona Y. Millwood, Christian Gieger, Toshiharu Ninomiya, Hans J. Grabe, J. Wouter Jukema, Ina L. Rissanen, Daniel Strbian, Young Jin Kim, Pei-Hsin Chen, Ernst Mayerhofer, Joanna M. M. Howson, Marguerite R. Irvin, Hieab Adams, Sylvia Wassertheil-Smoller, Kaare Christensen, Mohammad A. Ikram, Tatjana Rundek, Bradford B. Worrall, G. Mark Lathrop, Moeen Riaz, Eleanor M. Simonsick, Janika Kõrv, Paulo H. C. França, Ramin Zand, Kameshwar Prasad, Ruth Frikke-Schmidt, Frank-Erik de Leeuw, Thomas Liman, Karl Georg Haeusler, Ynte M. Ruigrok, Peter Ulrich Heuschmann, W. T. Longstreth, Keum Ji Jung, Lisa Bastarache, Guillaume Paré, Scott M. Damrauer, Daniel I. Chasman, Jerome I. Rotter, Christopher D. Anderson, John-Anker Zwart, Teemu J. Niiranen, Myriam Fornage, Yung-Po Liaw, Sudha Seshadri, Israel Fernández-Cadenas, Robin G. Walters, Christian T. Ruff, Mayowa O. Owolabi, Jennifer E. Huffman, Lili Milani, Yoichiro Kamatani, Martin Dichgans, Stephanie Debette

**Affiliations:** 1 Bordeaux Population Health Research Center, University of Bordeaux, Inserm, UMR 1219, Bordeaux, France; 2 Institute for Stroke and Dementia Research (ISD), University Hospital, LMU Munich, Munich, Germany; 3 Iwate Tohoku Medical Megabank Organization, Iwate Medical University, Iwate, Japan; 4 Estonian Genome Centre, Institute of Genomics, University of Tartu, Tartu, Estonia; 5 Institute of Mathematics and Statistics, University of Tartu, Tartu, Estonia; 6 Department of Statistical Genetics, Osaka University Graduate School of Medicine, Suita, Japan; 7 Massachusetts Veterans Epidemiology Research and Information Center (MAVERIC), VA Boston Healthcare System, Boston, MA USA; 8 TIMI Study Group, Boston, MA USA; 9 Division of Cardiovascular Medicine, Brigham and Women’s Hospital, Harvard Medical School, Boston, MA USA; 10 Division of Molecular Pathology, Institute of Medical Sciences, The University of Tokyo, Tokyo, Japan; 11 Laboratory of Complex Trait Genomics, Graduate School of Frontier Sciences, The University of Tokyo, Tokyo, Japan; 12 Center for Genomic Medicine, Massachusetts General Hospital, Boston, MA USA; 13 Program in Medical and Population Genetics, Broad Institute of Harvard and the Massachusetts Institute of Technology, Cambridge, MA USA; 14 Laboratory of Clinical Genome Sequencing, Department of Computational Biology and Medical Sciences, Graduate School of Frontier Sciences, The University of Tokyo, Tokyo, Japan; 15 Department of Public Health and Institute of Public Health, Chung Shan Medical University, Taichung, Taiwan; 16 Department of Internal Medicine, University of Turku, Turku, Finland; 17 Department of Public Health and Welfare, Finnish Institute for Health and Welfare, Turku, Finland; 18 Nuffield Department of Population Health, University of Oxford, Oxford, UK; 19 Department of Research and Innovation, Division of Clinical Neuroscience, Oslo University Hospital, Oslo, Norway; 20 K. G. Jebsen Center for Genetic Epidemiology, Department of Public Health and Nursing, Faculty of Medicine and Health Sciences, Norwegian University of Science and Technology (NTNU), Trondheim, Norway; 21 Department of Neurology, Oslo University Hospital, Oslo, Norway; 22 Department of Biostatistics, School of Public Health, University of Alabama at Birmingham, Birmingham, AL USA; 23 Department of Neurology and Cerebrovascular Disease Center, Seoul National University Bundang Hospital, Seoul National University College of Medicine, Seongnam, Republic of Korea; 24 Rajendra Institute of Medical Sciences, Ranchi, India; 25 Thrombosis and Atherosclerosis Research Institute, David Braley Cardiac, Vascular and Stroke Research Institute, Hamilton, Ontario Canada; 26 Department of Pathology and Molecular Medicine, Michael G. DeGroote School of Medicine, McMaster University, Hamilton, Ontario Canada; 27 Department of Neurology, Helsinki University Hospital and University of Helsinki, Helsinki, Finland; 28 Center for Genomic and Precision Medicine, College of Medicine, University of Ibadan, Ibadan, Nigeria; 29 Neuroscience and Ageing Research Unit Institute for Advanced Medical Research and Training, College of Medicine, University of Ibadan, Ibadan, Nigeria; 30 Department of Epidemiology, Erasmus MC University Medical Center Rotterdam, Rotterdam, The Netherlands; 31 Department of Radiology and Nuclear Medicine, Erasmus MC University Medical Center Rotterdam, Rotterdam, The Netherlands; 32 The Edmond and Lily Safra Center for Brain Sciences, The Hebrew University of Jerusalem, Jerusalem, Israel; 33 Department of Epidemiology, Harvard T. H. Chan School of Public Health, Boston, MA USA; 34 Department of Clinical Biochemistry, Copenhagen University Hospital—Rigshospitalet, Copenhagen, Denmark; 35 Department of Molecular and Functional Genomics, Weis Center for Research, Geisinger Health System, Danville, VA USA; 36 Department of Public Health Sciences, College of Medicine, The Pennsylvania State University, State College, PA USA; 37 Stroke Pharmacogenomics and Genetics Laboratory, Biomedical Research Institute Sant Pau (IIB Sant Pau), Barcelona, Spain; 38 Departament de Medicina, Universitat Autònoma de Barcelona, Barcelona, Spain; 39 The Danish Twin Registry, Department of Public Health, University of Southern Denmark, Odense, Denmark; 40 Department of Clinical Genetics, Odense University Hospital, Odense, Denmark; 41 Center for Alzheimer’s and Related Dementias, National Institutes of Health, Bethesda, MD USA; 42 Laboratory of Neurogenetics, National Institute on Aging, National Institutes of Health, Bethesda, MD USA; 43 Data Tecnica International, Glen Echo, MD USA; 44 Center for Public Health Genomics, University of Virginia, Charlottesville, VA USA; 45 Department of Biochemistry and Molecular Genetics, University of Virginia, Charlottesville, VA USA; 46 British Heart Foundation Cardiovascular Epidemiology Unit, Department of Public Health and Primary Care, University of Cambridge, Cambridge, UK; 47 Department of Genetics, Novo Nordisk Research Centre Oxford, Oxford, UK; 48 Department of Biomedical Informatics, Vanderbilt University Medical Center, Nashville, TN USA; 49 Department of Surgery, University of Pennsylvania, Philadelphia, PA USA; 50 Department of Medicine and Clinical Science, Graduate School of Medical Sciences, Kyushu University, Fukuoka, Japan; 51 University of Lille, INSERM U1167, RID-AGE, LabEx DISTALZ, Risk Factors and Molecular Determinants of Aging-Related Diseases, Lille, France; 52 CHU Lille, Public Health Department, Lille, France; 53 Institut Pasteur de Lille, Lille, France; 54 Department of Epidemiology, University of Alabama at Birmingham, Birmingham, AL USA; 55 UMC Utrecht Brain Center, Department of Neurology and Neurosurgery, University Medical Center Utrecht, University Utrecht, Utrecht, The Netherlands; 56 Cardiovascular Health Research Unit, Department of Medicine, University of Washington, Seattle, WA USA; 57 Department of Biostatistics, University of Washington, Seattle, WA USA; 58 Rush Alzheimer’s Disease Center, Rush University Medical Center, Chicago, IL USA; 59 Institute of Clinical Medicine, Faculty of Medicine, University of Oslo, Oslo, Norway; 60 Research and Communication Unit for Musculoskeletal Health (FORMI), Department of Research and Innovation, Division of Clinical Neuroscience, Oslo University Hospital, Oslo, Norway; 61 Division of Preventive Medicine, Brigham and Women’s Hospital, Boston, MA USA; 62 Harvard Medical School, Boston, MA USA; 63 MRC Population Health Research Unit, University of Oxford, Oxford, UK; 64 Department of Psychiatry, Washington University School of Medicine, Saint Louis, MO USA; 65 NeuroGenomics and Informatics Center, Washington University School of Medicine, Saint Louis, MO USA; 66 VA Maryland Health Care System, Baltimore, MD USA; 67 Department of Neurology, University of Maryland School of Medicine, Baltimore, MD USA; 68 Center for Translational and Computational Neuroimmunology, Department of Neurology, Columbia University Medical Center, New York, NY USA; 69 GenomesForLife—GCAT Lab Group, Germans Trias i Pujol Research Institute (IGTP), Badalona, Spain; 70 Klinik und Hochschulambulanz für Neurologie, Charité—Universitätsmedizin Berlin, Berlin, Germany; 71 Center for Stroke Research Berlin, Berlin, Germany; 72 German Center for Neurodegenerative Diseases (DZNE), partner site Berlin, Berlin, Germany; 73 German Centre for Cardiovascular Research (DZHK), partner site Berlin, Berlin, Germany; 74 Post-Graduation Program on Health and Environment, Department of Medicine and Joinville Stroke Biobank, University of the Region of Joinville, Santa Catarina, Brazil; 75 Department of Epidemiology, Julius Center for Health Sciences and Primary Care, University Medical Center Utrecht, Utrecht University, Utrecht, The Netherlands; 76 Icelandic Heart Association, Kopavogur, Iceland; 77 Faculty of Medicine, University of Iceland, Reykjavik, Iceland; 78 Department of Epidemiology and Public Health, Graduate School of Medical Sciences, Kyushu University, Fukuoka, Japan; 79 Department of Epidemiology and Biostatistics, School of Public Health, Imperial College London, London, UK; 80 Department of Public Health and Welfare, Finnish Institute for Health and Welfare, Helsinki, Finland; 81 Institute for Molecular Medicine Finland, FIMM-HiLIFE, Helsinki, Finland; 82 Clinical Trial Service and Epidemiological Studies Unit (CTSU), Nuffield Department of Population Health, University of Oxford, Oxford, UK; 83 Department of Neurology and Rehabilitation Medicine, University of Cincinnati College of Medicine, Cincinnati, OH USA; 84 Cambridge Baker Systems Genomics Initiative, Department of Public Health and Primary Care, University of Cambridge, Cambridge, UK; 85 Cambridge Baker Systems Genomics Initiative, Baker Heart and Diabetes Institute, Melbourne, Victoria Australia; 86 Health Data Research UK Cambridge, Wellcome Genome Campus and University of Cambridge, Cambridge, UK; 87 British Heart Foundation Centre of Research Excellence, University of Cambridge, Cambridge, UK; 88 Department of Neurology, Donders Center for Medical Neuroscience, Radboud University Medical Center, Nijmegen, The Netherlands; 89 Los Angeles County Department of Public Health, Los Angeles, CA USA; 90 Institute of Biomedicine, Department of Laboratory Medicine, the Sahlgrenska Academy, University of Gothenburg, Gothenburg, Sweden; 91 Department of Clinical Genetics and Genomics, Sahlgrenska University Hospital, Gothenburg, Sweden; 92 Department of Health Care Administration and Management, Graduate School of Medical Sciences, Kyushu University, Fukuoka, Japan; 93 Department of Biology, Brody School of Medicine Center for Health Disparities, East Carolina University, Greenville, NC USA; 94 Department of Neurology and Geriatric Research and Education Clinical Center, VA Maryland Health Care System, Baltimore, MD USA; 95 Department of Epidemiology and Preventive Medicine, School of Public Health and Preventive Medicine, Monash University, Melbourne, Victoria Australia; 96 Intramural Research Program, National Institute on Aging, NIH, Baltimore, MD USA; 97 Department of Neurology, Korea University Guro Hospital, Seoul, Republic of Korea; 98 Department of Computational Biology, University of Lausanne, Lausanne, Switzerland; 99 Swiss Institute of Bioinformatics, Lausanne, Switzerland; 100 University Center for Primary Care and Public Health, Lausanne, Switzerland; 101 Department of Epidemiology and Biostatistics, School of Public Health, Peking University Health Science Center, Beijing, China; 102 Stroke Research Group, Department of Clinical Neurosciences, University of Cambridge, Cambridge, UK; 103 Institute of Human Genetics, Technical University of Munich, Munich, Germany; 104 Institute of Human Genetics, Helmholtz Zentrum München, German Research Center for Environmental Health, Neuherberg, Germany; 105 Department of Medicine, University of Maryland School of Medicine, Baltimore, MD USA; 106 Geriatrics Research and Education Clinical Center, Baltimore Veterans Administration Medical Center, Baltimore, MD USA; 107 Institute of Clinical Epidemiology and Biometry, University of Würzburg, Würzburg, Germany; 108 Department of Neurology, University Hospital Würzburg, Würzburg, Germany; 109 The MIND Center, University of Mississippi Medical Center, Jackson, MS USA; 110 Department of Clinical Biochemistry, Copenhagen University Hospital—Herlev and Gentofte, Copenhagen, Denmark; 111 Department of Clinical Medicine, University of Copenhagen, Copenhagen, Denmark; 112 College of Medicine Nursing and Health Science, NUI Galway, Galway, Ireland; 113 Department of Genome Informatics, Graduate School of Medicine, The University of Tokyo, Tokyo, Japan; 114 Laboratory for Systems Genetics, RIKEN Center for Integrative Medical Sciences, Yokohama, Japan; 115 Laboratory of Statistical Immunology, Immunology Frontier Research Center (WPI-IFReC), Osaka University, Suita, Japan; 116 Integrated Frontier Research for Medical Science Division, Institute for Open and Transdisciplinary Research Initiatives, Osaka University, Suita, Japan; 117 Center for Infectious Disease Education and Research (CiDER), Osaka University, Suita, Japan; 118 Weill Institute for Neurosciences, University of California, San Francisco, San Francisco, CA USA; 119 Institute of Epidemiology, Helmholtz Zentrum München,, German Research Center for Environmental Health, Neuherberg, Germany; 120 Institute for Medical Information Processing, Biometry and Epidemiology, Ludwig Maximilian University Munich, Munich, Germany; 121 German Centre for Cardiovascular Research (DZHK), partner site Munich, Munich, Germany; 122 Department of Epidemiology, University of Washington, Seattle, WA USA; 123 Department of Health Systems and Population Health, University of Washington, Seattle, WA USA; 124 McCance Center for Brain Health, Massachusetts General Hospital, Boston, MA USA; 125 Department of Neurology, University of Miami Miller School of Medicine, Miami, FL USA; 126 Evelyn F. McKnight Brain Institute, Gainesville, FL USA; 127 Division of Cardiology, Department of Medicine, Columbia University, New York, NY USA; 128 Stroke Unit, Department of Neurology, Oslo University Hospital, Oslo, Norway; 129 Research and Development, The Norwegian Air Ambulance Foundation, Oslo, Norway; 130 Glenn Biggs Institute for Alzheimer’s and Neurodegenerative Diseases, University of Texas Health Sciences Center, San Antonio, TX USA; 131 Framingham Heart Study, Framingham, MA USA; 132 University Medicine Greifswald, Institute for Community Medicine, SHIP/KEF, Greifswald, Germany; 133 Kaiser Permanente Washington Health Research Institute, Kaiser Permanente Washington, Seattle, WA USA; 134 Department of Veterans Affairs Office of Research and Development, Seattle Epidemiologic Research and Information Center, Seattle, WA USA; 135 Department of Neurology, University of Pennsylvania, Philadelphia, PA USA; 136 Atlanta VA Health Care System, Decatur, GA USA; 137 Department of Epidemiology, Emory University Rollins School of Public Health, Atlanta, GA USA; 138 Department of Clinical Neuroscience, Institute of Neuroscience and Physiology, Sahlgrenska Unviersity Hospital, Gothenburg, Sweden; 139 Department of Internal Medicine, Section of Gerontology and Geriatrics, Leiden University Medical Center, Leiden, The Netherlands; 140 Department of Cardiology, Leiden University Medical Center, Leiden, The Netherlands; 141 Division Heart & Lungs, Department of Cardiology, University Medical Center Utrecht, Utrecht University, Utrecht, The Netherlands; 142 Department of Neurology and Neurosurgery, University of Tartu, Tartu, Estonia; 143 Department of Pathology and Laboratory Medicine, University of Pennsylvania, Philadelphia, PA USA; 144 Department of Public Health and Clinical Medicine, Umeå University, Umeå, Sweden; 145 Department of Medicine, Division of Cardiovascular Disease, Emory University School of Medicine, Atlanta, GA USA; 146 Department of Biostatistics, Boston University School of Public Health, Boston, MA USA; 147 Division of Genome Science, Department of Precision Medicine, National Institute of Health, Cheongju, Republic of Korea; 148 Research Unit Molecular Epidemiology, Institute of Epidemiology, Helmholtz Zentrum München, German Research Center for Environmental Health, Neuherberg, Germany; 149 Department of Psychiatry and Psychotherapy, University Medicine Greifswald, Greifswald, Germany; 150 German Center for Neurodegenerative Diseases (DZNE), site Rostock/Greifswald, Rostock, Germany; 151 Netherlands Heart Institute, Utrecht, The Netherlands; 152 Einthoven Laboratory for Experimental Vascular Medicine, LUMC, Leiden, The Netherlands; 153 Department of Clinical Genetics, Department of Radiology and Nuclear Medicine, Erasmus MC, Rotterdam, The Netherlands; 154 Latin American Brain Health (BrainLat), Universidad Adolfo Ibáñez, Santiago, Chile; 155 Department of Epidemiology and Population Health, Albert Einstein College of Medicine, New York, NY USA; 156 Department of Clinical Biochemistry and Pharmacology, Odense University Hospital, Odense, Denmark; 157 Department of Neurology, University of Virginia, Charlottesville, VA USA; 158 Department of Public Health Science, University of Virginia, Charlottesville, VA USA; 159 McGill Genome Centre, Montreal, Quebec Canada; 160 Longitudinal Studies Section, Translational Gerontology Branch, National Institute on Aging, Baltimore, MD USA; 161 Geisinger Neuroscience Institute, Geisinger Health System, Danville, PA USA; 162 Department of Neurology, College of Medicine, The Pennsylvania State University, State College, PA USA; 163 Klinik für Neurologie, Carl von Ossietzky University of Oldenburg, Oldenburg, Germany; 164 Comprehensive Heart Failure Center, University Hospital Würzburg, Würzburg, Germany; 165 Clinical Trial Center, University Hospital Würzburg, Würzburg, Germany; 166 Department of Neurology, University of Washington, Seattle, WA USA; 167 Institute for Health Promotion, Graduate School of Public Health, Yonsei University, Seoul, Republic of Korea; 168 Department of Health Research Methods, Evidence and Impact, McMaster University, Hamilton, Ontario Canada; 169 Population Health Research Institute, David Braley Cardiac, Vascular and Stroke Research Institute, Hamilton, Ontario Canada; 170 Department of Surgery and Department of Genetics, University of Pennsylvania, Philadelphia, PA USA; 171 Corporal Michael Crescenz VA Medical Center, Philadelphia, PA USA; 172 The Institute for Translational Genomics and Population Sciences, Department of Pediatrics, The Lundquist Institute for Biomedical Innovation at Harbor-UCLA Medical Center, Torrance, CA USA; 173 Department of Neurology, Brigham and Women’s Hospital, Boston, MA USA; 174 Division of Medicine, Turku University Hospital, Turku, Finland; 175 Brown Foundation Institute of Molecular Medicine, McGovern Medical School, University of Texas Health Science Center at Houston, Houston, TX USA; 176 Human Genetics Center, School of Public Health, University of Texas Health Science Center at Houston, Houston, TX USA; 177 Department of Medical Imaging, Chung Shan Medical University Hospital, Taichung, Taiwan; 178 Department of Neurology, Boston University School of Medicine, Boston, MA USA; 179 Department of Medicine, University of Ibadan, Ibadan, Nigeria; 180 Munich Cluster for Systems Neurology, Munich, Germany; 181 German Center for Neurodegenerative Diseases (DZNE), Munich, Germany; 182 Department of Neurology, Institute for Neurodegenerative Diseases, CHU de Bordeaux, Bordeaux, France; 183 Department of Neurology, Washington University School of Medicine, Saint Louis, MO USA; 184 Baltimore Veterans Administration Medical Center and University of Maryland School of Medicine, Baltimore, MD USA; 185 Mayo Clinic Florida, Jacksonville, FL USA; 186 Laboratory of Epidemiology and Population Science, National Institute on Aging, National Institutes of Health, Baltimore, MD USA; 187 University of Mississippi Medical Center, Jackson, MS USA; 188 William Harvey Research Institute, Barts and The London School of Medicine and Dentistry, Queen Mary University of London, London, UK; 189 Social, Genetic and Developmental Psychiatry Centre, King’s College London, London, UK; 190 Initiative for Research and Education to Advance Community Health, Washington State University, Seattle, WA USA; 191 Division of Public Health Sciences, Fred Hutchinson Cancer Research Center, Seattle, WA USA; 192 Division of Public Health Sciences, Wake Forest School of Medicine, Winston-Salem, NC USA; 193 Johns Hopkins University School of Medicine, Baltimore, MD USA; 194 Department of Neurology, Johns Hopkins University School of Medicine, Baltimore, MD USA; 195 Stroke Branch, National Institute of Neurological Disorders and Stroke, Bethesda, MD USA; 196 University of California, San Francisco, San Francisco, CA USA; 197 University of Colorado Anschutz Medical Campus, Denver, CO USA; 198 Brown University Warren Alpert Medical School, Providence, RI USA; 199 College of Public Health, University of Kentucky, Lexington, KY USA; 200 University of British Columbia, Vancouver, British Columbia Canada; 201 Neuroscience Institute, Saint Francis Medical Center, Trenton, NJ USA; 202 HUNT Research Center, Department of Public Health and Nursing, Faculty of Medicine and Health Sciences, Norwegian University of Science and Technology (NTNU), Trondheim, Norway; 203 Department of Research, Innovation and Education, St Olavs Hospital, Trondheim University Hospital, Trondheim, Norway; 204 Department of Environmental and Occupational Health Sciences, University of Washington, Seattle, WA USA; 205 Department of Pediatrics and Rady Children’s Hospital, University of California San Diego, La Jolla, CA USA; 206 K. G. Jebsen Thrombosis Research and Expertise Center, Department of Clinical Medicine, UiT—The Arctic University of Norway, Tromsø, Norway; 207 Department of Clinical Epidemiology, Leiden University Medical Center, Leiden, The Netherlands; 208 Department of Health Sciences Research, Mayo Clinic, Rochester, MN USA; 209 Division of Biostatistics, School of Public Health, University of Minnesota, Minneapolis, MN USA; 210 MRC Integrative Epidemiology Unit, University of Bristol, Bristol, UK; 211 Clinic of Thoracic and Occupational Medicine, St Olavs Hospital, Trondheim University Hospital, Trondheim, Norway; 212 Laboratory of Haematology, La Timone Hospital, Marseille, France; 213 C2VN, University of Aix Marseille, INSERM, INRAE, C2VN, Marseille, France; 214 Institute of Genomic Medicine, University of California, San Diego, La Jolla, CA USA; 215 Program in Genetic Epidemiology and Statistical Genetics, Harvard T. H. Chan School of Public Health, Boston, MA USA; 216 Division of Internal Medicine, University Hospital of North Norway, Tromsø, Norway; 217 Department of Laboratory Medicine and Pathology, School of Medicine, University of Minnesota, Minneapolis, MN USA; 218 Division of Endocrinology, Diabetes and Metabolism, The Ohio State University, Columbus, OH USA; 219 Department of Internal Medicine and the Center for Clinical and Translational Science, Ohio State University, Columbus, OH USA; 220 Department of Internal Medicine, Division of Cardiology, University of Michigan, Ann Arbor, MI USA; 221 Department of Epidemiology Research, Statens Serum Institut, Copenhagen, Denmark; 222 Institute for Translational Genomics and Population Sciences, Los Angeles Biomedical Research Institute at Harbor–UCLA Medical Center, Torrance, CA USA; 223 Division of Genomic Outcomes, Department of Pediatrics, Harbor–UCLA Medical Center, Torrance, CA USA; 224 Division of Epidemiology and Community Health, School of Public Health, University of Minnesota, Minneapolis, MN USA; 225 Centre National de Recherche en Génomique Humaine, Direction de la Recherche Fondamentale, CEA, Evry, France; 226 CEPH-Fondation Jean Dausset, Paris, France; 227 Million Veteran’s Program, Veteran’s Administration, Boston, MA USA; 228 CRB Assistance Publique—Hôpitaux de Marseille, HemoVasc (CRB AP-HM HemoVasc), Marseille, France; 229 Center for Vascular Emergencies, Department of Emergency Medicine, Massachusetts General Hospital, Boston, MA USA; 230 Department of Emergency Medicine, Harvard Medical School, Boston, MA USA; 231 Department of Neurology, Erasmus University Medical Center, Rotterdam, The Netherlands; 232 Department of Neurology, University Medical Center Groningen, Groningen, The Netherlands; 233 Department of Neurology, Amsterdam UMC, University of Amsterdam, Amsterdam, The Netherlands; 234 Department of Neurology, Cardiovascular Research Institute Maastricht, Maastricht University Medical Center, Maastricht, The Netherlands; 235 Department of Neurology, Leiden University Medical Center, Leiden, The Netherlands; 236 John Hunter Hospital, Hunter Medical Research Institute and University of Newcastle, Newcastle, New South Wales Australia; 237 Faculty of Health, University of Technology Sydney, Ultimo, New South Wales Australia; 238 Department of Neurology, IMIM-Hospital del Mar, Neurovascular Research Group, IMIM (Institut Hospital del Mar d’Investigacions Mèdiques), Universitat Autònoma de Barcelona/DCEXS-Universitat Pompeu Fabra, Barcelona, Spain; 239 Institute of Cardiovascular Research, Royal Holloway University of London, London, UK; 240 Ashford and St Peters Hospital, Chertsey, UK; 241 Usher Institute of Population Health Sciences and Informatics, University of Edinburgh, Edinburgh, UK; 242 Centre for Clinical Brain Sciences, University of Edinburgh, Edinburgh, UK; 243 Department of Neurology, Medical University of Graz, Graz, Austria; 244 Department of Neurology, Jagiellonian University, Krakow, Poland; 245 Stroke Division, Florey Institute of Neuroscience and Mental Health, University of Melbourne, Heidelberg, Melbourne, Victoria Australia; 246 Department of Neurology, Austin Health, Heidelberg, Victoria Australia; 247 Department of Clinical Sciences Lund, Neurology, Lund University, Lund, Sweden; 248 Department of Neurology, Rehabilitation Medicine, Memory Disorders, Geriatrics, Skåne University Hospital, Lund, Sweden; 249 Department of Clinical Sciences, Lund University, Malmö, Sweden; 250 Department of Medicine, Brigham and Women’s Hospital, Boston, MA USA; 251 Wolfson Centre for Prevention of Stroke and Dementia, Nuffield Department of Clinical Neurosciences, University of Oxford, Oxford, UK; 252 Department of Pharmacotherapy and Translational Research and Center for Pharmacogenomics, College of Pharmacy, University of Florida, Gainesville, FL USA; 253 Division of Cardiovascular Medicine, College of Medicine, University of Florida, Gainesville, FL USA; 254 BHF Cardiovascular Epidemiology Unit, Department of Public Health and Primary Care, University of Cambridge, Cambridge, UK; 255 NIHR Blood and Transplant Research Unit in Donor Health and Genomics, Department of Public Health and Primary Care, University of Cambridge, Cambridge, UK; 256 Wellcome Trust Sanger Institute, Cambridge, UK; 257 British Heart Foundation Cambridge Centre of Excellence, Department of Medicine, University of Cambridge, Cambridge, UK; 258 National Institute for Health Research Blood and Transplant Research Unit in Donor Health and Genomics, University of Cambridge, Cambridge, UK; 259 Department of Emergency Medicine, Washington University School of Medicine, Saint Louis, MO USA; 260 Department of Cerebrovascular Diseases, Fondazione IRCCS Istituto Neurologico ‘Carlo Besta’, Milan, Italy; 261 Laboratory for Statistical Analysis, RIKEN Center for Integrative Medical Sciences, Yokohama, Japan; 262 Department of Clinical and Experimental Sciences, Neurology Clinic, University of Brescia, Brescia, Italy; 263 Albrecht Kossel Institute, University Clinic of Rostock, Rostock, Germany; 264 Department of Neurology, Massachusetts General Hospital (MGH), Harvard Medical School, Boston, MA USA; 265 Survey Research Center, University of Michigan, Ann Arbor, MI USA; 266 Department of Neurosciences, Experimental Neurology, KU Leuven–University of Leuven, Leuven, Belgium; 267 Department of Neurology, VIB Center for Brain & Disease Research, University Hospitals Leuven, Leuven, Belgium; 268 Department of Medicine, Larner College of Medicine at the University of Vermont, Burlington, VT USA; 269 Department of Clinical Neuroscience, Institute of Neuroscience and Physiology, Sahlgrenska Academy at University of Gothenburg, Gothenburg, Sweden; 270 Region Västra Götaland, Department of Neurology, Sahlgrenska University Hospital, Gothenburg, Sweden; 271 Chang Gung Memorial Hospital, Linkou Medical Center in Taiwan, Taoyuan City, Taiwan; 272 Department of Neurology, School of Medicine, University of Pittsburgh, Pittsburgh, PA USA; 273 Department of Neurology, University Medicine Greifswald, Greifswald, Germany; 274 Stroke Pharmacogenomics and Genetics Laboratory, Fundación Docència I Recerca Mútua Terrassa, Hospital Mútua Terrassa, Terrassa, Spain; 275 Neurovascular Research Laboratory, Vall d’Hebron Institute of Research, Universitat Autònoma de Barcelona, Barcelona, Spain; 276 Neurology Service, Hospital Universitari Mútua Terrassa, Terrassa, Spain; 277 Department of Neurology, Beth Israel Deaconess Medical Center, Boston, MA USA; 278 Institute de Biomedicine of Seville, IBiS/Hospital Universitario Virgen del Rocío/CSIC/University of Seville and Department of Neurology, Hospital Universitario Virgen Macarena, Seville, Spain; 279 Department of Epidemiology, Human Genetics, and Environmental Sciences (EHGES), UTHealth Science Center, School of Public Health, Houston, TX USA; 280 BHF Glasgow Cardiovascular Research Centre, Faculty of Medicine, University of Glasgow, Glasgow, UK; 281 German Centre for Cardiovascular Research (DZHK), partner site Greifswald, Greifswald, Germany; 282 Program in Bioinformatics and Integrative Genomics, Harvard Medical School, Boston, MA USA; 283 Department of Statistical Genetics, Osaka University Graduate School of Medicine, Osaka, Japan; 284 deCODE genetics/AMGEN, Reykjavik, Iceland; 285 J. Philip Kistler Stroke Research Center, Department of Neurology, MGH, Boston, MA USA; 286 Population Health Research Institute, McMaster University, Hamilton, Ontario Canada; 287 AA Martinos Center for Biomedical Imaging, Department of Radiology, MGH, Harvard Medical School, Boston, MA USA; 288 School of Life Science, University of Lincoln, Lincoln, UK; 289 Department of Neurology, Mayo Clinic, Rochester, MN USA; 290 Stroke Pharmacogenomics and Genetics, Fundacio Docència i Recerca MutuaTerrassa, Terrassa, Spain; 291 Department of Medical Genetics, University Medical Center Utrecht, Utrecht, The Netherlands; 292 Boston University School of Public Health, Boston, MA USA; 293 The Beijer Laboratory and Department of Immunology, Genetics and Pathology, Uppsala University and Science for Life Laboratory, Uppsala, Sweden; 294 Department of Genetics, University of North Carolina, Chapel Hill, NC USA; 295 Department of Neurology and Stroke Center, Basel University Hospital, Basel, Switzerland; 296 Neurorehabilitation Unit, University of Basel and University Center for Medicine of Aging and Rehabilitation Basel, Felix Platter Hospital, Basel, Switzerland; 297 Department of Neurology, Yale University School of Medicine, New Haven, CT USA; 298 Department of Medical Sciences, Molecular Epidemiology and Science for Life Laboratory, Uppsala University, Uppsala, Sweden; 299 Department of Neurology, Leeds General Infirmary, Leeds Teaching Hospitals NHS Trust, Leeds, UK; 300 Public Health Stream, Hunter Medical Research Institute, New Lambton, New South Wales Australia; 301 College of Health, Medicine and Wellbeing, The University of Newcastle, Newcastle, New South Wales Australia; 302 Department of Biostatistics and Data Science, Division of Public Health Sciences, Wake Forest University School of Medicine, Winston-Salem, NC USA; 303 Department of Medicine, Division of Cardiovascular Medicine, Stanford University School of Medicine, Stanford, CA USA; 304 MRC Epidemiology Unit, School of Clinical Medicine, Institute of Metabolic Science, University of Cambridge, Cambridge, UK; 305 Computational Medicine, Berlin Institute of Health at Charité – Universitätsmedizin Berlin, Berlin, Germany; 306 Precision Healthcare Institute, Queen Mary University of London, London, UK; 307 INSERM U 1172, CHU Lille, Université Lille, Lille, France; 308 MRC Biostatistics Unit, University of Cambridge, Cambridge, UK; 309 Bioinformatics Core Facility, University of Gothenburg, Gothenburg, Sweden; 310 Department of Medical Epidemiology and Biostatistics, Karolinska Institutet, Stockholm, Sweden; 311 Department of Allergy and Rheumatology, Graduate School of Medicine, University of Tokyo, Tokyo, Japan; 312 Department of Pediatrics, College of Medicine, University of Oklahoma Health Sciences Center, Oklahoma City, OK USA; 313 Department of Neurology, Justus Liebig University, Giessen, Germany; 314 Department of Public Health, Bordeaux University Hospital, Bordeaux, France; 315 School of Medicine, Dentistry and Nursing at the University of Glasgow, Glasgow, UK; 316 University of Newcastle and Hunter Medical Research Institute, New Lambton, New South Wales Australia; 317 INM, University of Montpellier Inserm U1298, Montpellier, France; 318 Centre for Research in Environmental Epidemiology, Barcelona, Spain; 319 Department of Neurology, Università degli Studi di Perugia, Umbria, Italy; 320 Broad Institute, Cambridge, MA USA; 321 Department of Neurology, Memory Clinic, Bordeaux University Hospital, Bordeaux, France; 322 Department of Internal Medicine B, University Medicine Greifswald, Greifswald, Germany; 323 Robertson Center for Biostatistics, University of Glasgow, Glasgow, UK; 324 Hero DMC Heart Institute, Dayanand Medical College & Hospital, Ludhiana, India; 325 Atherosclerosis Research Unit, Department of Medicine Solna, Karolinska Institutet, Stockholm, Sweden; 326 Tohoku Medical Megabank Organization, Sendai, Japan; 327 Department of Public Health and Caring Sciences/Geriatrics, Uppsala University, Uppsala, Sweden; 328 Krembil Brain Institute, University Health Network, Toronto, Ontario Canada; 329 Department of Medicine and Tanz Centre for Research in Neurodegenerative Diseases, University of Toronto, Toronto, Ontario Canada; 330 Epidemiology and Prevention Group, Center for Public Health Sciences, National Cancer Center, Tokyo, Japan; 331 Department of Basic and Clinical Neurosciences, King’s College London, London, UK; 332 Departments of Neurology & Radiology, Landspitali National University Hospital, Reykjavik, Iceland; 333 Department of Neurology, Heidelberg University Hospital, Heidelberg, Germany; 334 Montefiore Medical Center, Albert Einstein College of Medicine, New York, NY USA; 335 Department of Medical Sciences, Uppsala University, Uppsala, Sweden; 336 Genetic and Genomic Epidemiology Unit, Wellcome Trust Centre for Human Genetics, University of Oxford, Oxford, UK; 337 Wellcome Trust Centre for Human Genetics, Oxford, UK; 338 BioBankJapan, Laboratory of Clinical Sequencing, Department of Computational Biology and Medical Sciences, Graduate School of Frontier Sciences, University of Tokyo, Tokyo, Japan; 339 Department of Biostatistics, University of Liverpool, Liverpool, UK; 340 Institute of Genetic Epidemiology, Helmholtz Zentrum München—German Research Center for Environmental Health, Neuherberg, Germany; 341 Department of Medicine I, Ludwig-Maximilians-Universität, Munich, Germany; 342 Translational Genomics Unit, Department of Oncology, IRCCS Istituto di Ricerche Farmacologiche Mario Negri, Milan, Italy; 343 Department of Genetics, Microbiology and Statistics, University of Barcelona, Barcelona, Spain; 344 Psychiatric Genetics Unit, Group of Psychiatry, Mental Health and Addictions, Vall d’Hebron Research Institute (VHIR), Universitat Autònoma de Barcelona,Biomedical Network Research Centre on Mental Health (CIBERSAM), Barcelona, Spain; 345 National Institute for Health Research Comprehensive Biomedical Research Centre, Guy’s & St Thomas’ NHS Foundation Trust and King’s College London, London, UK; 346 School of Lifecourse and Population Sciences, King’s College London, London, UK; 347 Icelandic Heart Association, Reykjavik, Iceland; 348 Department of Epidemiology, University of Maryland School of Medicine, Baltimore, MD USA; 349 Institute of Cardiovascular and Medical Sciences, College of Medical, Veterinary and Life Sciences, University of Glasgow, Glasgow, UK; 350 IBE, Faculty of Medicine, LMU Munich, Munich, Germany; 351 Division of Epidemiology and Prevention, Aichi Cancer Center Research Institute, Nagoya, Japan; 352 Department of Epidemiology, Nagoya University Graduate School of Medicine, Nagoya, Japan; 353 Department of Neurology, Caen University Hospital, Caen, France; 354 University of Caen Normandy, Caen, France; 355 Department of Internal Medicine, Erasmus University Medical Center, Rotterdam, The Netherlands; 356 Landspitali University Hospital, Reykjavik, Iceland; 357 Department of Pharmaceutical Sciences, College of Pharmacy, University of Oklahoma Health Sciences Center, Oklahoma City, OK USA; 358 Oklahoma Center for Neuroscience, Oklahoma City, OK USA; 359 IMIM (Hospital del Mar Medical Research Institute), Barcelona, Spain; 360 Department of Epidemiology and Medical Statistics, University of Ibadan, Ibadan, Nigeria; 361 Institute of Cardiovascular Diseases, University of Ibadan, Ibadan, Nigeria; 362 Department of Medicine, Kwame Nkrumah University of Science and Technology, Kumasi, Ghana; 363 Department of Medicine, University of Ghana Medical School, Accra, Ghana; 364 Department of Medicine, Ahmadu Bello University, Zaria, Nigeria; 365 Department of Medicine, University of Ilorin Teaching Hospital, Ilorin, Nigeria; 366 Jos University Teaching Hospital, Jos, Nigeria; 367 Department of Medicine, Aminu Kano Teaching Hospital, Kano, Nigeria; 368 Department of Medicine, Obafemi Awolowo University Teaching Hospital, Ile-Ife, Nigeria; 369 Medical University of South Carolina, Charleston, SC USA; 370 Department of Health Promotion and Education, University of Ibadan, Ibadan, Nigeria; 371 Department of Radiology, College of Medicine, University of Ibadan, Ibadan, Nigeria; 372 China National Center For Food Safety Risk Assessment, Beijing, China; 373 Fuwai Hospital, Chinese Academy of Medical Sciences, National Center for Cardiovascular Diseases, Beijing, China; 374 Chinese Academy of Medical Sciences, Beijing, China; 375 Peking Union Medical College, Beijing, China; 376 Department of Public Policy, Institute of Medical Sciences, The University of Tokyo, Tokyo, Japan; 377 Human Genome Sequencing Center, Baylor College of Medicine, Houston, TX USA; 378 Interfaculty Institute for Genetics and Functional Genomics, University Medicine Greifswald, Greifswald, Germany; 379 Department of Neurology, Haga Hospital, The Hague, The Netherlands; 380 Department of Neurology, Amphia Hospital, Breda, The Netherlands; 381 Department of Neurology, Elisabeth-Tweesteden Hospital, Tilburg, The Netherlands; 382 Department of Neurology, Rijnstate Hospital, Arnhem, The Netherlands; 383 Department of Neurology, Medisch spectrum Twente, Enschede, The Netherlands; 384 Department of Neurology, Catharina Hospital, Eindhoven, The Netherlands; 385 Department of Neurology, Franciscus Gasthuis & Vlietland, Rotterdam, The Netherlands; 386 Department of Neurology, Catharina Wilhelmina Hospital, Nijmegen, The Netherlands; 387 Department of Neurology, Medisch Center Leeuwarden, Leeuwarden, The Netherlands; 388 Department of Neuromedicine and Movement Science, Faculty of Medicine and Health Sciences, Norwegian University of Science and Technology (NTNU), Trondheim, Norway; 389 Department of Human Genetics, University of Michigan, Ann Arbor, MI USA; 390 Center for Statistical Genetics, Department of Biostatistics, University of Michigan, Ann Arbor, MI USA; 391 Stroke Unit, Department of Internal Medicine, St Olavs Hospital, Trondheim University Hospital, Trondheim, Norway; 392 Department of Computational Medicine and Bioinformatics, University of Michigan, Ann Arbor, MI USA; 393 Analytic and Translational Genetics Unit, Massachusetts General Hospital, Boston, MA USA; 394 Department of General Practice, University of Oslo, Oslo, Norway; 395 Department of Neurology, Akershus University Hospital, Lørenskog, Norway; 396 BioCore—Bioinformatics Core Facility, Norwegian University of Science and Technology (NTNU), Trondheim, Norway; 397 Clinic of Laboratory Medicine, St Olavs Hospital, Trondheim University Hospital, Trondheim, Norway; 398 Department of Clinical and Molecular Medicine, Norwegian University of Science and Technology (NTNU), Trondheim, Norway; 399 Department of Neurology and Center for Translational Neuro- and Behavioral Sciences (C-TNBS), University Hospital Essen, University Duisburg-Essen, Essen, Germany; 400 Department of Medicine I, University Hospital Würzburg, Würzburg, Germany; 401 Stroke Unit, Department of Neurology, Biomedical Research Institute Sant Pau, Hospital de la Santa Creu i Sant Pau, Barcelona, Spain; 402 Institute for Biomedical Research of Barcelona (IIBB), National Spanish Research Council (CSIC), Barcelona, Spain; 403 Clinical Neurosciences Research Laboratory (LINC), Health Research Institute of Santiago de Compostela (IDIS), Santiago de Compostela, Spain; 404 Department of Neurology, Biocruces-Bizkaia Health Research Institute, Bilbao, Spain; 405 Stroke Unit, Department of Neurology, University Hospital of Valladolid, Valladolid, Spain; 406 Department of Neurology, Hospital Clínic de Barcelona, IDIBAPS, Barcelona, Spain; 407 Stroke Unit, Department of Neurology, Hospital Universitari Vall d’Hebron, Barcelona, Spain; 408 Department of Neurosciences, Hospital Germans Trias I Pujol, Universitat Autònoma de Barcelona, Barcelona, Spain; 409 Department of Neurology, University Hospital Central de Asturias (HUCA), Oviedo, Spain; 410 Department of Neurology, Son Espases University Hospital, Illes Balears Health Research Institute (IdISBa), Palma, Spain; 411 Department of Neurology, University Hospital of Albacete, Albacete, Spain; 412 High Content Genomics and Bioinformatics Unit, Germans Trias i Pujol Research Institute (IGTP), Badalona, Spain; 413 The Copenhagen City Heart Study, Copenhagen University Hospital—Bispebjerg and Frederiksberg, Copenhagen, Denmark; 414 Central Diagnostics Laboratory, Division Laboratories, Pharmacy, and Biomedical genetics, University Medical Center Utrecht, Utrecht University, Utrecht, The Netherlands; 415 Taub Institute for Research on Alzheimer’s Disease and the Aging Brain, College of Physicians and Surgeons, Columbia University, New York, NY USA; 416 The Centre for Translational and Computational Neuroimmunology, Columbia University Medical Center, New York, NY USA; 417 Department of Human Genetics, McGill University, Montreal, Quebec Canada

**Keywords:** Stroke, Genome-wide association studies, Stroke, Predictive markers, Genetic markers

Correction to: *Nature* 10.1038/s41586-022-05165-3 Published online 30 September 2022

In the version of this article initially published, the name of the PRECISE4Q Consortium was misspelled as “PRECISEQ” and has now been amended in the HTML and PDF versions of the article. Further, data in the first column of Supplementary Table 55 were mistakenly shifted and have been corrected in the file accompanying the HTML version of the article.

## Supplementary information

Supplementary Information Supplementary Methods, including a description of the GWAS of stroke-risk factors in Tohuku Medical Megabank and Calculation of candidate polygenic score models; description of study populations in the GIGASTROKE initiative; study-specific acknowledgements; information on other members of participating consortia; and Supplementary References.

Supplementary Tables Supplementary Tables 1–56.

