# Supplementary material for: Stroke genetics informs drug discovery and risk prediction across ancestries
Source: Nature. 2022 Sep 30;611(7934):115–23. doi: 10.1038/s41586-022-05165-3 (PMC9524349; doi:10.1038/s41586-022-05165-3)
Supplement: Supplementary file 2 — Reporting Summary [file 41586_2022_5165_MOESM2_ESM.pdf]

nature portfolio

Corresponding author(s): Stephanie Debette and Martin Dichgans

Last updated by author(s): Jun 17, 2022

Reporting Summary

Nature Portfolio wishes to improve the reproducibility of the work that we publish. This form provides structure for consistency and transparency in reporting. For further information on Nature Portfolio policies, see our Editorial Policies and the Editorial Policy Checklist.

Statistics

For all statistical analyses, confirm that the following items are present in the figure legend, table legend, main text, or Methods section.

n/a	Confirmed	
☐	☒	The exact sample size (*n*) for each experimental group/condition, given as a discrete number and unit of measurement
☐	☒	A statement on whether measurements were taken from distinct samples or whether the same sample was measured repeatedly
☐	☒	The statistical test(s) used AND whether they are one- or two-sided<br>*Only common tests should be described solely by name; describe more complex techniques in the Methods section.*
☐	☒	A description of all covariates tested
☐	☒	A description of any assumptions or corrections, such as tests of normality and adjustment for multiple comparisons
☐	☒	A full description of the statistical parameters including central tendency (e.g. means) or other basic estimates (e.g. regression coefficient) AND variation (e.g. standard deviation) or associated estimates of uncertainty (e.g. confidence intervals)
☐	☒	For null hypothesis testing, the test statistic (e.g. *F*, *t*, *r*) with confidence intervals, effect sizes, degrees of freedom and *P* value noted<br>*Give P values as exact values whenever suitable.*
☒	☐	For Bayesian analysis, information on the choice of priors and Markov chain Monte Carlo settings
☒	☐	For hierarchical and complex designs, identification of the appropriate level for tests and full reporting of outcomes
☐	☒	Estimates of effect sizes (e.g. Cohen's *d*, Pearson's *r*), indicating how they were calculated

Our web collection on statistics for biologists contains articles on many of the points above.

Software and code

Policy information about availability of computer code

Data collection	No software was used for data collection
Data analysis	Each individual study that contributed genetic-phenotype association summary statistics to the consortium carried out their association analyses independently of the consortium (study-specific information outlined in Supplementary Table 2). Please refer Supplementary Table 2 for the software used for quality control, imputation and GWAS analyses by individual studies. Moreover we used publicaly available METAL v2020-05-05, MR-MEGA v.0.1.6 and MTAG v1.0.8 software for GWAS meta-analysis. For post GWAS analysis we used VEGASv2, MAGMA v1.08, cov-LDSC v1.0.0, GCTA-COJO v1.26.0, susieR, R v4.1.1 (MendelianRandomization package), MENTR v1, R v4.1.1 (TWAS-Fusion package), STEAP pipeline v1, GREP v1.0.0, Trans-Phar v1, R v4.0.3 (PheWAS package), plink v1.90b6.8, LDpred v.1.0.11, PRScs.py v2021-01-04. These are publically available software, original manuscript of these software was cited whenever these software were menioned in the manuscript

For manuscripts utilizing custom algorithms or software that are central to the research but not yet described in published literature, software must be made available to editors and reviewers. We strongly encourage code deposition in a community repository (e.g. GitHub). See the Nature Portfolio guidelines for submitting code & software for further information.

Data

Policy information about availability of data

All manuscripts must include a data availability statement. This statement should provide the following information, where applicable:

- Accession codes, unique identifiers, or web links for publicly available datasets
- A description of any restrictions on data availability
- For clinical datasets or third party data, please ensure that the statement adheres to our policy

Summary statistics generated by the GIGASTROKE consortium across ancestries and stroke subtypes are available in the GWAS Catalog (study code GCST90104534-

GCST90104563)
The integrated polygenic risk score models of stroke in Europeans and East Asians are available in the PGS Catalog (PGS002724 and PGS002725). Individual level data can be requested directly from contributing studies, listed in Supplementary Table 1. Single nucleus RNA-seq (snRNA-seq) data is deposited in the SYNAPSE database as part of the Religious Orders Study and Memory and Aging Project (ROSMAP) (https://www.synapse.org) and through the RADC Resource Sharing Hub (https://www.radc.rush.edu). We used publicly available data from GTEx (https://gtexportal.org/home/), the Gusev lab (http://gusevlab.org/projects/fusion/), the FinnGen Freeze 7 cohort (https://www.finngen.fi/en/access_results), PhenoScanner v2 database (http://www.phenoscanner.medschl.cam.ac.uk), the pQTL summary statistics (https://doi.org/10.1038/s41588-020-0682-6, http://www.phpc.cam.ac.uk/ceu/proteins/, http://metabolomics.helmholtz-muenchen.de/pgwas/index.php, https://zenodo.org/record/264128), the deCODE genetics (https://www.decode.com/summarydata/), the summary statistics using the UK Biobank whole-exome sequencing (https://doi.org/10.1038/s41586-021-04103-z).

Field-specific reporting

Please select the one below that is the best fit for your research. If you are not sure, read the appropriate sections before making your selection.

☒ Life sciences ☐ Behavioural & social sciences ☐ Ecological, evolutionary & environmental sciences

For a reference copy of the document with all sections, see nature.com/documents/nr-reporting-summary-flat.pdf

Life sciences study design

All studies must disclose on these points even when the disclosure is negative.

Sample size

We performed meta-analysis of GWAS on 29 population-based cohorts or biobanks with incident stroke ascertainment and 25 clinic-based case-control studies, comprising up to 110,182 stroke patients and 1,503,898 controls. We also gathered an independent dataset of 89,084 any stroke cases and 1,013,843 controls, mostly from large biobanks, for external replication. We included clinic-based studies with minimum of n=100 cases and n=100 controls, while we were more inclusive for population-based cohorts with longitudinal information on incident stroke and considered all population-based studies with more than 20 incident stroke cases. No statistical calculation for adequate sample size was performed, but the results identifying multiple genomic regions at genome-wide significance threshold indicates adequate power for genetic discovery.

Data exclusions

Individual level phenotype and genotype data exclusions were performed by each individual study, described in supplementary appendix. However in population-based longitudinal cohorts we considered well ascertained incident stroke cases only, the self-reported stroke cases at baseline were excluded from this analysis.

Replication

To verify the reproducibility of our findings, first, we replicated 42 loci discovered in one ancestry into the internal data of other ancestries (internal cross-ancestry validation). We successfully replicated 10 out of 42 loci after accounting for the number of loci tested, of which 7 were genome-wide significant in European (EUR), 1 in East Asian (EAS), and 2 in both (EUR) and (EAS). Additional 15 loci showed nominal association ($p<0.05$) in at least one other ancestry (Supplementary Table 15). Second, we also gathered an independent dataset of 89,084 any stroke cases and 1,013,843 controls, mostly from large biobanks, for external replication. Out of the 60 loci reaching genome-wide significance in primary inverse-variance weighted (IVW) meta-analyses, in this independent external dataset 48 loci (80%) replicated at $p<0.05$ with consistent directionality, of which 31 (52%), at $p<8.2\text{x}10^{-4}$ (accounting for the number of loci tested).
Based on these follow-up results we characterized the level of confidence of identified loci as follows: high confidence in case of significant internal 'cross-ancestry' and/or external replication after accounting for the number of loci tested, or nominally significant replication in both internal and external replication, or evidence of involvement in monogenic stroke; intermediate confidence in case of nominal significance in either internal 'inter-ancestry' or external replication but not both; and low confidence in the absence of formal replication.
Overall, out of the 60 loci reaching genome-wide significance in the main IVW GWAS meta-analysis, 52 (87%) replicated at $p<0.05$ with consistent direction, of which 37 (61.7%) with high confidence, and 15 with intermediate confidence (25%). The 8 loci that did not replicate were labeled as "low confidence". Four of these were ethnic specific and 3 were low frequency variants that were monomorphic in some ancestries, limiting our ability for replication.
Within the secondary analyses (MR-MEGA and MTAG), none of the 3 MR-MEGA loci replicated, although one was borderline significant (Supplementary Table 16). Of the 26 MTAG loci, 18 (69%) replicated with AS or AIS at $p<0.05$, of which 9 (35%) with high confidence. Of the 8 MTAG loci that did not replicate, 7 showed a consistent directionality (borderline significant for one), and 4 were subtype-specific, limiting our ability for replication with AS or AIS.
While we have clearly labeled "low confidence" variants, we have not removed them from bioinformatics functional follow-up analyses. Indeed, we feel that despite the important worldwide effort that enabled to gather nearly 90,000 additional stroke cases, several issues still affect our ability to replicate some of the identified stroke risk loci:
• limits of statistical power, considering a smaller sample size than in the discovery and the winner's curse phenomenon;
• we cannot rule out some degree of misclassification in the follow-up samples that were, with two smaller exceptions, nearly exclusively derived from large biobanks with stroke ascertainment based on ICD codes only (Turnbull, Lancet Reg Health West Pac. 2022; Rannikmae, Neurology 2020), while a large proportion of stroke cases in the discovery were recruited and deeply phenotyped in a hospital-based setting;
• a substantial proportion of genetic risk for stroke is subtype specific, which is not fully captured in the replication because of the limited availability of stroke subtype data

Randomization

No randomization was performed because there was no allocation of samples to experimental groups.

Blinding

Blinding was not relevant to this study. The investigators of each study evaluated the case status of individual samples. Individual studies performed a genome-wide association study (GWAS) using logistic regression (or cox regression in some longitudinal population-based cohorts) testing association of genotypes with five stroke phenotypes (AS, AIS, CES, LAS, and SVS) under an additive effect model, adjusting for age, sex, principal components of population stratification, and study specific covariates when needed, details are provided in Supplementary table 2. The consortium meta-analysed summary statistics from these case/control studies, not individual level data.

Reporting for specific materials, systems and methods

We require information from authors about some types of materials, experimental systems and methods used in many studies. Here, indicate whether each material, system or method listed is relevant to your study. If you are not sure if a list item applies to your research, read the appropriate section before selecting a response.

Materials & experimental systems

n/a	Involved in the study
☒	☐ Antibodies
☒	☐ Eukaryotic cell lines
☒	☐ Palaeontology and archaeology
☒	☐ Animals and other organisms
☐	☒ Human research participants
☒	☐ Clinical data
☒	☐ Dual use research of concern

Methods

n/a	Involved in the study
☒	☐ ChIP-seq
☒	☐ Flow cytometry
☒	☐ MRI-based neuroimaging

Human research participants

Policy information about studies involving human research participants

Population characteristics

We performed meta-analysis of GWAS on 29 population-based cohorts or biobanks with incident stroke ascertainment and 25 clinic-based case-control studies, comprising up to 110,182 stroke patients and 1,503,898 controls. The cohorts included individuals of European (EUR, 66.7% of stroke patients), East-Asian (EAS, 24.8%), African-American (AFR, 3.7%), South-Asian (SAS, 3.3%), and Hispanic (HIS, 1.4%) ancestry. Analyses were performed for any stroke (AS: comprising ischemic stroke, ICH, and stroke of unknown or undetermined type), any ischemic stroke regardless of subtype (AIS, N=86,668), and ischemic stroke subtypes (LAS, N=9,219; CES, N=12,790; SVS, N=13,620).
We also gathered an independent dataset of 89,084 AS (of which 85,546 AIS; 70.0% EUR, 15.6% AFR, 10.1% EAS, 4.1% HIS, and 0.1% SAS) and 1,013,843 controls, mostly from large biobanks, for external replication.
Population characteristics of all individual studies are provided in Supplementary Table 1.

Recruitment

As summarized in Supplementary Table 1 and described in greater detail in the Supplementary Appendix, participants were recruited in three different settings: (1) population-based studies (about one third of the discovery dataset); (2) clinic-based case-control studies; (3) biobanks (mostly hospital-based, about 90% of follow-up studies). In clinic/hospital-based studies patients with very severe strokes making informed consent more challenging or very minor strokes not leading to any hospitalization are less likely to be included. Stroke ascertainment in population-based studies is more comprehensive as it is conducted prospectively. Stroke subtyping is more detailed in clinic-based case-control studies than in population-based studies and biobanks.

Ethics oversight

Each contributing studies received ethical approval by the respective Institutional Review Boards (IRB). Detailed descriptions for each contributing study are given in supplementary appendix.

Note that full information on the approval of the study protocol must also be provided in the manuscript.
