# Supplementary material for: Publisher Correction: Stroke genetics informs drug discovery and risk prediction across ancestries
Source: Nature. 2022 Nov 14;612(7938):E7. doi: 10.1038/s41586-022-05492-5 (PMC9712088; doi:10.1038/s41586-022-05492-5)
Supplement: Supplementary file 1 — Supplementary Methods, including a description of the GWAS of stroke-risk factors in Tohuku Medical Megabank and Calculation of candidate polygenic score models; description of study populations in the GIGASTROKE initiative; study-specific acknowledgements; information on other members of participating consortia; and Supplementary References. [file 41586_2022_5492_MOESM1_ESM.docx]

**Stroke genetics informs drug discovery and risk prediction across ancestries**

**SUPPLEMENTARY APPENDIX**

**Table of content**

[1. Supplementary Methods 2](#_Toc113967773)

[2. Description of study populations in the GIGASTROKE initiative 4](#_Toc113967774)

[3. Study-specific acknowledgements 48](#_Toc113967775)

[4. Other members of participating consortia 69](#_Toc113967776)

[5. References 94](#_Toc113967777)

# 1. Supplementary Methods

**GWAS of stroke risk factors in Tohoku Medical Megabank**

We obtained information on lifestyle, effect of disaster, blood, and urine information from the 87,865 participants. Among the study participants, 53,599 were genotyped using a customized microarray designed by the TMM, denoted as Japonica array version 2 (JPAv2).[^1^](#_ENREF_1) Subjects compatible with the following criteria were excluded: low call rate (<0.95), non-Japanese ancestry estimated from genetic principal components (PCs), and sex-mismatch between genotype and basic resident register. Genetic PCs were computed using PLINK v 2.00a3LM.[^2^](#_ENREF_2) Variants with a low call rate (<0.95), low Hardy–Weinberg equilibrium exact test P-value (P<1×10^-6^), low minor allele frequency (MAF; <0.01) were excluded. The genotyped data were pre-phased using Eagle version 2.4.1,[^3^](#_ENREF_3) and imputed using Minimac3 version 2.0.1,[^4^](#_ENREF_4) with the 1000 Genomes Project reference panel (phase3v5; cross-ancestry).[^5^](#_ENREF_5) Individuals whose age <18 or >85 and variants with low-imputation quality (R^2^<0.3) were excluded. Then, we conducted GWAS of eight stroke risk factor traits (SBP, DBP, LDL and HDL cholesterol, triglycerides, BMI, cigarettes per day and HbA1C). For each trait, subjects with phenotypic value of outside three folds of the upper/lower quartile interquartile range were excluded from the analysis, followed by the rank-based inverse normal transformation of the phenotypic values. The GWAS sample size was up to 53,323 (SBP, 53,317; DBP, 53,323; LDL cholesterol, 48,699; HDL cholesterol, 53,318; triglycerides, 52,503; BMI, 53,256; cigarettes per day, 18,659; and HbA1C, 52,370). Association between variants and phenotypic values were estimated using BOLT-LMM version 2.3.4,[^6^](#_ENREF_6) with the adjustments for age, sex, and top 10 genetic PCs.

**Calculation of candidate polygenic score (PGS) models**

Three different methods: (P+T),[^7^](#_ENREF_7) LDpred,[^8^](#_ENREF_8) and PRScs,[^9^](#_ENREF_9), were used for calculating the candidate PGS models in Europeans and East-Asians using 1000G p3v5 European (n=503) and East-Asian (n=504) LD reference panels respectively. For all PGS calculations only variants with both MAF>1% and imputation INFO>0.8 were included in EstBB and variants with imputation R^2^>0.3 were included in BBJ data.

First, pruning and thresholding scores were built using the PLINK v1.90b6.8 (--clump) software.[^2^](#_ENREF_2) The algorithm forms clumps around SNPs with association p-values less than a provided threshold. Each clump contains all SNPs within 250kb of the index SNP that are also in LD with the index SNP as determined by a provided r2 threshold in the LD reference. PGSs were built containing the index SNPs of each clump with corresponding estimated β-coefficient (log(OR)) for its effect allele as weights. PGS models were created over a range of p-value (5e-8, 5e-6, 5e-4, 5e-2, 5e-1, or 1e-0) and r2 (0.2 0.4 0.6, or 0.8) pruning thresholds, for a total of 24 pruning and thresholding-based candidate scores for each of the GWAS summary statistics.

Second, candidate PGS models were derived using the LDpred software v1.0.11 (https://bitbucket.org/bjarni_vilhjalmsson/ldpred).[^8^](#_ENREF_8) This Bayesian approach calculates a posterior mean effect size for each variant taking into account the LD structure. The underlying Gaussian distribution additionally considers the fraction of causal markers via a tuning parameter, ρ. A range of values for the fraction of causal variants ρ was used (1.0, 0.3, 0.1, 0.03, 0.01, 0.003, or 0.001). Third, PGS models for each of the GWAS summary statistics were built using the PRScs software.[^9^](#_ENREF_9) PRScs performs Bayesian continuous shrinkage to GWAS summary statistics to account for LD. Six different global shrinkage parameters φ were considered: 1e-8, 1e−6, 1e−4, 1e−2, 1e-0 and a fully Bayesian approach that automatically learns tuning parameter φ from GWAS summary statistics

# 2. Description of study populations in the GIGASTROKE initiative

**GIGASTROKE studies previously included in MEGASTROKE**

**METASTROKE consortium**

The METASTROKE consortium has been described in detail previously.[^10^](#_ENREF_10)^,^[^11^](#_ENREF_11)

**NINDS-SIGN consortium**

The NINDS-SIGN consortium has been described in detail previously.[^10^](#_ENREF_10)^,^[^12^](#_ENREF_12)

**CHARGE consortium**

We combined data from prospective cohort studies participating in the Cohorts for Heart and Aging Research in Genomic Epidemiology (CHARGE) consortium.[^13^](#_ENREF_13)

***Age, Gene/Environment Susceptibility (AGES) -Reykjavik Study***

The AGES-Reykjavik Study is a single center prospective cohort study based on the Reykjavik Study. The Reykjavik Study was initiated in 1967 by the Icelandic Heart Association to study cardiovascular disease and risk factors.[^14^](#_ENREF_14) The cohort included men and women born between 1907 and 1935 who lived in Reykjavik at the 1967 baseline examination. Re-examination of surviving members of the cohort was initiated in 2002 as part of the AGES-Reykjavik Study. The AGES-Reykjavik Study is designed to investigate aging using a multifaceted comprehensive approach that includes detailed measures of brain function and structure. All cohort members were European Caucasians. Briefly, as part of a comprehensive examination, all participants answered a questionnaire, underwent a clinical examination and had blood drawn.Among AGES participants with GWAS data (N=3,219), after exclusion of participants with prevalent stroke (N=224), and those without follow-up for incident stroke events (N=114), N=2,996 participants were available for analyses.

*Stroke ascertainment in AGES*: Incident stroke cases were ascertained from multiple sources including hospital, general practice, nursing home records and death certificates. All possible cases were adjudicated with standard TOAST criteria by two Neurologists and a Neuroradiologist with expertise in evaluating stroke cases for epidemiologic studies.

***Atherosclerosis Risk in Communities (ARIC) Study***

The ARIC study is a prospective population-based study of atherosclerosis and clinical atherosclerotic diseases in 15,792 men and women, including 11,478 non-Hispanic white participants, drawn from 4 U.S. communities (Suburban Minneapolis, Minnesota; Washington County, Maryland; Forsyth County, North Carolina, and Jackson, Mississippi). In the first three communities, the sample reflects the demographic composition of the community. In Jackson, only black residents were enrolled. Ancestry was self-reported during an interview. Participants were handed a card and asked to tell the interviewer which best described his or her race. Choices offered were: White, Black, American Indian/Alaskan Native, Asian/Pacific Islander, Other: specify. Over 99% identified as either white or black. Only self-identified blacks were included in for COMPASS. Participants were between age 45 and 64 years at their baseline examination in 1987-1989 when blood was drawn for DNA extraction and participants consented to genetic testing. Only individuals free of stroke or TIA at baseline were included in the analysis. Single-nucleotide polymorphisms (SNPs) were genotyped on the Affymetrix 6.0 chip and were imputed to ≈2.5 million SNPs based on a panel of cosmopolitan reference haplotypes from HapMap CEU and YRI (HapMap II CEU and YRI (build 35, release 21)). MACH v1.0.16 was used to perform genotype imputations and allele dosage information was summarized in the imputation results.

*Stroke ascertainment in ARIC:* Hospitalized strokes that occurred by December 31, 2016 were included in the present study. During annual telephone contacts, trained interviewers asked each ARIC participant to list all hospitalizations during the past year. Hospital records for any hospitalizations identified were then obtained. In addition, all local hospitals annually provided lists of stroke discharges (International Classification of Diseases, Ninth Revision, Clinical Modification codes 430 to 438), which were scrutinized for ARIC participant discharges. Details on quality assurance for ascertainment and classification of stroke are described elsewhere. Briefly, the stroke diagnosis was assigned according to criteria adapted from the National Survey of Stroke. Strokes secondary to trauma, neoplasm, hematologic abnormality, infection, or vasculitis were excluded, and a focal deficit lasting <24 hours was not considered to be a stroke. Out-of-hospital stroke was not ascertained and validated; thus, these potential stroke events were not included. Strokes were classified into hemorrhagic stroke (subarachnoid and intracerebral hemorrhage) and ischemic stroke (thrombotic and embolic brain infarction). A stroke was classified as ischemic when a brain CT or MRI revealed acute infarction and showed no evidence of hemorrhage. All definite ischemic strokes were further classified as lacunar, nonlacunar thrombotic, or cardioembolic on the basis of the recorded neuroimaging results. For this analysis, the hemorrhagic strokes identified by ARIC were censored at the time of their occurrence.

***Cardiovascular Health Study (CHS) – European Ancestry***

The Cardiovascular Health Study (CHS) is a population-based cohort study of risk factors for coronary heart disease and stroke in adults ≥65 years conducted across four field centers.[^15^](#_ENREF_15) The original predominantly European ancestry cohort of 5,201 persons was recruited in 1989-1990 from random samples of people on the Medicare eligibility lists; subsequently, an additional predominantly African-American cohort of 687 persons was enrolled for a total sample of 5,888. Blood samples were drawn from all participants at their baseline examination and DNA was subsequently extracted from available samples. Because the other cohorts in the CHARGE analysis were predominantly European ancestry, the African American participants were excluded from this analysis to reduce the possibility of confounding by population structure. European ancestry participants were excluded from this GWAS study sample due to the presence at study baseline of coronary heart disease, congestive heart failure, peripheral vascular disease, valvular heart disease, stroke or transient ischemic attack, or lack of available DNA. Beyond laboratory genotyping failures, participants were excluded if they had a call rate<=95% or if their genotype was discordant with known sex or prior genotyping. After quality control, genotyping was successful for 3,268 European ancestry participants. CHS was approved by institutional review committees at each field center and individuals in the present analysis had available DNA and gave informed consent including consent to use of genetic information for the study of cardiovascular disease.

*Stroke ascertainment in CHS*: Participants were examined annually from enrollment to 1999 and continued to be under surveillance for stroke following 1999.[^16^](#_ENREF_16)^,^[^17^](#_ENREF_17) Since baseline, participants have also been contacted twice a year to identify potential cardiovascular events, including stroke. In addition, all hospitalizations were screened for potential stroke events. For suspected fatal and non-fatal events occurring with or without hospitalization, information was collected from the participant or next of kin, from medical records, and, if needed, from the participant's physician. When available, scans or reports of CT, MRI or both were reviewed centrally. Final at a consensus conference using all available information vascular neurologists adjudicated the occurrence of fatal and non-fatal stroke, stroke types, and subtypes. Stroke definitions were derived from the criteria used for the Systolic Hypertension in the Elderly Program (SHEP).[^18^](#_ENREF_18) Stroke types were ischemic, hemorrhagic and other based on brain imaging. Hemorrhagic stroke subtypes were intra-parenchymal, subarachnoid, and other. Ischemic stroke subtypes were 1) small vessel, 2) large vessel, 3) cardioembolic, and 4) other that included mostly uncertain subtypes. The approach used in CHS was developed before the TOAST criteria were published in 1993.[^19^](#_ENREF_19) Nonetheless, the two approaches are quite similar.

***Framingham Heart Study (FHS)***

The Framingham Heart Study (FHS) is a three-generation, single-site, community-based, ongoing cohort study that was initiated in 1948 to investigate prospectively the risk factors for CVD including stroke. It now comprises 3 generations of participants (N=10,333): the Original cohort followed since 1948;[^20^](#_ENREF_20) their Offspring and spouses of the Offspring, followed since 1971;[^21^](#_ENREF_21) and children from the largest Offspring families enrolled in 2000 (Gen 3).[^22^](#_ENREF_22) Gen 3 participants were not included in this analysis since they are young (mean age 40±9 years) and few have suffered strokes. The Original cohort enrolled 5209 men and women who comprised two-thirds of the adult population then residing in Framingham, MA. Survivors continue to receive biennial examinations. The Offspring cohort comprises 5124 persons (including 3514 biological offspring) who have been examined approximately once every 4 years. The population of Framingham was virtually entirely white (Europeans of English, Scots, Irish and Italian descent) in 1948 when the Original cohort was recruited. At the initial examination participants were asked for country of birth and whether or not they had any Italian ancestry. At a later examination (the 8^th^) the Offspring cohort participants were asked to identify their race from the following choices: Caucasian or white, African-American or black, Asian, Native Hawaiian or other Pacific Islander, American Indian or Alaska native or ‘prefer not to answer’. They were either asked to identify their ethnicity as either ‘Hispanic or Latino’ or not. Almost all the FHS Original and Offspring participants are white/Caucasian and none were excluded from the discovery cohort. FHS participants had DNA extracted and provided consent for genotyping in the 1990s. All available eligible participants underwent genome-wide genotyping. In 272 persons (31 with stroke), small amounts of DNA were extracted from stored whole blood and required whole genome amplification prior to genotyping. Cell lines were available for most of the remaining participants.

Among FHS participants with GWAS data (N=4,535), after exclusion of participants with prevalent stroke (N=138), and those without follow-up for incident stroke events (N=12), N=4,385 participants were available for analyses.

*Stroke ascertainment in FHS:* At each clinic exam, participants receive questionnaires, physical examinations and laboratory testing; between examinations they remain under surveillance (regardless of whether or not they live in the vicinity) via physician referrals, record linkage and annual telephone health history updates. Incident strokes have been identified since 1948 through this ongoing system of FHS clinic and local hospital surveillance and methods used have been detailed previously;[^23-25^](#_ENREF_23) they include review of medical records and collaboration with local general practitioners, emergency rooms and imaging facilities. If a participant saw a physician or was admitted to the hospital, visited an emergency room or obtained any brain imaging between biennial examinations for symptoms suggestive of TIA or stroke, a stroke neurologist from the Heart Study attempted to visit the person within 48 hours and recorded a complete history and neurological examination; this was repeated at 1, 3 and 6 months. All medical records from practitioners, hospitals, imaging centers, rehabilitation centers and nursing homes were procured for review. A panel of 3 investigators (at least 2 neurologists) adjudicated the diagnosis of stroke and determined stroke subtype in each case based on the Framingham evaluations and external records. The recruitment of Original and Offspring cohort participants at FHS had occurred long before the DNA collection with the result that the majority of stroke events in the FHS (although ascertained prospectively) were prevalent at the time of DNA collection and were excluded from these analyses. While this reduced the sample size from FHS, the meta-analyses presented here focused on incident events.

***FINRISK***

FINRISK surveys are cross-sectional, population-based studies conducted every 5 years since 1972 to monitor the risk of chronic diseases. For each survey, a representative random sample was selected from 25- to 74-year-old inhabitants of different regions in Finland. The survey included a questionnaire and a clinical examination, at which a blood sample was drawn, with linkage to national registers of cardiovascular and other health outcomes. The study protocol has been described elsewhere.^[26](#_ENREF_26)^ The current study included eligible individuals from FINRISK surveys conducted in 1992, 1997, 2002, and 2007.

*Stroke ascertainment in FINRISK:* During follow-up, participants were monitored for stroke through linkage of the study database with the National Hospital Discharge Register and the National Causes-of-Death Register. The clinical outcomes were linked to study subjects using their unique national social security ID, which is assigned to every permanent resident of Finland. The registers are countrywide covering all cardiovascular events that have led either to hospitalization or death in Finland. Their stroke diagnoses have been validated.^[27](#_ENREF_27)^ With both registers the diagnostic classification was done using the Finnish adaptation of ICD-codes: I63; not I63.6, I64 (ICD-10) / 4330A, 4331A, 4339A, 4340A, 4341A, 4349A, 436 (ICD-9) / 433, 434, 436 (ICD-8) for Ischemic stroke excluding any hemorrhagic strokes, and I60-I61,I63-I64 (not I63.6) (ICD-10) / 430, 431, 4330A, 4331A, 4339A, 4340A, 4341A, 4349A, 436 (ICD-9) / 430, 431 (except 431.01, 431.91), 433, 434, 436 (ICD-8) for all-stroke including SAH. ICD-8 codes 430, 431 (excluding codes 431.01, 431.91 of the Finnish adaptation of ICD-8*), 432, 433, 434 or with ICD-9 codes 430, 431, 433 (excluding codes 4330X, 4331X, 4339X of the Finnish adaptation of ICD-9*), 434 (excluding code 4349X of the Finnish adaptation of ICD-9*), 436, 437, 438 or with ICD-10 codes I60, I61, I63 (excluding I63.6), I64 or I69.^[28](#_ENREF_28)^ The stroke was classified as a first-ever event if there was no evidence of a previous stroke event in the patient’s history. An event found in either register was sufficient for diagnosis.

***Health, Aging, and Body Composition (Health ABC) Study***

The Health ABC study is a prospective cohort study designed to examine the associations between body composition, weight-related health conditions, and functional limitations in older adults aged 70-79 years at inception.^[29](#_ENREF_29)^ In 1997-1998, 3,075 participants were recruited from a random sample of white and all African-American Medicare eligible residents in the Pittsburgh, PA and Memphis, TN metropolitan areas. Genome-wide genotyping was performed in 1732 white participants and 1663 met all QC criteria. All participants provided informed consent and protocols were approved by the institutional review boards at both study sites.

Among Health ABC participants with GWAS data (N=1663), after exclusion of participants without follow-up for incident stroke events (N=2), N=1,661 participants were available for analyses.

*Stroke ascertainment in Health, Aging, and Body Composition (Health ABC) Study:* Participants were screened for stroke events every 6 months alternating between semi-annual phone interviews and annual clinical visits. Any self-reported hospitalization for stroke led to medical record abstraction and verification by a Health ABC Disease Adjudicator at each site. Date and causes of death were obtained from the death certificate. Causes of death were adjudicated based on the review of medical records, proxy information and autopsy report (when performed).^[30](#_ENREF_30),[31](#_ENREF_31)^ Stroke subtyping was done from medical records review.  If the medical record indicated the event was hemorrhagic or ischemic in nature, this was recorded in the Health ABC data.

***Rotterdam Study***

The Rotterdam Study is a population-based cohort study among inhabitants of a district of Rotterdam (Ommoord), The Netherlands, and aims to examine the determinants of disease and health in the elderly with a focus on neurogeriatric, cardiovascular, bone, and eye disease.^[32-34](#_ENREF_32)^ All inhabitants aged ≥55 years (N=10,275) were invited and the participation rate was 78%, yielding a total of 7983 subjects. All participants gave written informed consent to retrieve information from treating physicians. Baseline measurements were obtained from 1990 to 1993 and consisted of an interview at home and two visits to the research center for physical examination. At this baseline examination ancestry was determined by self-report. Participants were asked to identify with one of the following categories that best described their ancestry: Dutch, Caucasian, Asian, Indian, Indonesian, Mediterranean, Negroid. Less than 1% of participants chose an ancestry other than Dutch or Caucasian. Survivors have been re-examined three times: in 1993-1995, 1997-1999, and 2002-2004. All persons attending the baseline examination in 1990-93 consented to genotyping and had DNA extracted. Genome-wide genotyping was attempted in persons with high-quality extracted DNA.

In 1990-1993, 7 983 persons 55 years of age or over participated and were re-examined every 3 to 4 years. In 1999, 3 011 individuals who had become 55 years of age or moved into the study district since the start of the study were added to the cohort (Rotterdam Study-II).^[33](#_ENREF_33)^ All participants had DNA extracted at their first visit. Genotyping was attempted in participants with high-quality extracted DNA.

Among Rotterdam Study-I participants with GWAS data (N=6,291), after exclusion of participants with prevalent stroke (N=179), and those without follow-up for incident stroke events (N=46), N=6,066 participants were available for analyses.

Among Rotterdam Study-II participants with GWAS data (N=2,157), after exclusion of participants with prevalent stroke (N=76), and those without follow-up for incident stroke events (N=1), N=2,080 participants were available for analyses.

*Stroke ascertainment in Rotterdam:* All participants have been continuously monitored for major events (including stroke) through automated linkage of the study database with files from general practitioners and the municipality. In addition physician files from nursing homes and general practitioner records of participants who moved out of the Ommoord district were reviewed twice a year. For suspected stroke and TIA events, both fatal and non-fatal, additional information (including neuroimaging) was obtained from general practitioner’ and hospital records and research physicians discussed available information with an experienced stroke neurologist to verify all diagnoses and to subclassify the strokes. Strokes were subclassified into ischemic or hemorrhagic based on neuroimaging (CT or MRI within 3 weeks) mentioned in medical records. If a hemorrhage was shown the stroke was subclassified as hemorrhagic, if there were no signs of hemorrhage, the stroke was subclassified as ischemic. Furthermore, strokes were subclassified according to TOAST criteria based on the diagnostic workup mentioned in medical records.^[35](#_ENREF_35),[36](#_ENREF_36)^

***Study of Health in Pomerania (SHIP)***

The “Study of Health in Pomerania” is a population-based epidemiological study in the region of Western Pomerania, Germany.^[37](#_ENREF_37)^ In brief, from the total population of West Pomerania comprising 213 057 inhabitants in 1996, a two-stage stratified cluster sample of adults aged 20–79 years was drawn. The net sample (without migrated or deceased persons) comprised 6 265 eligible subjects, out of which 4 308 completed their baseline examinations. From July 2007 to October 2010 the ‘Life-Events and Gene-Environment Interaction in Depression’ (LEGENDE) study was carried out in the SHIP cohort. After exclusion of SHIP- 1 participants without GWAS data and a positive lifetime prevalence of stroke before the SHIP-1 examination (N=188), N=3,112 participants were available for analyses.

*Stroke ascertainment in SHIP:* SHIP participants were followed-up after a median (range) of 5.0 (4.3–8.5) years on average. New stroke events were identified based on the following sources: Self-report by participants during the follow-up visit at the clinic center, with specific questions asking for a physician diagnosis (self-reported physician's diagnosis of stroke);^[38](#_ENREF_38)^ ICD codes based on the statutory health insurance, a survey among family doctors, inpatient visits at the Greifswald University Hospital, and Death Certificates. We included cases with fatal and non-fatal strokes. For in- and outpatient data we defined any stroke as cases with a coded ICD I61, I63, I64, I69.1, I69.3, I69.4 diagnosis. For ischemic stroke we included all cases with I63.x codes based on in- and outpatient data. Data from participants with self-reported events lacking an external validation were right censored at the estimated date of event. All participants with any stroke event from any source before the baseline examination were excluded from analyses.

***Women’s Genome Health Study (WGHS)***

The WGHS (Women’s Genome Health Study) is a large cohort for genome-wide genetic analysis of a wide range of clinical phenotypes among >25 000 women, 45 years or older at baseline and with ongoing follow-up observation, now for approximately 18 years.^[39](#_ENREF_39)^ The population is derived from participants in the Women’s Health Study (WHS) who provided a blood sample at baseline. By design, participants included in the WGHS were free from dementia and stroke at baseline. Similarly, follow-up for incident stroke events was complete in the WGHS. Therefore, the total number of WGHS participants with whole genome genetic data for analysis was N=23,294.

*Stroke ascertainment in WGHS:*Since enrollment WGHS participants were followed-up annually for the occurrence of relevant clinical endpoints including stroke. The end-point ascertainment was continued in a blinded fashion through the scheduled end of the trial (March 31, 2004), when the cohort was converted to observational mode. Follow-up and validation of reported end points continues through the ongoing observational period. When a stroke endpoint was reported to occur, full medical reports were obtained and reviewed by an endpoints committee of physicians unaware of randomized treatment assignment. A confirmed stroke was defined as a new neurologic deficit of sudden onset that persisted for >24 h. Clinical information as well as computed tomographic scans or MRI were used to distinguish hemorrhagic from ischemic events.^[39](#_ENREF_39)^ Stroke subtyping definition distinguishes ischemic versus hemorrhagic events according to TOAST criteria.^[19](#_ENREF_19)^

***Multi-Ethnic Study of Atherosclerosis (MESA)***

The Multi-Ethnic Study of Atherosclerosis (MESA) is a study of the characteristics of subclinical cardiovascular disease (disease detected non-invasively before it has produced clinical signs and symptoms) and the risk factors that predict progression to clinically overt cardiovascular disease or progression of the subclinical disease.^[40](#_ENREF_40)^ MESA researchers study a diverse, population-based sample of 6,814 asymptomatic men and women aged 45-84. Thirty-eight percent of the recruited participants are white, 28 percent African-American, 22 percent Hispanic, and 12 percent Asian, predominantly of Chinese descent. Only white participants were used for the present analysis.

Participants were recruited from six field centers across the United States: Wake Forest University, Columbia University, Johns Hopkins University, University of Minnesota, Northwestern University and University of California - Los Angeles. The first examination took place over two years, from July 2000 - July 2002. It was followed by four examination periods that were 17-20 months in length. Participants have been contacted every 9 to 12 months throughout the study to assess clinical morbidity and mortality.^[40](#_ENREF_40)^

Prevalent stroke was an exclusion criterion for MESA at baseline. Among MESA white participants with GWAS data (N=2,685), we excluded those with unexpected ancestry as inferred by principal components (N=124), those with unexpected relatedness (N=35), and those without follow-up for incident stroke events (N=162), N=2,364 participants were available for analyses.

*Stroke ascertainment in MESA:* New occurrences of stroke were recorded over 7-years of follow-up. In brief, a telephone interviewer contacted each participant every 9–12 months. Information about all new cardiovascular conditions, hospital admissions, cardiovascular outpatient diagnoses, treatments, and deaths were obtained. To verify self-reported diagnoses, information was collected from death certificates and medical records for all hospitalizations and outpatient cardiovascular diagnoses, using ICD-9 and ICD-10 codes. In the case of out-of-hospital deaths, next-of-kin interviews or questionnaires were administered to physicians, relatives or friends. Two physicians from the MESA study events committee independently reviewed all medical records for end point classification and assignment of incidence dates. The reviewers were blinded to the study data. If the reviewing physicians disagreed on the event classification, they adjudicated differences. Neurologists reviewed and classified stroke as present if there was a focal neurologic deficit lasting 24 hours or until death, or if <24h, there was a clinically relevant lesion on brain imaging and no nonvascular cause. Patients with focal neurological deficits secondary to brain trauma, tumor, infections, or other non-vascular cause were excluded.^[41](#_ENREF_41)^ Ischemic strokes were distinguished from hemorrhagic stroke using findings on imaging, surgery, autopsy, or some combination of these. Ischemic stroke subtypes were assigned based on an extension of the Trial of Org 10172 in Acute Stroke Treatment (TOAST) scheme to try to reduce the number classified as undetermined.

***PROspective Study of Pravastatin in the Elderly at Risk (PROSPER)***

PROSPER was a prospective multicenter randomized placebo-controlled trial to assess whether treatment with pravastatin diminishes the risk of major vascular events in elderly. Between December 1997 and May 1999, we screened and enrolled subjects in Scotland (Glasgow), Ireland (Cork), and the Netherlands (Leiden). Men and women aged 70-82 years were recruited if they had pre-existing vascular disease or increased risk of such disease because of smoking, hypertension, or diabetes. A total number of 5804 subjects were randomly assigned to pravastatin or placebo. A large number of prospective tests were performed including Biobank tests and cognitive function measurements. A detailed description of the study has been published elsewhere.^[42](#_ENREF_42),[43](#_ENREF_43)^

Among PROSPER participants with GWAS data (N=5,244), after exclusion of participants with prevalent stroke (N=586), and those without follow-up for incident stroke events (N=0), N=4,658 participants were available for analyses.

*Stroke ascertainment in PROSPER:* Stroke was defined as any event that meets the criteria listed below:

(a) Ischemic stroke (1 of the following conditions must be met): (1) Rapid onset of focal neurologic deficit lasting >24 hours or leading to death plus evidence from neuroimaging (computed tomography or magnetic resonance imaging) showing cerebral/cerebellar infarction or no abnormality, or postmortem examination showing cerebral and/or cerebellar infarction. (2) Rapid onset of global neurologic deficit (e.g., coma) lasting >24 hours or leading to death plus evidence from neuroimaging showing infarction, or postmortem examination showing infarction. (3) Focal neurologic deficit (mode of onset uncertain) lasting >24 hours or leading to death plus evidence from neuroimaging showing infarction, or postmortem examination showing infarction.

(b) Primary intracerebral and/or cerebellar hemorrhage (1 of the following conditions must be met): (1) Rapid onset of focal neurologic deficit lasting >24 hours or leading to death, plus neuroimaging or postmortem examination showing primary intracerebral and/or cerebellar hemorrhage. (2) Rapid onset of global neurologic deficit (e.g., coma) lasting >24 hours or leading to death, plus evidence from neuroimaging or postmortem examination showing primary intracerebral and cerebellar hemorrhage. (3) Focal neurologic deficit (mode of onset uncertain) lasting >24 hours or leading to death, plus evidence from neuroimaging or postmortem examination showing primary intracerebral and/or cerebellar hemorrhage.

(c) Not known (1 of the following conditions must be met): (1) Rapid onset of focal neurologic deficit lasting >24 hours or leading to death, without neuroimaging or postmortem data available. (2) Rapid onset of global neurologic deficit (e.g., coma) lasting >24 hours or leading to death, without neuroimaging or postmortem data available. (3) Focal neurologic deficit (mode of onset uncertain) lasting >24 hours or leading to death, without neuroimaging or postmortem data available.

The PROSPER Endpoints Committee was responsible for the classification of all possible study end points. The Committee received all annual study electrocardiograms showing serial changes, information regarding domiciliary visits or hospitalizations associated with possible myocardial infarction, and information on all deaths (including postmortem reports, death certificates, hospital records, general practitioners’ records, and/or interviews of family members or witnesses).

***3C-Study***

The Three-City study is a prospective study aiming to assess the association between vascular diseases and risk of dementia. The detailed protocol of the study has been previously described.^[44](#_ENREF_44)^ The Three-City cohort is composed of non-institutionalized individuals aged 65 years and over, randomly selected from electoral rolls of three cities of France (Bordeaux, Dijon, and Montpellier), and agreeing to participate in the study. Between March 1999 and March 2001, 9,294 persons were enrolled (4,931 in Dijon, 2,104 in Bordeaux and 2,259 in Montpellier).

Up to five face-to-face examinations were performed during follow-up. Trained nurses and psychologists performed interviews and physical and cognitive measurements at the participant’s home and at the study centre. As imputation was performed separately in the 3C-Dijon sample on the one hand and the Bordeaux and Montpellier samples on the other hand, analyses were run separately in these datasets (3C-Dijon and 3C-Bordeaux-Montpellier).

In the 3C-Dijon study, among participants with GWAS data (N=4,077), after exclusion of participants with prevalent stroke (N=204), and those without follow-up for incident stroke events (N=111), N=3,762 participants were available for analyses.

In the 3C-Bordeaux-Montpellier study, after exclusion of participants without GWAS data and with prevalent stroke and those without follow-up for incident stroke events N=2,153 participants were available for analyses.

*Stroke ascertainment in 3C-Study:* At each follow-up visit, participants or informants for deceased participants were systematically questioned about the occurrence of any severe medical event or hospitalization since the last contact. For those reporting a possible stroke event, all available clinical information was collected from hospital records, and interviews with the participant’s physician, nursing home staff (for participants admitted in a nursing home during follow-up) or family. Expert panels including at least one physician specialized in vascular medicine reviewed all available clinical information and classified each event according to the International Classification of Diseases – 10^th^ Edition. Stroke was confirmed if the participant had a new focal neurological deficit of sudden onset attributable to a cerebrovascular event that persisted for more than 24 hours. Stroke was classified by the panel as ischemic stroke, intracerebral hemorrhage or of unspecified type and ischemic stroke (IS) was classified by the panel according to the TOAST classification into cardioembolic IS, large-artery IS, small vessel disease IS, IS of other etiologies, and IS of undetermined etiology.^[19](#_ENREF_19)^

**EPIC-CVD**

EPIC is a multi-centre prospective cohort study of 519,978 participants (366,521 women and 153,457 men, mostly aged 35–70 years) recruited between 1992 and 2000 in 23 centres located in 10 European countries.^[45](#_ENREF_45)^ Participants were invited mainly from population-based registers (Denmark, Germany, certain Italian centres, the Netherlands, Norway, Sweden, UK). Other sampling frameworks included: blood donors (Spain and Turin and Ragusa in Italy); screening clinic attendees (Florence in Italy and Utrecht in the Netherlands); people in health insurance programmes (France); and health conscious individuals (Oxford, UK). About 97% of the participants were of white European ancestry. Prevalent CVD was ascertained through self-reported history of MI or angina, or registry-ascertained CVD event prior to baseline. EPIC-CVD employs a nested case-cohort design,^[46](#_ENREF_46)^ analogous to the EPIC-InterAct study for type-2 diabetes which established a common set of referents through selection of a random sample of the entire cohort (“subcohort”).^[47](#_ENREF_47)^

*Stroke ascertainment in EPIC:* Centres were asked to ascertain suspected stroke cases from registries, hospital records or self-report (i.e. follow-up questionnaires). Stroke events were defined by ICD10 codes as follows: Ischemic I63, Haemorrhagic I61, SAH I60, Unclassified I64, Other CRBV I62, I65-I69, F01. Incident stroke cases have been defined as fatal and non-fatal. All centres have recorded cause-specific mortality through mortality registries and/or active follow-up, and have ascertained and validated incident fatal and non-fatal stroke through a combination of methods.

Ascertained non-fatal stroke events were validated by clinical symptoms and imaging evidence (CT/MRI) or confirmed through hospital/GP records (with assessment of notes) or confirmed through hospital records (without assessment of notes). Individuals were excluded if they had clinical symptoms but no validation was possible e.g. there was no imaging evidence, nor GP/primary care records (without assessment of notes) or registry information. Fatal stroke events were validated either by autopsy or hospital records and death certificate or by death certificate if they died in hospital. Individuals where validation was not possible were excluded. Participants with a history of stroke or MI at baseline were excluded. No further stroke subtyping was performed.

**Biobank Japan**

BioBank Japan Project (BBJ) was started in 2003 and collected DNA and clinical information from about 200,000 patients with 47 common diseases between 2003 and 2007 (BBJ 1st cohort), and about 67,000 patients with 38 diseases between 2013 and 2017 (BBJ 2nd cohort).

Eligibility of cases was determined by physicians from a collaborative network of 66 hospitals. Individuals were excluded from the analysis if they were not supposed to be East Asian ancestry, genetic sex discordance, or with other possible clinical information errors. Overlapping individuals between BBJ cohorts and in-house imputation panel were excluded before imputation. Among the 177,261 subjects genotyped from BBJ 1st cohort after QC, 17,651 cases were registered for ischemic stroke. Of all ischemic stroke cases, 1,331 were classified as large artery strokes, 758 as cardioembolic and 4,915 as small vessel stroke. Clinical information was collected by standardized questionnaire though medical records survey. Independent genotype data of 9,809 subejects from BBJ 1st cohort, and 41,929 subjects from BBJ 2nd cohort were used for the validation of polygenic risk score. 577 and 1,470 ischemic stroke cases were registered, respectively.

*Stroke ascertainment in Biobank Japan:* Stroke patients were selected from BioBank Japan. Ischemic stroke was diagnosed by physicians at collaborating hospitals and its subtypes were determined by medical record survey according to the TOAST criteria. For GWAS, individuals without any stroke or intracranial aneurysm were selected as controls ( n=159,610).

**CADISP**

The Cervical Artery Dissections and Ischemic Stroke Patients (CADISP) study was designed to identify genetic risk variants for cervical artery dissections (CeAD), a major cause of ischemic stroke in young adults.^[48](#_ENREF_48)^ As part of a secondary analysis, patients with an ischemic stroke without cervical artery dissection (non-CeAD ischemic stroke) were also recruited, in the same centers as CeAD patients. These were patients with a diagnosis of ischemic stroke, in whom CeAD had been formally ruled out according to CADISP inclusion criteria (see attachment). Non-CeAD ischemic stroke patients were frequency-matched on age (by 5-year intervals) and gender on CeAD patients. A total of 658 non-CeAD ischemic stroke patients were included in Belgium, Finland, France, Germany, Italy, and Switzerland. We excluded 19 patients due to unavailability of geographically matched healthy controls, or due to non-European origin; of the remaining 639 non-CeAD IS patients, 613 individuals had good quality DNA available and were genotyped at the CNG. Of these, a total of 555 non-CeAD IS patients aged < 60 years, who were successfully genotyped and met genotyping quality control criteria, were used for the present analysis.

The abstracted hospital records of cases were reviewed and adjudicated for IS subtype by a neurologist in each participating center. Each item required for the subtype classification was also recorded in a standardized fashion. Based on this, IS subtypes were then centrally re-adjudicated by a panel of neurologists, in agreement with the TOAST system,^[19](#_ENREF_19)^ using a more detailed subtype description from an early version of the Causative Classification System (CCS).^[49](#_ENREF_49)^

The majority of controls (N=9,046, of which 74 Finns and 8,972 non-Finnish Europeans) were selected from an anonymized control genotype database at the Centre National de Génotypage [CNG], in order to match cases for ethnic background, based on principal component analysis. European reference samples from the genotype repository at the CNG were also analyzed simultaneously to provide improved geographical resolution. Additional Finnish controls were recruited within the CADISP study, both from the general population and among spouses and unrelated friends of CADISP patients, within the Helsinki area. A total of 234 individuals were eligible for genotyping at the CNG. Of these, 213 individuals who were genotyped successfully and met quality control criteria were available for the present analysis.^[48](#_ENREF_48)^ All participants were of European ancestry.

**COMPASS**

COMPASS contains African American participants from several cohort studies. Diagnosis of stroke was adjudicated by a physician. The study has been described previously,^[50](#_ENREF_50)^ details on the individual cohorts are as follows.

***Atherosclerosis Risk in Communities (ARIC) Study***

The ARIC study is a prospective population-based study of atherosclerosis and clinical atherosclerotic diseases in 15,792 en and women, including 11,478 non-Hispanic white participants, drawn from 4 U.S. communities (Suburban Minneapolis, Minnesota; Washington County, Maryland; Forsyth County, North Carolina, and Jackson, Mississippi). In the first three communities, the sample reflects the demographic composition of the community. In Jackson, only black residents were enrolled. Ancestry was self-reported during an interview. Participants were handed a card and asked to tell the interviewer which best described his or her race. Choices offered were: White, Black, American Indian/Alaskan Native, Asian/Pacific Islander, Other: specify. Over 99% identified as either white or black. Only self-identified blacks were included in for COMPASS. Participants were between age 45 and 64 years at their baseline examination in 1987-1989 when blood was drawn for DNA extraction and participants consented to genetic testing. Only individuals free of stroke or TIA at baseline were included in the analysis. Single-nucleotide polymorphisms (SNPs) were genotyped on the Affymetrix 6.0 chip and were imputed to ≈2.5 million SNPs based on a panel of cosmopolitan reference haplotypes from HapMap CEU and YRI (HapMap II CEU and YRI (build 35, release 21)). MACH v1.0.16 was used to perform genotype imputations and allele dosage information was summarized in the imputation results.

*Stroke ascertainment in ARIC:* Hospitalized strokes that occurred by December 31, 2012 were included in the present study. During annual telephone contacts, trained interviewers asked each ARIC participant to list all hospitalizations during the past year. Hospital records for any hospitalizations identified were then obtained. In addition, all local hospitals annually provided lists of stroke discharges (International Classification of Diseases, Ninth Revision, Clinical Modification codes 430 to 438), which were scrutinized for ARIC participant discharges. Details on quality assurance for ascertainment and classification of stroke are described elsewhere. Briefly, the stroke diagnosis was assigned according to criteria adapted from the National Survey of Stroke. Strokes secondary to trauma, neoplasm, hematologic abnormality, infection, or vasculitis were excluded, and a focal deficit lasting <24 hours was not considered to be a stroke. Out-of-hospital stroke was not ascertained and validated; thus, these potential stroke events were not included. Strokes were classified into hemorrhagic stroke (subarachnoid and intracerebral hemorrhage) and ischemic stroke (thrombotic and embolic brain infarction). A stroke was classified as ischemic when a brain CT or MRI revealed acute infarction and showed no evidence of hemorrhage. All definite ischemic strokes were further classified as lacunar, nonlacunar thrombotic, or cardioembolic on the basis of the recorded neuroimaging results. For this analysis, the hemorrhagic strokes identified by ARIC were censored at the time of their occurrence.

***Cardiovascular Health Study (CHS) – African-Americans***

The Cardiovascular Health Study (CHS) is a population-based cohort study of risk factors for coronary heart disease and stroke in adults ≥65 years conducted across four field centers .^[15](#_ENREF_15)^ The original predominantly European ancestry cohort of 5,201 persons was recruited in 1989-1990 from random samples of people on the Medicare eligibility lists; subsequently, an additional predominantly African-American cohort of 687 persons were enrolled for a total sample of 5,888. Because the COMPASS consortium focused on non-European samples, only self-described African-Americans contributed to the COMPASS analyses.

Blood samples were drawn from all participants at their baseline examination and DNA was subsequently extracted from available samples. Genotyping was performed at the General Clinical Research Center’s Phenotyping/Genotyping Laboratory at Cedars-Sinai among CHS African-American participants who consented to genetic testing and had DNA available using Illumina HumanOmni1-Quad_v1 BeadChip system.

Beyond laboratory genotyping failures, participants were excluded if they had a call rate<=95% or if their genotype was discordant with known sex or prior genotyping (to identify possible sample swaps). After quality control, genotyping was successful for 823 African-American participants.

CHS was approved by institutional review committees at each field center and individuals in the present analysis had available DNA and gave informed consent including consent to use of genetic information for the study of cardiovascular disease.

***The Healthy Aging in Neighborhoods of Diversity across the Life Span Study (HANDLS) – African Americans***

In the absence of non-stroke control samples from the VISP, ISGS, and SWISS studies, controls from the Healthy Aging in Neighborhoods of Diversity across the Life Span study (HANDLS) study were used for the VISP and SWISS-ISGS case-control analyses (with no overlap across studies). Controls were sex and race/ethnicity-matched and randomly selected from all HANDLS participants not reporting history of stroke at baseline or reporting adjudicated stroke during follow-up.

HANDLS is an interdisciplinary, community-based, prospective longitudinal epidemiologic study examining the influences of race and socioeconomic status (SES) on the development of age-related health disparities among socioeconomically diverse African Americans and whites in Baltimore, MD, USA. This study assesses physical parameters over a 20-year period while evaluating genetic, biologic, demographic, and psychosocial influences. HANDLS recruited 3,722 participants (2200 African Americans (59%) and 1522 whites (41%)) from Baltimore, MD.

*Stroke Ascertainment.* Stroke status at baseline was determined through self-report while incident strokes, other vascular events, and deaths were determined using medical records and clinic visits during follow-up.

Genotyping was focused on a subset of participants self-reporting as African American and was performed at the Laboratory of Neurogenetics, National Institute on Aging, National Institutes of Health. Genotype data (for up to 907,763 SNPs) were generated for 1,024 participants using either Illumina 1M and 1M duo arrays (n=709) ,or a combination of 550K, 370K, 510S and 240S to equate the million SNP level of coverage. Inclusion criteria for genetic data in HANDLS includes concordance between self-reported sex and sex estimated from X chromosome heterogeneity, > 95% call rate per participant (across all equivalent arrays), concordance between self-reported African ancestry and ancestry confirmed by analyses of genotyped SNPs, and no cryptic relatedness to any other samples at a level of proportional sharing of genotypes > 15% (effectively excluding 1st cousins and closer relatives from the set of probands used in analyses). In addition, SNPs included in the analysis were filtered for HWE p-value > 1e-7, missing by haplotype p-values > 1e-7, minor allele frequency > 0.01, and call rate > 95%. Data analyses utilized the high-performance computational capabilities of the Biowulf Linux cluster at the NIH, Bethesda, Md. (http://biowulf.nih.gov).

***INTERSTROKE-African Americans***

INTERSTROKE is an international, multi-centered, case-control study of stroke investigating the global burden of risk factors across 32 countries and 18 different ethnic groups around the world. A detailed report of the study design has been published (Neuroepidemiology. 2010; 35:36-44). Briefly, cases were patients with acute first stroke (within 5 days of symptoms onset and 72 hours of hospital admission) in whom neuroimaging (CT or MRI) was performed. The TOAST classification system was used to define ischemic stroke subtypes. Cases were excluded if 1) they were unable to communicate due to severe stroke without a valid surrogate respondent (e.g. first-degree relative or spouse), 2) they were hospitalized for acute coronary syndrome/myocardial infarction, or 3) stroke was attributed to non-vascular causes (e.g. tumor). Controls were selected from the community and had no history of stroke.

A subset of INTERSTROKE participants consenting to genetic analysis with sufficient DNA quantities were genotyped on the Illumina Infinium Cardiometabo BeadChip. All samples were genotyped at a central site (the Genetic Molecular Epidemiology Laboratory in Hamilton, Ontario, Canada). Samples were excluded if they had 1) a high proportion of missing variants (missingness > 0.05), 2) inconsistencies between reported and genetically determined sex or ethnicity or 3) exhibited cryptic relatedness. Genotyped variants were excluded if they were rare (MAF < 0.01), exhibited high missingness across samples (missingness > 0.01), or deviated from hardy-weinberg equilibrium (P<5x10^-6^). Pre-phasing and imputation were perforrmed with SHAPEIT2 and IMPUTE, respectively, using the 1000Genomes Phase 1 Version 3 (November 23, 2010 subversion) reference panel. Imputed variants were removed if they were rare (MAF < 0.01) or of poor quality (INFO SCORE < 0.30).

***Ischemic Stroke Genetic Study (ISGS)-African Americans***

ISGS is a multicenter inception cohort study. Cases were recruited from inpatient stroke services at five United States academic medical centers. Cases are adult men and women over the age of 18 years diagnosed with first-ever ischemic stroke confirmed by a study neurologist on the basis of history, physical examination and CT or MR imaging of the brain. Cases had to be enrolled within 30 days of onset of stroke symptoms. Cases were excluded if they had: a mechanical aortic or mitral valve at the time of the index ischemic stroke, central nervous system vasculitis, or bacterial endocarditis. They were also excluded if they were known to have: cerebral autosomal dominant arteriopathy with subcortical infarcts and leukoencephalopathy (CADASIL), Fabry disease, homocystinuria, mitochondrial encephalopathy with lactic acidosis and stroke-like episodes (MELAS), or sickle cell anemia. Diagnostic evaluation included: head CT (95%) or MRI (83%), electrocardiography (92%), cervical arterial imaging (86%), and echocardiography (74%). Medical records from all cases were centrally reviewed by a vascular neurology committee and assigned ischemic stroke subtype diagnoses according to TOAST criteria, Oxfordshire Community Stroke the Baltimore-Washington Young Stroke Stud. DNA was donated to the NINDS DNA Repository (Coriell Institute, Camden, NJ) for eligible samples with appropriate written informed consent. DNA samples were genotyped using the Illumina 610 array and data analyses were supported by the high-performance computational capabilities of the Biowulf Linux cluster at the NIH (http://biowulf.nih.gov).

***Jackson Heart Study (JHS)***

The JHS is a single-site, prospective, population-based study designed to explore the environmental, behavioral, and genetic factors that influence the development of CVD among African Americans. A total of 5,301 women and men between the ages of 21 and 94 were recruited between 2000 and 2004 from a tri-county area of Mississippi: Hinds, Madison, and Rankin Counties. Participants were recruited from four sources, including (1) randomly sampled households from a commercial listing; (2) ARIC participants; (3) a structured volunteer sample that was designed to mirror the eligible population; and (4) a nested family cohort. Overviews of the JHS including the sampling and recruitment, sociocultural, and laboratory methods have been described and published previously.^[51-54](#_ENREF_51)^ The institutional review boards of the following participating institutions approved the study: the University of Mississippi Medical Center, Jackson State University, and Tougaloo College. All of the participants provided written informed consent. Unrelated participants were between 35 and 84 years old, and members of the family cohort were ≥ 21 years old when consent for genetic testing was obtained and blood was drawn for DNA extraction.

The baseline examination consisted of a home interview, self-administered questionnaires, and a clinic visit. Medications taken in the prior 2 weeks were brought to clinic and transcribed verbatim with subsequent coding by a pharmacist. After an overnight fast, anthropometric and seated blood pressure measurements were obtained and venipuncture/urine collection was performed in accordance with the National Committee for Clinical Laboratory Standards. Blood pressure was measured by trained technicians using a Hawksley random zero manometer and determined by the arithmetic average of two readings taken 1 minute apart after a five-minute rest.^[55](#_ENREF_55)^

*Stroke Assessment in the JHS:* In addition to the standard JHS examinations, participants were contacted by telephone annually beginning in 2005 to obtain interim information about cardiovascular events. (ICD-9 code 428 for hospitalizations). During the annual follow up phone call, participants or designated representative provide self-reported information of hospitalization or death. Identification and abstraction of CVD illness and death data are performed by a certified medical record abstractor. Incident stroke is defined as stroke that occurred while the participants was enrolled the study, i.e. stroke event occurred after the baseline visit. Strokes are classified as either definite or probable stroke. The definition of stroke was based on the World Health Organization (WHO) criteria for definition of stroke or clinical criteria in which case the WHO criteria might not have been satisfied, but there is clinical evidence sufficient for a diagnosis of stroke to be made. More details on identification and classification of stroke events in the JHS have already been published.^[56](#_ENREF_56),[57](#_ENREF_57)^ Although not directly relevant in this study, ischemic stroke subtyping in the JHS was done the TOAST classification criteria.

***The Sea Islands Genetics Network (SIGNET) & REasons for Geographic And Racial Differences in Stroke (REGARDS) – African Americans***

The Sea Islands Genetics Network (SIGNET) study consists of the REasons for Geographic And Racial Differences in Stroke (REGARDS), the Sea Islands Genetic African American Registry (Project SuGAR), a COBRE for Oral Health study (COBRE), and the Systemic Lupus Erythematosus in Gullah Health study (SLEIGH). All subjects are African Americans (AA), and all provided written informed consent.

All SIGNET samples (n= 4,298) were genotyped using the Affymetrix Genome-Wide Human SNP Array 6.0. Imputation was performed using MACH (version 1.0.16) to impute all autosomal SNPs using the CEU+YRI reference panel (as supplied by Goncalo Abecasis) from build 36 (2,318,207 SNPs in total).

REGARDS is an observational cohort of 30,239 AA and white men and women enrolled in their homes after a telephone interview in 2003-7 (Howard VJ et al., 2005). Participants were a national sample oversampled from the southeastern stroke belt (56%) and were 58% female and 42% black by design. Participants were followed every 6 months by telephone to ascertain health outcomes, with validation of stroke, coronary heart disease, death and other ancillary study endpoints. For SIGNET, we selected all AA REGARDS type 2 diabetes (T2D) cases recruited from SC, GA, NC, and AL, and an equivalent number of race, sex, and age-strata matched diabetes-free controls. We also included all participants not already included that were current residents of the 15-county “Low Country” region of SC and GA (SC counties Beaufort, Berkeley, Charleston, Colleton, Dorchester, Georgetown, Hampton, Horry, Jasper; GA counties Bryan, Camden, Chatham, Glynn, Liberty, McIntosh). The subset of REGARDS participants genotyped under SIGNET are referred to as SIGNET-REGARDS. GWAS genotyping was completed among 2398 SIGNET-REGARDS AA participants, including 1149 with diabetes and 1249 without diabetes.

***Siblings with Ischemic Stroke Study (SWISS) – African Americans***

SWISS is a prospective multicenter affected sibling pair study of first-ever or recurrent ischemic stroke. Subjects were recruited from 54 enrolling hospitals across the US and Canada. Samples were collected between 1999-2011. Ischemic stroke probands were enrolled at 66 US medical centers and 4 Canadian medical centers. All recruits were extensively clinically phenotyped and have imaging- confirmed ischemic stroke using either CT or MRI brain scans. Probands are adult men and women over the age of 18 years diagnosed with ischemic stroke confirmed by a study neurologist on the basis of history, physical examination and CT or MR imaging of the brain who also have a history of at least one living sibling with a history of stroke. Probands were excluded if 1) they had a mechanical aortic or mitral valve at the time of the index ischemic stroke, central nervous system vasculitis, or bacterial endocarditis or 2) were known to have cerebral autosomal dominant arteriopathy with subcortical infarcts and leukoencephalopathy (CADASIL), Fabry disease, homocystinuria, mitochondrial encephalopathy with lactic acidosis and stroke-like episodes (MELAS), or sickle cell anemia. Siblings were enrolled using proband- initiated direct contact when permitted by Institutional Review Boards. Concordant siblings had their diagnosis of ischemic stroke confirmed by review of medical records by a central vascular neurology committee. Concordant siblings had the same eligibility criteria as probands. Subtype diagnoses were assigned to the index strokes of probands and concordant siblings according to TOAST criteria. Discordant siblings of the proband were confirmed to be stroke-free using the Questionnaire for Verifying Stroke-free Status. DNA samples were genotyped using the Illumina 660 array and data analyses were supported by the high-performance computational capabilities of the Biowulf Linux cluster at the NIH (http://biowulf.nih.gov).

***Women’s Health Initiative (WHI) – African Americans***

The goal of the WHI was to investigate the etiology and prevention of chronic disease in post-menopausal women . Approximately 161,000 postmenopausal women 50–79 years of age from 40 clinical centers in the US were recruited between 1993 and 1998. WHI consists of an observational study (OS), and clinical trials (CT) of postmenopausal hormone therapy (estrogen alone or estrogen plus progestin), a calcium and vitamin D supplement trial, and a dietary modification trial. A subset of 8,515 African American women who provided consent for DNA analysis were randomly selected for genome-wide genotyping as part of the SNP Health Association Resource (SHARe). .

Genetic data were obtained from genome-wide scans using the Genome-wide Human SNP Array 6.0 (Affymetrix, Santa Clara, CA, www.affymetrix.com) of 909,622 single nucleotide polymorphisms (SNPs). Genotyping quality control included examination of concordance rates for blinded and un-blinded duplicates. Approximately 1% of SNPs failed genotyping and SNPs with call rates < 95% or concordance rates <98%, or minor allele frequency <1% were excluded. In addition to the genotyping, SNPs were imputed using 1000 Genomes Project phase 1 integrated variant set (Aug 2012). Principal components were calculated for each individual and evaluated for their contribution to ancestral variation. Because most of the ancestral variation was explained by the first 4 PCs, only these were included as covariates in the analyses.

Stroke ascertainment in WHI: All incident strokes, other vascular events, and deaths were identified through self-report at annual (OS) and semi-annual (CT) participant contacts, and through third- party reports by family members and proxies. Medical records were obtained for potential strokes, and adjudication was performed by trained physician adjudicators who assigned a diagnosis. Stroke diagnosis requiring and/or occurring during hospitalization was based on rapid onset of a neurological deficit attributable to an obstruction or rupture of an arterial vessel system. The deficit was not known to be secondary to brain trauma, tumor, infection or other cause and must have lasted more than 24 hours unless death supervened or a lesion compatible with acute stroke was evident on computed tomography or magnetic resonance imaging were classified as ischemic, hemorrhagic or unknown/missing. Ischemic stroke subtypes were further classified using Trial of Org 10172 in Acute Stroke Treatment (TOAST) analyses, strokes subtypes judged as ‘probable’ or ‘possible’ were combined. African American women passing the above genotyping quality control criteria, with follow-up data, and without a history of stroke at baseline were included in the WHI analyses. All participants provided written, informed consent.

***SiGN***

The Stroke Genetics Network (SiGN) study was funded by a cooperative agreement grant from the National Institute of Neurological Disorders and Stroke (NINDS) U01 NS069208. Genotyping services were provided by the Johns Hopkins University Center for Inherited Disease Research (CIDR), which is fully funded through a federal contract from the National Institutes of Health (NIH) to the Johns Hopkins University (contract No.HHSN268200782096C). The Biostatistics Department Genetics Coordinating Center at the University of Washington (Seattle) provided more extensive quality control of the genotype data through a subcontract with CIDR. Additional support to the Administrative Core of SiGN was provided by the Dean’s Office, University of Maryland School of Medicine. SiGN- Group 4 consists of AA subjects from the GASROS, GCNKSS, ISGS, MCISS, MIAMISR, NOMAS, REGARDS, SPS3, SWISS, WHI, and WUSTL studies. MGH-GASROS: The Massachusetts General Hospital Stroke Genetics Group was supported by the NIH Genes Affecting Stroke Risks and Outcomes Study (GASROS) grant K23 NS042720, the American Heart Association/Bugher Foundation Centers for Stroke Prevention Research 0775010N, and NINDS K23NS042695, K23 NS064052, the Deane Institute for Integrative Research in Atrial Fibrillation and Stroke, and by the Keane Stroke Genetics Fund. Genotyping services were provided by the Broad Institute Center for Genotyping and Analysis, supported by grant U54 RR020278 from the National Center for Research Resources. GCNKSS: The Greater Cincinnati/Northern Kentucky Stroke Study (GCNKSS) was supported by the NIH (NS030678). MCISS: The Middlesex County Ischemic Stroke Study (MCISS) was supported by intramural funding from the New Jersey Neuroscience Institute/JFK Medical Center, Edison, NJ, and The Neurogenetics Foundation, Cranbury, NJ. We acknowledge Dr Souvik Sen for his advice and encouragement in the initiation and design of this study. MIAMISR and NOMAS: The Northern Manhattan Study (NOMAS) was supported by grants from the NINDS (R37 NS029993, R01 NS27517). The Cerebrovascular Biorepository at University of Miami/Jackson Memorial Hospital (The Miami Stroke Registry, Institutional Review Board No. 20070386) was supported by the Department of Neurology at University of Miami Miller School of Medicine and Evelyn McKnight Brain Institute. Biorepository and DNA extraction services were provided by the Hussmann Institute for Human Genomics at the Miller School of Medicine. SPS3: The Secondary Prevention of Small Subcortical Strokes trial was funded by the US National Institute of Health and Neurological Disorders and Stroke grant No. U01NS38529- 04A1 (principal investigator, Oscar R. Benavente; coprincipal investigator, Robert G. Hart). The SPS3 Genetic Substudy (SPS3-GENES) was funded by R01 NS073346 (coprincipal investigators, Julie A. Johnson, Oscar R. Benavente, and Alan R. Shuldiner) and U01 GM074492-05S109 (principal investigator, Julie A. Johnson). WUSTL: Washington University St. Louis Stroke Study (WUSTL): The collection, extraction of DNA from blood, and storage of specimens were supported by 2 NINDS NIH grants (P50 NS055977 and R01 NS8541901). Basic demographic and clinical characterization of stroke phenotype was prospectively collected in the Cognitive Rehabilitation and Recovery Group (CRRG) registry. The Recovery Genomics after Ischemic Stroke (ReGenesIS) study was supported by a grant from the Barnes-Jewish Hospital Foundation.

**deCODE study**

Icelandic ischemic stroke cases (5,520), were identified from a registry of individuals diagnosed with ischemic stroke or TIA at Landspitali University Hospital in Reykjavik, the only tertiary referral centre in Iceland, during the years 1993 to 2013. The ischemic stroke or TIA diagnoses were based on standard WHO criteria and imaging evidence (either CT or MRI), and were clinically confirmed by neurologists. Eligible patients who survived the stroke were invited to participate the genetic study, either by attending a recruitment centre for deCODE’s genetic studies, or they were visited at their home by a study nurse. Patients were classified into causative subtypes according to the Trial of Org 10172 in Acute Stroke Treatment (TOAST).

The study is based on whole-genome sequencing of 8,453 Icelanders and Illumina SNP chip genotyping of 151,677 Icelanders.  Genotypes for the chip-typed individuals are phased using the method of long-rage phasing (Kong et al. Detection of sharing by descent, long-range phasing and haplotype imputation Nat Genet 40, 1068-75, 2008). and genotype probabilities for un-typed variants are imputed into the chip-typed individuals, and their close relatives, using phased genotypes for the 8.453 WGS individuals as reference. Association testing for case–control analysis was performed using logistic regression, adjusting for age and county and assuming a multiplicative model of risk. About 25 million variants, all with imputation info over 0.8, were tested for association. To account for inflation in the test statistics due to cryptic relatedness and stratification within the case and control sample sets, we applied the method of LD score regression (Bulik-Sullivan B. K. et al. LD Score regression distinguishes confounding from polygenicity in genome-wide association studies. Nat. Genet. 47, 291–295, 2015).

Control comprised 254,176 individuals recruited through different genetic projects at at deCODE. Individuals with confirmed stroke (identified by cross-matching with hospital lists) were excluded as controls.

The study was approved by the Data Protection Commission of Iceland and the National Bioethics Committee of Iceland. All participants gave informed consent.

**Glasgow Stroke Sample**

Cases with ischemic stroke attending the cerebrovascular service of the Western Infirmary, Glasgow, were recruited between 1990 and 2004 as part of an ongoing study of genetic and circulating biomarkers in stroke. All patients underwent brain imaging and extracranial carotid ultrasound in accordance with a standard clinical protocol. The study was approved by the West Ethics Committee.

Controls were drawn from shared WTCCC controls obtained from the 1958 Birth Cohort. This is a prospectively collected cohort of individuals born in 1958, and ascertained as part of the national child development study.^[58](#_ENREF_58)^ Data from this cohort are available as a common control set for a number of genetic and epidemiological studies.

**Hisayama and HISYAMA-FSR study**

A detailed study description was described previously^[59](#_ENREF_59)^. The individuals with ischemic stroke were recruited from seven hospitals in Fukuoka Prefecture, Japan (Kyushu University Hospital, Hakujyuji Hospital, Fukuoka Red Cross Hospital, Kyushu Medical Center, Imazu Red Cross Hospital, Fukuoka Higashi Medical Center, and Seiai Rehabilitation Hospital) in 2004 (The Fukuoka Stroke Registry [FSR]) . The Hisayama Study is an ongoing population-based epidemiological study of cardiovascular disease in the town of Hisayama, Fukuoka Prefecture, Japan. A total of 3,328 individuals aged 40 years or older participated in the screening survey and underwent a comprehensive assessment in 2002-2003.

*Stroke ascertainment in the Hisayama and Hisayama-FSR study:* Ischemic stroke was defined as a sudden non-convulsive, focal neurologic deficit lasting longer than 24 hours due to brain ischemia. The diagnoses of ischemic stroke and its subtypes for all cases were made by stroke neurologists of the hospitals, referring to detailed clinical features and ancillary laboratory examinations: namely, cerebral angiography, brain imaging (including computed tomography and magnetic resonance imaging), echocardiography, and carotid duplex imaging. Subtypes of ischemic stroke were determined on the basis of the Classification of Cerebrovascular Disease III proposed by the National Institute of Neurological Disorders and Stroke and the TOAST classification.

**Heart and Vascular Health Study (HVH 1 & 2)**

The setting for this study was Group Health (GH), a large integrated health care system in western Washington State (Kaiser Permanente Washington, as of February 2017).

Data were utilized from an ongoing case-control study of incident myocardial infarction (MI) and stroke cases with a shared common control group. Methods for the study have been described previously and are briefly summarized below.^[60-62](#_ENREF_60)^ The study was approved by the human subjects committee at GH, and written informed consent was provided by all study participants.

All study participants were GH members and aged 30-79 years. MI and stroke cases were identified from hospital discharge diagnosis codes and were validated by medical record review. Controls were a random sample of GH members frequency matched to MI cases on age (within decade), sex, treated hypertension, and calendar year of identification. The index date for controls was a computer-generated random date within the calendar year for which they had been selected. For stroke cases, the index date was the date of admission for the first acute stroke. Participants were excluded if they were recent enrollees at GH, had a history of prior stroke, or if the incident event was a complication of a procedure or surgery.

Trained medical record abstractors collected eligibility and risk factor information from a review of the GH medical record using only data available prior to the index date and through a telephone interview. Medication use was ascertained using computerized GH pharmacy records. A venous blood sample was collected from all consenting subjects, and DNA was extracted from white blood cells using standard procedures.

Diagnostic criteria for ischemic stroke were adopted from the Cardiovascular Health Study.^[17](#_ENREF_17)^ These criteria included (1) rapid onset of neurologic deficit or subarachnoid hemorrhage, (2) deficit persisting for longer than 24 hours unless computed tomography or magnetic resonance imaging show evidence of permanent damage, and (3) no underlying brain trauma, tumor, or infection to cause symptoms.

Ischemic stroke cases satisfied one or more of the following criteria: (a) Focal deficit, without evidence of blood on CT or MRI, (b) Focal deficit, with mottled appearance in the appropriate location on CT, or (c) surgery or autopsy evidence of infarction.

Among ischemic strokes, the subtypes were defined as follows:

Small artery IS required either: (a) CT/MRI demonstrates a deep area of infarction (decreased density) less than 2 cm. across, or (b) A normal CT, but the clinical syndrome is typical of a lacunar infarction, that is: a pure motor stroke, a pure sensory stroke, hemiparesis plus ataxia, or dysarthria plus a clumsy hand. Cardioembolic IS required either (a) a recognized source of emboli such as atrial fibrillation, endocarditis, mitral stenosis, thrombus in heart, recent MI or cardiac surgery, or (b) a mottled appearance consistent with infarction on the CT. Large artery IS was defined by the absence of apparent source of emboli or evidence of lacunar infarction and evidence of large vessel atherosclerosis by carotid ultrasound or angiography.

All participants were of European ancestry.

**INTERSTROKE** (European, Asian, and Latin-american)

INTERSTROKE is an international, multi-centered, case-control study of stroke investigating the global burden of risk factors across various regions and ethnic groups around the world. A detailed report of the study design has been published.^[63](#_ENREF_63)^ Briefly, cases were stroke patients with acute first stroke (within 5 days of symptoms onset and 72 hours of hospital admission) in whom neuroimaging (CT or MRI) was performed. Stroke was defined with the WHO clinical criteria for stroke. The TOAST classification system was used to define ischemic stroke subtypes. Cases were excluded if 1) they were unable to communicate due to severe stroke without a valid surrogate respondent (e.g. first-degree relative or spouse), 2) they were hospitalized for acute coronary syndrome/myocardial infarction, or 3) stroke was attributed to non-vascular causes (e.g. tumor). Controls were selected from the community and had no history of stroke. The study was approved by the ethics committees in all participating centres. All participants, or their proxy, provided written informed consent before taking part in the study.

**MDC**

MDC is a prospective, population-based cohort study that included 28 449 randomly selected men (born between 1923 and 1945) and women (born between 1923 and 1950) at baseline examinations between 1991 and 1996.^[64](#_ENREF_64)^

Controls were drawn from the same cohort matched for gender, age, and time of baseline investigation. All participants provided written informed consent, and the study was approved by the ethical committee at Lund University, Lund, Sweden.

*Stroke ascertainment in MDC:* Subjects with ischemic stroke after the baseline examination were identified in the Stroke Register of Malmö until December 31, 2005. A specialized nurse from the stroke register systematically searched for and registered patients with stroke who lived in the city of Malmö. The research nurse, with a senior physician, validated the diagnosis by reviewing medical records. Criteria for stroke was rapidly developing clinical signs of local or global loss of cerebral function lasting for >24 hours or leading to death before then, with no apparent cause other than cerebral ischemia or hemorrhage. Stroke was classified as subarachnoid hemorrhage (International Classification of Diseases, Ninth Revision [ICD-9] code 430), intracerebral hemorrhage (ICD 431), cerebral infarction (ICD 434), and undetermined stroke. Subjects with stroke before the baseline examination were excluded.

**Risk Assessment of Cerebrovascular Events (RACE) Study**

The Risk Assessment of Cerebrovascular Events (RACE) Study, Pakistan is a retrospective case-control study designed to identify and evaluate genetic, lifestyle and biomarker determinants of stroke and its subtype in Pakistan. Samples were recruited from six hospital centres in Pakistan. Cases were eligible for inclusion in the study if they: (1) are aged at least 18 years; (2) presented with a sudden onset of neurological deficit affecting a vascular territory with sustained deficit at 24 hours verified by medical attention within 72 hours after onset (onset is defined by when the patient was last seen normal and not when found with deficit); (3) the diagnosis was supported by CT/MRI; and (4) presented with a Modified Rankin Score of < 2 prior to the stroke. TOAST and Oxfordshire classification systems were used to sub-phenotype all stroke cases. Control participants were individuals enrolled in the Pakistan Risk of Myocardial Infarction Study (PROMIS), a case/control study of acute MI based in Pakistan.^[65](#_ENREF_65)^ Controls in PROMIS were recruited following procedures and inclusion criteria as adopted for RACE cases. In order to minimize any potential selection biases, PROMIS controls selected for this stroke study were frequency matched to RACE cases based on age and gender and were recruited in the following order of priority: (1) non-blood related or blood related visitors of patients of the out-patient department; (2) non-blood related visitors of stroke patients; (3) patients of the out-patient department presenting with minor complaints.

**The Sahlgrenska Academy Study on Ischemic Stroke (SAHLSIS)**

SAHLSIS is a case-control study of ischemic stroke in western Sweden, which has been described in detail elsewhere.^[66](#_ENREF_66)^ Briefly, adult subjects who presented with first-ever or recurrent acute ischemic stroke before 70 years of age were recruited consecutively at stroke units. Community controls were randomly selected from a population-based health survey or from the Swedish Population Register to match the cases with regards to age (+/- 1 year), sex and geographical residence area. Control individuals with a history or sign of stroke, coronary heart disease or peripheral artery disease were excluded as described.^[66](#_ENREF_66)^ All participants were of European origin. Patients were not excluded based on stroke severity or whether they were enrolled in a treatment trial. All patients underwent ECG and neuroimaging at the acute stage (all by CT and 58% also by MRI). Additional diagnostic work-up was performed when clinically indicated. Inclusion criteria was ischemic stroke which was defined as an episode of focal neurological deficits with acute onset and lasting > 24 hours or until death, with no apparent non-vascular cause, and no signs of primary hemorrhage on brain imaging. Subjects were excluded if they had a diagnosis of cancer at advanced stage, infectious hepatitis or human immunodeficiency virus. Ischemic stroke subtype was assigned according to modified TOAST criteria.^[66](#_ENREF_66)^ A subgroup of SAHLSIS is also part of the SiGN study.

**SIFAP**

The SIFAP study is a multicenter study carried out to determine the frequency of Fabry disease in an unselected group of young adult patients with acute cerebrovascular events defined as having had an acute ischemic stroke or transient ischemic attack less than three months before enrollment into the study. The study is briefly summarized here. First-ever (80.5%) and recurrent ischemic strokes were included. MRI was a mandatory procedure but, in the case of negative or missing MRI, a qualified stroke neurologist could confirm the clinical diagnosis. For this project, ischemic stroke cases recruited from 15 sites throughout Germany and determined not to have Fabry Disease were included in the analysis. All were of European ancestry and had age of first stroke of 18 – 55 years. The diagnosis of Fabry disease was based in males as well as in females in the first level on the sequencing data of the entire exon structure including promoter of the α-galactosidase gene. In cases where a mutation was detected, biochemical analysis was done. Stroke cases from SIFAP were genotyped at CIDR (Baltimore, MD) using the Illumina Human Omni 2.5MQuad array. Only those cases without Fabry disease were selected for genotyping. Controls free of cardiovascular diseases were selected from the KORA Study previously genotyped at CIDR in the same platform. The Cooperative Health Research in the Region of Augsburg (KORA) study is a population-based study of cardiovascular and metabolic traits carried out in the region of Augsburg, Southern Germany. A subset of control subjects (N = 28) was re-genotyped together with cases to provide cross-set duplicates. This joint clustering was used to minimize possible artifactual differences in allelic frequency between cases and controls due to genotyping at different times, and the cross-set duplicates were used to detect such artifacts that may have occurred.

**UK - young lacunar stroke DNA resource**

A total of 1,029 Caucasian patients with lacunar stroke, aged ≤70 years, were recruited from 72 specialist’s stroke centres throughout the UK between 2002 and 2012, as part of the Young Lacunar Stroke DNA Resource. DNA samples were available in 930 patients. An additional 82 Caucasian patients of all ages with lacunar stroke were recruited from St. George’s Hospital, London as part of the GENESIS study.^[67](#_ENREF_67)^ Lacunar stroke was defined as a clinical lacunar syndrome, with an anatomically compatible lesion on MRI (subcortical infarct ≤15 mm in diameter). All patients underwent full stroke investigation including brain MRI, imaging of the carotid arteries and ECG. Echocardiography was performed when appropriate. All MRIs and clinical histories were reviewed centrally by one physician. Exclusion criteria were: stenosis >50% in the extra- or intracranial cerebral vessels, or previous carotid endarterectomy; cardioembolic source of stroke, defined according to the TOAST (Trial of Org 10172 in Acute Stroke Treatment) criteria as high or moderate probability; cortical infarct on MRI; subcortical infarct > 15mm in diameter, as these can be caused by embolic mechanisms (striatocapsular infarcts); any other specific cause of stroke (e.g. lupus anticoagulant, cerebral vasculitis, dissection, monogenic cause of stroke). All cases were screened for *NOTCH3* CADASIL and Fabry disease mutations and positive cases excluded.

Unrelated Caucasian controls, free of clinical cerebrovascular disease, were obtained by random sampling, stratified for age and sex, from general practice lists from the same geographical location as the patients. All patients and controls underwent a standardized clinical assessment and completed a standardized study questionnaire. MRI was not performed in controls.

The study was approved by the Multi-Centre Research Ethics Committee (04/MRE00/36) and informed consent was obtained from all participants.

**ICH**

Case and control subjects included in the discovery phase were subjects of European ancestry aged >55 years in the Genetics of Cerebral Hemorrhage with Anticoagulation13 (GOCHA) study (multicenter study in the US) and aged >18 years in the Genetic and Environmental Risk Factors for Hemorrhagic Stroke (GERFHS) studies I and II in Cincinnati, OH; Hospital del Mar Intracerebral Hemorrhage study and Vall d’Hebron Hospita lCH study in Barcelona, Spain; Jagiellonian University Hemorrhagic Stroke Study in Krakow, Poland; and the Lund Stroke Register study in Lund, Sweden. Because of their limited sample sizes, data from the four European studies (ESs) were analyzed together for the purposes of quality control, imputation, and association testing.

Cases were ascertained across participating studies according to predefined standardized criteria. Spontaneous ICH was defined as a new and acute neurological deficit with compatible brain imaging (computed tomography or magnetic resonance imaging) showing the presence of intraparenchymal bleeding. According to standard research and clinical practice in the field, ICH location was assigned based on admission images by neurologists who were blinded to genotype data. ICH originating at the cerebral cortex or cortical-subcortical junction (with or without involvement of subcortical white matter) was defined as lobar, and ICH originating at the thalamus, internal capsule, basal ganglia, deep periventricular white matter, cerebellum, or brain stem was defined as nonlobar. Exclusion criteria included trauma, brain tumor, hemorrhagic transformation of ischemic stroke, vascular malformation, and any other cause of secondary ICH.

Control subjects were ICH-free individuals enrolled from the same population that gave rise to the case subjects at each participating study site, aged >55 years (GOCHA) and >18 years (GERFHS and ESs). Control subjects were sampled by random digit dialing in GERFHS and from ambulatory clinics in the remainder of the studies.

All studies were approved by the Institutional Review Board or ethics committee at each participating site. Participants provided informed consent; when subjects were not able to communicate, consent was obtained from their legal proxies.

**GIGASTROKE new studies**

**Epidemiological Prevention Study of Zoetermeer (EPOZ)**

From 1975 to 1978 a population survey was undertaken in Zoetermeer, a Dutch town of 60,000 inhabitants at that time in the western part of the Netherlands. The objective was to assess the prevalence and determinants of cardiovascular and other chronic diseases. All 13,462 inhabitants aged 5 years or over of two districts were invited to participate. At entry into the study the participants were interviewed by a physician, a venous blood sample and a urine sample was obtained, a self-administered questionnaire was checked, and a brief physical examination, including measurements of blood pressure pulse rate and body mass index, was performed.^[68](#_ENREF_68)^

**FinnGen study**

The FinnGen study is a nationwide genetic study comprising genotyped samples from hospital biobanks and prospective epidemiological cohorts. The current study included data from sping 2020 (data freeze 5), with 171,548 and 12,681 genotyped individuals from hospital biobanks and prospective epidemiological surveys (excluding FINRISK), respectively.^[69](#_ENREF_69)^

During follow-up, participants were monitored for stroke through linkage of the study database with the National Hospital Discharge Register and the National Causesof-Death Register. The clinical outcomes were linked to study subjects using their unique national social security ID, which is assigned to every permanent resident of Finland. The registers are nationwide covering all cardiovascular events that have led either to hospitalization or death in Finland. Their stroke diagnoses have been validated.^[70](#_ENREF_70)^

With both registers the diagnostic classification was done using the Finnish adaptation of ICD-codes: I63; not I63.6, I64 (ICD-10) / 4330A, 4331A, 4339A, 4340A, 4341A, 4349A, 436 (ICD-9) / 433, 434, 436 (ICD-8) for Ischemic stroke excluding any hemorrhagic strokes, and I60-I61,I63-I64 (not I63.6) (ICD-10) / 430, 431, 4330A, 4331A, 4339A, 4340A, 4341A, 4349A, 436 (ICD-9) / 430, 431 (except 431.01, 431.91), 433, 434, 436 (ICD-8) for allstroke including SAH. ICD-8 codes 430, 431 (excluding codes 431.01, 431.91 of the Finnish adaptation of ICD-8*), 432, 433, 434 or with ICD-9 codes 430, 431, 433 (excluding codes 4330X, 4331X, 4339X of the Finnish adaptation of ICD-9*), 434 (excluding code 4349X of the Finnish adaptation of ICD-9*), 436, 437, 438 or with ICD-10 codes I60, I61, I63 (excluding I63.6), I64 or I69.34 The stroke was classified as a first-ever event if there was no evidence of a previous stroke event in the patient’s history. An event found in either register was sufficient for diagnosis.

The Coordinating Ethical Committee of the Hospital District of Helsinki and Uusimaa approved both FinnGen study protocols. All participants gave informed written consent.

**The Trøndelag Health Study (HUNT)**

The Trøndelag Health Study (HUNT) is an ongoing population-based cohort study from the county of Nord-Trøndelag in Norway.^[71](#_ENREF_71)^ All inhabitants aged 20 years or older were invited to participate in the HUNT1 survey (1984-1986), the HUNT2 survey (1995-1997), and the HUNT3 survey (2006-2008). All participants have provided questionnaire, interview, and measurement data, which can be found at the HUNT databank [https://hunt-db.medisin.ntnu.no/hunt-db]. In addition, about 80,000 participants have provided biological samples for storage at the HUNT biobank [https://www.ntnu.edu/hunt/hunt-biobank]. The Norwegian Identification Number was used to link data from the HUNT database and the HUNT biobank to other registries.

The health care system in Norway is publicly funded and the neurological departments in Nord-Trøndelag have catchment area responsibilities for the whole county. We obtained data from local hospital registries on ICD-9 and ICD-10 hospital discharge codes from all inpatient and outpatient contacts from 1987 through 2018 for all genotyped participants in the HUNT study.^[72](#_ENREF_72)^ Also, we obtained data on all who were registered in the national quality register Norwegian Stroke Register between its establishment in 2012 and 2016.^[73](#_ENREF_73)^

To define incident stroke we excluded cases who at baseline were registered with a hospital discharge diagnosis of stroke (ICD-10 I60-I69 or ICD-9 430-438); who self-reported having had a stroke (in HUNT questionnaires); or who had a stroke diagnosis in the Norwegian Stroke Register. Controls were defined as those who were born before 1948 (meaning that they were ≥ 70 years of age at the end of follow-up); were not registered with a hospital discharge diagnosis of stroke (ICD-10 I60-I69 or ICD-9 430-438); had no self-reported stroke in the HUNT questionnaires; and were not registered with a stroke diagnosis in the Norwegian Stroke Register.

The study was approved by the Regional Committee for Medical and Health Research Ethics (ref. 2015/578).

**UKBIOBANK**

For definition of stroke cases, we used UKB fields 42007 and 42009, the algorithmically defined stroke outcome, including only incident strokes (first stroke diagnosed during follow‐up; http://biobank.ctsu.ox.ac.uk/crystal/docs/alg_outcome_stroke.pdf). Stroke events that were self‐reported only without corroborating evidence from medical records were excluded due to substantial uncertainty about the accuracy of stroke self‐report. Coded hospital admissions and death record data (International Classification of Diseases, 9th and 10th revision coding systems) were included based on previous work showing good accuracy of these data sources for identifying stroke cases. Participants without a stroke diagnosis were included as controls. Related participants and those of non–white‐British descent were excluded, as were single nucleotide polymorphisms (SNPs) with minor allele frequency < 0.01. The imputed data was analyzed using logistic regression with 10 ancestry principal components, age, sex and genotyping array included as covariates using PLINK2. All participants provided written informed consent; the UKB received ethical approval from the National Research Ethics Service Committee North West-Haydock (reference 11/NW/0382), and all study procedures were in accordance with the World Medical Association for medical research. Access to the UK Biobank data was obtained under application number 2532.

**Estonian Biobank (EstBB)**

Estonian Biobank (EstBB) is an Estonian population-based cohort that consists of ~200,000 adults. All biobank participants have signed a broad informed consent form and analyses were carried out under ethical approval 1.1-12/624 from the Estonian Committee on Bioethics and Human Research and data release N05 from the EstBB.^[74](#_ENREF_74)^ Stroke case/control information was collected from the Estonian Health Insurance Fund (HIF) and E-Health Foundation data, Tartu University Hospital (TUH) and North Estonia Medical Centre data (PERH), death registry, and from questionnaires on recruitment to EstBB. Only unrelated individuals were included in the analysis. Individuals with stroke were identified using ICD-10 codes I60-I64, incident stroke cases were defined as individuals first diagnosed with stroke only after inclusion in the EstBB and prevalent stroke cases were defined as individuals first diagnosed with stroke before joining the EstBB. For the stroke subtype analysis, cases of intracerebral hemorrhage, any ischemic stroke, cardioembolic ischemic stroke, small vessel ischemic stroke and large artery ischemic stroke were defined using ICD-10 codes I61, I63, I63.4, I63.5 and I63.0 respectively, and all individuals with any other stroke diagnosis were excluded.

**Estonian Young Stroke Registry (UTARTU-EstBB)**

Estonian Young Stroke registry is a prospective ongoing hospital-based registry of all consecutive patients aged 18-54 years hospitalised to Tartu University Hospital, the second largest hospital in Estonia, with discharge diagnosis of acute ischemic stroke since January 1^st^ 2013. From 2013 to 2015 also patients hospitalised to North Estonia Medical Centre, the largest hospital in Estonia in Tallinn, were included in the registry. Ischemic stroke was defined as a focal neurological deficit of acute onset lasting more than 24h or with evidence of acute brain ischaemia on neuroimaging, when symptoms lasted <24 h.

All patients were diagnosed and managed by stroke neurologists and evaluated for etiology following a prespecified detailed protocol. Stroke subtypes were defined according to the Trial of Org 10172 in Acute Stroke Treatment (TOAST) criteria. All patients have provided written informed consent. This study was approved by the Research Ethics Committee of the University of Tartu (license 302/M-23). A detailed description and results of the study is provided in Vibo *et al* 2021.^[75](#_ENREF_75)^

Unrelated Estonian Biobank (EstBB) participants without any stroke were used as controls, without any overlap with the controls used in the EstBB analysis.

**Geisinger Ischemic Stroke cohort in MyCode Biobank (GEISINGER)**

The Geisinger MyCode Community Health Initiative is a health system-based population representing a geographically defined population who visit Geisinger clinics from East and Central Pennsylvania and is enrolled in the MyCode genotyping and exome sequencing program.^[76](#_ENREF_76)^ A total of 12,883 IS patients were identified and extracted from the Geisinger Neuroscience Ischemic Stroke (GNSIS) database^[77](#_ENREF_77)^, of which 1,184 patients were enrolled in the Phase I MyCode program and met the inclusion/exclusion criteria. Briefly, this study cohort comprised all consecutive patients aged 18 or higher with IS admitted to Geisinger Health System from September 2003 to May 2019. In cases of multiple encounters due to recurrent cerebral infarcts, the first hospital encounter was considered as the index event. Only first-time IS patients with focal neurological deficit persisting for ≥ 24 hours were selected for analysis. We excluded patients based on the following: 1) patients who had a previous history of stroke outside of Geisinger, 2) patients younger than 18 years at the time of IS, and 3) patients without baseline clinical risk factors recorded in the EHR. All patients had European ancestry (EUR) validated by principal component analysis (PCA)^[78](#_ENREF_78)^ and MRI data to confirm the diagnosis. We also identified 19,806 MyCode patients with index age ≥69 but without the *International Classification of Diseases (ICD)*, *Ninth or Tenth Revision* codes for IS. The genome-wide genotyping and imputation was conducted by Regeneron Genomics Center. GWAS was carried out in a case-control (n=1184 for cases) design by considering all Geisinger MyCode patients with age ≥69(n=19806), or ≥79(n=7484), and without any stroke-related *ICD9* or *ICD10* codes, as low-risk control. The age cutoffs of 69 and 79 for controls were based on mean age of onset for cases which is 59 in our cohort. As this design follows younger cases versus older controls, we expected to have 50% of controls having index age of 10 years or 20 years older than the onset age of cases. A linear mixed regression model (SAIGE) with saddle point approximation, adjusted for covariates (age, sex and five major PCs) was conducted to account for the relatedness and case-control imbalance. We have previously shown that PRS augments stroke subtyping in this retrospective cohort.^[78](#_ENREF_78)^

**Copenhagen City Heart Study (CCHS)**

This prospective study of the Danish general population was initiated in 1976–78 with follow up examinations in 1981–83, 1991–94, and 2001–03.^[79](#_ENREF_79),[80](#_ENREF_80)^ Data collection included a questionnaire, a physical examination, and blood sampling for biochemical and DNA analyses. We included 8,228 unrelated individuals without any stroke at baseline who gave blood for biochemical and DNA analyses at the 1991–94 examination; among these, 1,508 developed all-cause stroke during follow-up.

**Danish Twin Registry (DTR)**

The Danish Twin Registry (DTR) sample included 976 individuals collected as part of the study of Middle-Aged Danish Twins (MADT, N=733) and the Longitudinal Study of Aging Danish Twins (LSADT, N=243).^[81](#_ENREF_81)^ MADT was initiated in 1998 and includes 4,314 twins randomly chosen from the birth years 1931-1952. Surviving participants were revisited from 2008 to 2011, where the blood samples and survey data used in the present study were collected. LSADT was initiated in 1995 and includes twins aged 70 years and older. Follow-up assessments were conducted every second year through 2005. The individuals included here all participated in the 1997 assessment, where blood samples and survey data were collected from same sex twin pairs. GWAS data is available for a total of 1968 MADT and LSADT participants.

Incident stroke cases were defined as individuals registered with one or more of the ICD-10 codes I61 and I63 for the first time ever after blood sampling. Controls were defined as individuals who were not registered with any of the diagnoses I60-69 and who in the survey answered no to self-reported stroke.

Information on registry diagnoses were obtained from the Danish National Patient Registry, which contains discharge diagnoses for all inpatients admitted to Danish hospitals from 1977 onwards and for outpatients from 1995 onwards.^[82](#_ENREF_82),[83](#_ENREF_83)^ In the present study, we included primary and secondary diagnoses for inpatients and outpatients. Individuals were followed in the Danish National Patient Registry until March 2014.

If data were available for both twins of a twin pair, one twin was randomly selected per pair and included in the study.

Written informed consents were obtained from all participants. Collection and use of biological material, and survey and registry information were approved by the Regional Scientific Ethical Committees for Southern Denmark, and the study was registered at Research & Innovation Organization at University of Southern Denmark (registration number 10.874), who approves all scientific projects for University of Southern Denmark according to the Data Protection Regulation.

**Second Manifestations of ARTerial disease (SMART)**

The Second Manifestations of ARTerial disease (SMART) study is an ongoing prospective cohort at the University Medical Center Utrecht, The Netherlands of patients between ages 18–79. Several informations were obtaines at inclusion trough questionnaires on medical history, history of vascular disease (coronary artery disease, cerebrovascular disease, peripheral arterial disease, abdominal aortic aneurysm), cardiovascular risk factors (e.g. hypertension, hyperlipidemia, smoking, alcohol consumption, physical activity) and medication use. Blood pressure, height and weight were also measured.

**ASPirin in Reducing Events in the Elderly (ASPREE)**

This is secondary data analysis of community-dwelling Australian participants aged ≥70 years in the randomised, double-blind, placebo-controlled ASPirin in Reducing Events in the Elderly (ASPREE, n = 19,114) clinical trial and an associated cohort sub-study—the ASPREE Longitudinal Study of Older Persons (ALSOP, n = 14,892). ASPREE recruitment occurred between March 2010 and December 2014. Active enrolled Australian ASPREE participants (n = 16,439) were invited to participate in ALSOP ∼3–6 months after being recruited to ASPREE. Eighty-nine percent (n = 14,892) of all Australian ASPREE participants (n = 16,703) completed a first wave ALSOP questionnaire set, and most (>85%) within 15 months of enrolling in ASPREE. ALSOP questionnaires examined factors related to general health such as eyesight, hearing and oral health, and lifestyle and socioeconomic factors including social health and health behaviours. Participants were eligible for inclusion in our analyses if they were classified as community-dwelling (living at home, with family, friends or spouse) and complete baseline data were available from ASPREE and ALSOP to capture social isolation, social support, loneliness, age, gender and at least one CVD risk factor, thus resulting in a total of 11,498 participants. Ethics approval for ASPREE and ALSOP were obtained through the Monash University Human Research Ethics Committee. Both studies were designed in accordance with the National Health and Medical Research Council Guidelines on Human Experimentation, and conducted in compliance with the Declaration of Helsinki.

The study included genotyped participants from the ASPREE trial. The design and results of the trial have been reported previously.^[84-87](#_ENREF_84)^ Briefly, ASPREE was a randomized double-blind placebo-controlled clinical trial investigating the effect of daily 100mg aspirin on disability-free survival over a median follow-up of 4.7-years (interquartile range 3.6 to 5.7 years). In total, 19,114 individuals aged ≥70 years (≥ 65 years for US minorities) were recruited. Participants were only included when they did not have prior cardiovascular events (including previous diagnosis of myocardial infarction, heart failure, angina pectoris, stroke, diagnosis of atrial fibrillation, or systolic blood pressure ≥180mmHg) and were free from dementia or physical disability at enrolment. All participants provided written informed consent. The ASPREE study was approved by local Ethics Committees and registered on Clinicaltrials.gov (NCT01038583). Informed consent for genetic analysis was obtained, with ethical approval from the Alfred Hospital Human Research Ethics Committee (390/15) and site-specific Institutional Review Boards (US).

ASPREE stroke endpoint: The definition of stroke was based on World Health Organization definition and included imaging by computer tomography or magnetic resonance imaging in the majority of cases.^[19](#_ENREF_19),[88](#_ENREF_88)^ All cases of Ischemic Stroke were further divided into subtypes of large vessel, small vessel, cardioembolic, and undetermined.^[19](#_ENREF_19),[85](#_ENREF_85)^ Undetermined stokes had undetermined causes, multiple causes identified, or an incomplete evaluation made. Fatal stroke was defined as any death in which the underlying cause was an obstruction or rupture in the intracranial or extracranial cerebral arterial system. All stroke events were assessed by the Adjudication Committee; blinded to the identity of participants and study treatment group assignment, as described previously.^[85](#_ENREF_85),[87](#_ENREF_87)^

**German_stroke cohorts**

***Prospective Cohort with Incident Stroke study (PROSCIS-B)***

The prospective cohort with incident stroke (PROSCIS) study is a prospective hospital-based cohort study conducted at two tertiary stroke centres in Germany.(clinicaltrials.org/NCT01363856).^[89](#_ENREF_89)^ PROSCIS-B is conducted at the Center for Stroke Research Berlin, Charité University Hospital, Germany, and enrolled patients between January 2010 and June 2013. The study enrolled patients aged 18 years or older with first ever acute stroke within the last 7 days. Key inclusion criteria included: (1) a diagnosis of ischaemic stroke, primary intracranial haemorrhage, or venous sinus thrombosis according to the WHO criteria, and (2) written informed consent as documented by patient or legal guardian prior to study participation. Key exclusion criteria included: (1) prior stroke (definition according to WHO criteria); (2) brain tumour or brain metastasis; and (3) participation in an intervention/AMG study. Baseline assessments included: a structured interview that collected information about demographic variables, living situation, functional pre-stroke outcome, lifestyle habits, health and family history, as well as medication before stroke provided by the patient or the next of kin and cognitive function before and after stroke. Clinical examinations included anthropometric measures and stroke severity. Vascular and cardiological examinations included standardized physiological measures of blood pressure, electrocardiography, and brain and vessel imaging. All participants provided informed consent and protocols were approved by the institutional review board. Ischemic stroke subtypes were determined according to TOAST criteria based on relevant clinical and imaging data by stroke physicians. Genotyping was performed at the using the Illumina Global screening array (GSA) v2. After initial QC, removal of population outliers, removal of related individuals and filtering on sample and SNP call rate (>99%), imputation to the HRC reference panel was performed using the Michigan imputation server.

***Munich Stroke sample***

Cases were consecutive European Caucasians recruited from a single tertiary level stroke center at LMU Hospital, Ludwig-Maximilians-University, Munich between 2009 and 2017. All participants provided informed consent and protocols were approved by the institutional review board. Ischemic stroke subtypes were determined according to TOAST criteria based on relevant clinical and imaging data by stroke physicians. Genotyping was performed at the Core Facility NGS, Helmholtz Zentrum München, Neuherberg, Germany using the Illumina Global screening array (GSA) v2. After initial QC, removal of population outliers, removal of related individuals and filtering on sample and SNP call rate (>99%), imputation to the HRC reference panel was performed using the Michigan imputation server.

***SICFAIL Study***The Stroke-Induced Cardiac FAILure in mice and men (SICFAIL) study is a prospective hospital-based cohort study comprising consecutive patients with acute ischemic stroke (IS) recruited at the Stroke Unit of the Department of Neurology, University Hospital Würzburg, Germany between January 2014 and February 2017. Inclusion criteria were diagnosis of IS according to the World Health Organization definition (LIT: Hatano S. Experience from a multicentrestroke register: a preliminary report. Bull World Health Organ 1976; 54:541–553.), age ≥18 years and provision of informed consent. Patients participating in an acute intervention study were excluded. The main aim of the SICFAIL study is to describe the natural course of cardiac dysfunction after IS with details of the study design published previously (clinical trial registration: DRKS00011615) (LIT Heuschmann PU et al.).^[90](#_ENREF_90)^ All patients underwent routine diagnostic and etiological workup during the acute treatment phase, including neuroimaging (computed tomography or magnetic resonance imaging), vascular imaging (ultrasound and/or computedtomography or magnetic resonance imaging angiography as judged necessary by the physician in charge), 12-lead electro-cardiography, electrocardiography monitoring and routine blood sampling. Trans-thoracic echocardiography, transoesophageal echocardiography and holter monitoring were performed as part of clinical routine. Demographics, co-morbidities, pre-stroke functional status and lifestyle factors were recorded at baseline. Patients were followed regularly up to 5 years after the event. Etiology of ischemic stroke was classified according to the Trial of ORG 10172 in Acute Stroke Treatment (TOAST) criteria.^[19](#_ENREF_19)^ Classification was in-dependently assessed by specifically trained physicians and interrater reliability of the TOAST classification was good (AC1 coefficient: 0.83). The SICFAIL study was supported by the German Ministry of Research and Education within the Comprehensive Heart Failure Centre Würzburg (grant numbers BMBF 01EO1004 and01EO1504).

***MONICA/KORA Augsburg Study***

For the German (Munich/Berlin/Würzburg) samples, independent control groups were selected from Caucasians of German origin participating into the population KORAgen study. This survey represents a sex- and age stratified random sample of all German residents of the Augsburg area and consists of individuals 25 – 74 years of age, with about 300 subjects for each 10-year increment. All controls were free of a history of stroke or transient ischemic attack. We included controls from the S3 and S4 cohorts of KORA. S3 participants were genotyped using the Illumina Omni2.5 Array, while S4 participants were genotyped using the Affymetrix Axiom genotyping array. After QC, removal of population outliers, removal of related individuals and filtering on sample and SNP call rate (>99%), imputation to the HRC reference panel was performed separately for S3 and S4 using the Michigan imputation server

**Dutch Stroke cohorts**

***FUTURE Study – Radboudumc, Nijmegen, the Netherlands***

The FUTURE study (“Follow-Up of Transient ischemic attack and stroke patients and Unelucidated Risk factor Evaluation” study), is a prospective cohort study designed to investigate the etiologies and consequences of stroke in a population of individuals between ages 18-50 years. The FUTURE study comprised all consecutive patients with a TIA, ischemic stroke, or ICH, between ages 18 – 50 years, admitted to the Radboud University Medical Centre Nijmegen from January 1, 1980 until November 1, 2010. Only patients with first-ever ischemic stroke were included in the present study. Exclusion criteria were previous stroke or TIA, traumatic hemorrhagic stroke, hemorrhage in known cerebral metastasis or primary brain tumor, cerebral venous sinus thrombosis, subarachnoid hemorrhage or ICH due to known ruptured aneurysm, and retinal infarction. To minimize bias due to changing diagnostic techniques, the World Health Organization definition for ischemic stroke was used. Stroke was defined as focal neurological deficit with no other than a vascular cause persisting for a period of for more than 24 hours.

The assessment of the etiology (modified Trial of ORG 10172 in Acute Stroke Treatment (TOAST) classification) was performed for all cases retrospectively using a validated approach as previously described, as the scale did not exist at the time when a substantial number of our patients experienced their index event.

***Dutch Parelsnoer initiative (PSI) Cerebrovascular Disease Study***

The Dutch Parelsnoer initiative (PSI) Cerebrovascular Disease Study is a large prospective cohort study in which comprehensive clinical data, detailed phenotyping of stroke, imaging data, and biomaterials were collected in a large cohort of stroke patients.[^91^](#_ENREF_91) The PSI is a unique partnership between all eight University Medical Centers in the Netherlands and was established in 2007 by the Netherlands Federation of University Medical Centers. The general aims are to build a strong collaborative infrastructure, to allow all participants to prospectively collect their data, and to store biomaterials in a uniform and standardized format.[^92^](#_ENREF_92) For the present study 1,377 patients ≥ 18 years of age with ischemic stroke were included who were enrolled between September 2009 and November 2014. Ischemic stroke was defined as focal neurologic deficits of sudden onset originating from the brain and persisting for more than 24 hours, in the absence of hemorrhage as confirmed by imaging. Ischemic stroke subtypes were further classified according to the Trial of Org 10172 in Acute Stroke Treatment (TOAST).[^19^](#_ENREF_19) Information on ancestry in patients and controls was obtained by self-report. DNA samples were genotyped on the Illumina GSA platform.

***ODYSSEY Study – Radboudumc, Nijmegen, the Netherlands.***

This study is part of the Observational Dutch Young Symptomatic StrokE studY (ODYSSEY), a Dutch multicenter prospective cohort study on the risk factors and prognosis of patients with a first-ever, ischemic stroke, TIA or intracerebral hemorrhage (ICH) aged 18-49 years. For this study, we included 466 patients with ischemic stroke from the Radboudumc Nijmegen site of the study. In short, our study comprises consecutive patients aged 18-49 years with first-ever symptomatic ischemic stroke defined as the occurrence of acute focal neurological deficits *with* radiological evidence of cerebral ischemia. Patients were included between May 2013 until end of inclusion in February 2021.

Patient’s medical files, including risk factors, the cause of ischemic stroke and TIA, were systematically assessed for all patients according to the modified TOAST criteria, (including a subdivision into high-risk and medium-risk sources of cardio-embolism and in large artery atherosclerosis and likely atherothrombotic disease). Atherothrombotic stroke was defined as having (1) an ipsilateral internal carotid stenosis of >50% (in NASCET criteria), or (2) an ipsilateral stenosis of >50% of another intra/extracranial artery, or (3) a mobile thrombus in the aortic arch. Likely atherothrombotic stroke was defined as (1) an ipsilateral internal carotid stenosis of <50%, or (2) an ipsilateral stenosis of <50% of another intra/extracranial artery, or (3) aortic arch plaques >4 mm in thickness without a mobile component, or (4) a history of myocardial infarction or coronary revascularization, (5) a history of documented peripheral arterial disease, or (6) at least two risk factors for atherosclerotic disease: arterial hypertension (treated or known blood pressure before stroke >140/90 mm Hg or hypertensive retinopathy), diabetes mellitus (treated or known blood fasting glucose >7 mmol/l), current smoking (or smoking stopped within the last 6 months), high cholesterol (treated or known low-density lipoprotein before the stroke >160 mg/dl or 4,1 mmol/l).

***Dutch stroke study controls***

The Dutch control subjects were population based controls from the Prospective amyotrophic lateral sclerosis (ALS) Study matched for sex, age and geographic region within the Netherlands. The Prospective ALS study in The Netherlands has been described in detail previously.^[93](#_ENREF_93),[94](#_ENREF_94)^ All individuals gave written informed consent and the University Medical Center Utrecht Medical Ethics Committee, Utrecht approved this protocol. The controls were genotyped on Illumina OmniExpress and Illumina GSA.

**China Kadoorie Biobank (CKB)**

China Kadoorie Biobank (CKB) is a study, which investigates the main genetic and environmental causes of common chronic diseases in the Chinese population. During 2004-2008, over 510,000 men and women aged 30-79 years were recruited from the general population in five rural and five urban areas in China with extensive data collection by questionnaire and physical measurements, and with long-term storage of blood samples for future study. The study was approved by the ethical review committee of the Chinese Center for Disease Control and Prevention and the Oxford Tropical Research Ethics Committee, University of Oxford. All participants provided written informed consent forms.^[95](#_ENREF_95)^ Incident stroke events were ascertained through linkage to death and disease registries and the nationwide health insurance system, which records all hospitalized events, among participants with no history of stroke or TIA at baseline. Stroke events were defined according to the International Classification of Diseases 10th revision (ICD-10, I60, I61, I63, I64). Genotyping was performed using a custom-designed Affymetrix Axiom array, with imputation into 1KG3, among a randomly selected subset of 75,719 participants.

**The Korean Cancer Prevention Study-II (KCPS2) Biobank**

The Korean Cancer Prevention Study-II (KCPS2) Biobank is a large blood-based cohort study with long-term follow-up via a unique linkage of routine, medical examinations conducted at health promotion centers across South Korea with records for mortality and hospitalization. The cohort comprises 156 701 participants (94 840 men and 61 861 women) who undertook routine health assessments during 2004 and 2013, provided blood samples and informed consent for long-term prospective follow-up. We prospectively identified incident strokes and ischemic stroke cases from insurance claims reported to the National Health Insurance System. We ascertained nonfatal or stroke events, defined according to the International Classification of Diseases 10th revision (ICD-10, I60– I69).

**The Joinville Stroke Biobank (JSB)**

The Joinville Stroke Biobank (JSB) has its origin in the Joinville Stroke Registry (JOINVASC), a cohort aimed for studies on epidemiological aspects of stroke in Brazil and is currently maintained with University of the Region of Joinville (Univille) resources. The phenotypic data are extracted from the Joinville Stroke Registry. The Joinvile Stroke Registry (JOINVASC) is an ongoing population-based stroke data bank started in 2005 and supported by municipal law since 2013. The city has two stroke centers, four general hospitals with computed tomography (CT) available 24/7, and one public rehabilitation care facility, totaling 1078 beds. The registry uses the ideal methodology proposed by Sudlow and Warlow^[96](#_ENREF_96)^ as well as the Stroke-Steps modular program proposed by WHO (first step for all hospital cases, second step for checking of death certificates and third step to ascertain mild events) to ascertain stroke events. After obtaining written informed consent from all patients or their relatives/legal responsible, the JOINVASC research nurses routinely record clinical, laboratorial, and sociodemographic data, as well as electrocardiographic and radiological tests´ results. Stroke is defined as the presence of signs of sudden focal or global cerebral dysfunction that lasts longer than 24 h without any apparent non-vascular cause. TIA is defined as a sudden acute loss of cerebral or ocular function, with symptoms lasting less than 24 h, which could be indicative of an embolic or atherothrombotic disease after appropriate investigation.

**GENERACION Project**

The ischemic stroke patients were recruited if they had a measurable neurologic deficit on the NIHSS within 6 hours of last known normal, had a stroke diagnosis performed by an experienced neurologist at each center and confirmed by neuroimaging, were older than 18 years of age, and were recruited at one of the 14 hospitals included in the study. Etiologic subgroups were classified following TOAST criteria. These patients were recruited as part of the GENISIS,^[97](#_ENREF_97)^ GODS,^[98](#_ENREF_98)^ and CONIC^[99](#_ENREF_99)^ projects.

Controls were subjects without a history of ischemic stroke, older than 18, who declared they were free of neurovascular diseases by direct interview before recruitment. The control cohort was collected in primary care centers from Barcelona city and in hospitals throughout Spanish territory as a part of the GCAT,^[100](#_ENREF_100),[101](#_ENREF_101)^ CONIC,^[99](#_ENREF_99)^ GRECOS,^[102](#_ENREF_102)^ and ISSYS^[103](#_ENREF_103)^ projects.

***Genetics of Early Neurological Instability after Ischemic Stroke (GENISIS) cohort***

Genetics of Early Neurological Instability after Ischemic Stroke (GENISIS) is an international study currently recruiting patients from four different locations: United States, Finland, Poland, and Spain. The inclusion criteria for the GENISIS study are IS patients (age ≥ 18 years) collected from 2003 to 2017 with a measurable neurologic deficit on the NIHSS within 6 hours of last known normal. Patients who received endovascular thrombectomy, or for whom consent and/or a blood sample could not be obtained were excluded. For our study we only included Spanish patients.

***The Genetic contribution to functional Outcome and Disability after Stroke (GODS) cohort***

The Genetic contribution to functional Outcome and Disability after Stroke (GODS) project is a study that aimed to find genetic factors associated with stroke outcome. All participants met the following criteria: (1) European descent, aged >18 years, diagnosis of IS in the anterior vascular territory; (2) assessed by a neurologist during the acute phase of stroke; (3) initial stroke severity >4, according to the National Institutes of Health Stroke Scale (NIHSS); (4) information on post-stroke functional status at 3 months (or alternatively between 3 and 6 months); (5) evidence of acute IS in a neuroimaging study; (6) lack of concomitant disease. Individuals with stroke recurrence during the follow-up period were excluded, in addition to posterior vascular territory and lacunar strokes.

***The CONtrol ICtus (CONIC) cohort***

The CONtrol ICtus (CONIC) study is a national study focus on find new genetic risk factors for ischemic stroke, it is a case-control matched study. Control participants were recruited between 2007 and 2008. All controls were older than 65 years of age and declared free of dementia, neurovascular and/or cardiovascular disease, as evaluated by self-description during a direct interview before recruitment. Subjects with a history of first and/or second-degree neurovascular disorder were also excluded from the study. The stroke cases were admitted to the emergency department of a university with a documented middle cerebral artery (MCA) occlusion on transcranial Doppler ultrasonography (TCD) and received tPA in a standard 0.9-mg/kg dose (10% bolus, 90% continuous infusion over 1 hour) within 4.5 hours of symptom onset following National Institute of Neurological Disorders and Stroke (NINDS) recommendations.

***The Genotyping RECurrence Risk Of Stroke (GRECOS) cohort***

The Genotyping RECurrence Risk Of Stroke (GRECOS) project is a national study that aimed to find genetic factors associated with recurrence after stroke. Control participants were relatives of patients (wife or husband, without any consanguinity between cases and controls) and healthy volunteers visiting the same hospital for routine testing. They were >65 years of age and classified as free of neurovascular and cardiovascular history and family history by direct interview before recruitment.

***The Investigating Silent Stroke in hYpertensives: A magnetic resonance imaging Study (ISSYS) cohort***

The Investigating Silent Stroke in hYpertensives: A magnetic resonance imaging Study (ISSYS) is an observational prospective study in hypertensive participants to determine the prevalence of silent or magnetic resonance imaging (MRI)–defined brain infarcts and cognitive impairment. This cohort comprises 1000 nondemented individuals, aged 50 to 70 years old, and diagnosed of essential hypertension at least one year before inclusion in the ISSYS study

***GCAT Genomes for Life Study cohort***

GCAT|Genomes for Life Study is a long-term project that was set up to integrate and assess the role of epidemiological, environmental and omic factors (genomic, metabolomic, proteomic, epigenomic) in the development of chronic diseases. GCAT aims to assess the prevalence of risk factors and their association with disease incidence over time. The GCAT cohort is a prospective collection recruited from the general population of the north-east region of Spain, Catalonia. The GCAT Study have recruited 20 000 participants aged 40–65 years. Participants complete a self-administered computer-based questionnaire that collects data on a large number of lifestyle and health factors that are of interest in epidemiological and genetic studies. Participants who agreed to take part in the study completed a self-administered computer-driven questionnaire, and underwent blood pressure, cardiac frequency and anthropometry measurements. Participants will be followed for 20 years after recruitment.

**AIIMS-DELHI (Indian stroke GWAS)**

The Indian Stroke GWAS study is a multicentric hospital-based case-control study where 8 teaching hospitals from Northern, Southern and North-Eastern India recruited 4,088 participants, including 1,609 stroke cases.^[104](#_ENREF_104)^ List of the 8 hospitals participating in the study is given below: (1) AIIMS, All India Institute of Medical Sciences, New Delhi, India, (2) PGI Rohtak, Pandit Bhagwat Dayal Sharma Post Graduate Institute of Medical Sciences or PGIMS Rohtak, (3) Safdarjung, Safdarjung Hospital, New Delhi, (4) R and R, Army Hospital (Research And Referral, New Delhi, (5) Gangaram, Sir Gangaram Hospital, New Delhi, RML, (6) Dr. Ram Manohar Lohia Hospital in New Delhi, (7) Sree Chitra Tirunal Institute for Medical Sciences and Technology, (8) NEIGHRHIMS, North Eastern Indira Gandhi Regional Institute of Health and Medical Sciences, Shillong, India.

Stroke was diagnosed using guidelines set by the World Health Organization by trained neurologists and was primarily of vascular origin. Stroke-free status was assessed by a well-validated questionnaire.^[105](#_ENREF_105)^ Each study site recruited its controls from the same site after ethnicity matching. Genome-wide genotyping was performed on an Illumina platform using the genome screen array version 2.0 (with additional multidisease content). Additional details on genotyping, imputation, statistical testing etc have been published previously.^[104](#_ENREF_104)^

**Tohoku Medical Megabank**

The Tohoku Medical Megabank (TMM) Project is composed of a population-based adult cohort study, the TMM Community-Based Cohort Study (TMM CommCohort Study) and a birth and three-generation cohort study, the TMM Birth and Three-Generation Cohort Study (TMM BirThree Cohort Study).^[106](#_ENREF_106),[107](#_ENREF_107)^ The aim of the TMM CommCohort Study was to assess the long-term impact of the Great East Japan Earthquake (March 11, 2011) on disaster victims and gene-environment interactions on the incidence of multifactorial diseases, such as cancer and cardiovascular diseases.^[107](#_ENREF_107)^

**GIGASTROKE follow-up studies**

**Million Veteran Program**

The Million Veteran Program (MVP) is an ongoing longitudinal cohort study that began in 2011 to study genetic and non-genetic determinants of health and disease. Active users of the Veterans Health Administration healthcare system learn of MVP via an invitational mailing and/or through MVP staff while receiving clinical care with informed consent and HIPAA authorization as the only inclusion criteria. Clinical and demographic characteristics were obtained from electronic health records within the VA’s Corporate Data Warehouse (CDW) and supplemented by the MVP Baseline Survey when age or sex was missing.^[108](#_ENREF_108)^ Genotyping was performed with a customized Affymetrix Axiom Biobank Array,^[109](#_ENREF_109)^ and imputation was carried out with a hybrid imputation panel comprised of the African Genome Resources panel (https://imputation.sanger.ac.uk/?about=1#referencepanels) and 1000 Genomes (p3v5). Population-specific principal components (PCs) were computed using EIGENSOFT v.6. The harmonized race/ethnicity and genetic ancestry (HARE) approach was used to assign individuals to two mutually exclusive groups: 1) non-Hispanic White (European ancestry), 2) non-Hispanic Black (African ancestry).^[110](#_ENREF_110)^ Kinship was inferred using KING v.2.0. For each pair of relatives (kinship coefficient ≥0.0884), one individual was excluded.

The replication cohort for follow-up analyses spanned the period beginning in 2011 to the end of 2018. Incident acute ischemic stroke (AIS) events were identified from VA EHR data using both inpatient and outpatient ICD-9 (433.x1, 434.x, 436.x, excluding 434.x0) and ICD-10 (I63.x cerebral infarction) codes, or analogous codes in data from the Centers for Medicare and Medicaid (CMS). Date of AIS was defined as the first occurrence of ischemic stroke codes. Participants who had an ischemic stroke prior to enrollment were excluded, and the absence of ischemic stroke codes was assumed to be no event. Follow-up for each participant was measured in days since enrollment until one of three censoring events: AIS, administrative censoring (Jan 1st, 2019), or death. The final analysis cohort included 403,489 European and 107,343 African ancestry participants at baseline with a mean age of 63.2 and 57.0 years, 92% and 86% male, median (5^th^ - 95^th^ percentile) follow-up of 3.8 (0.4 – 6.8) and 3.8 (0.5 – 6.7) years, and among whom 8,392 and 2,227 experienced an incident AIS during follow-up, respectively.

**Estonian Biobank**

See above for details on Estonian Biobank. In the replication we analyzed 1571 new cases of stroke and 4062 matched controls that had been recruited to the Estonian biobank during 2018-2021 (not included in the initial GWAS). The genotyping and data processing was identical with that of the EstBB cohort described above. Significant variants from the GWAS meta-analyses were tested with Regenie (v2.2.4) logistic regression, including age, sex and PC 1-5 as covariates in the analyses.

**Penn Medicine Biobank**

The Penn Medicine Biobank is a genomic and precision medicine cohort that recruits patients from clinical practice sites throughout the University of Pennsylvania Health System. Participants actively consent to allow the linkage of biospecimens to data from their longitudinal electronic health record. Currently >80,000 participants are enrolled in the PMBB. A subset of 43,623 individuals have undergone whole exome sequencing and genotyping through a collaboration with the Regeneron Genetics Center. Stroke cases were identified using ICD 9 and 10 diagnosis codes reported in either 1 inpatient visit or 2 outpatient visits.

**Mass General Brigham Biobank**

The Mass General Brigham Biobank (MGB) is an ongoing prospective clinical research cohort of patients of Mass General Brigham, the parent organization of Massachusetts General Hospital (MGH) and Brigham and Women’s Hospital (BWH) in Boston, Massachusetts, USA. All patients aged 18 years or older presenting to any of the MGB clinics consenting to broad research are included. Patients are recruited in-person at MGH and BWH and online through an electronic patient gateway. The MGB Biobank provides banked samples (plasma, serum, DNA, buffy coats), electronic health record (EHR) data, imaging, survey data on lifestyle, environment, family history, and genetic data. Recruitment has been ongoing since 1998 and to date, more than 133,000 patients have been included in the Biobank.

Subjects consented from 2009-2021 with sufficient quantity and quality of stored DNA samples were successively genotyped. Genotyping was performed in batches at the MGB Translational Genomics Core (Boston MA, USA) and at the Broad Institute (Cambridge MA, USA), using Illumina arrays (MEGA, MEGA-Ex, GSA-24v2-0 + Multi-Disease). Genotyping on the MEG arrays was performed between 2017-2019, on GSA arrays between 2019-2021. Array-based genotypes with a call rate of 99% or higher and without sex mismatch were retained and imputed at the Michigan imputation server separately on each batch, using the Minimac3 algorithm and the 1000 Genome reference panel. At study date, genotypes for 56,000 patients were available.

To build the replication cohort, the MGB Biobank database was queried for genotyped patients. Cases were identified by presence of any type of ischemic stroke in their EHR as defined by ICD9 (433.01, 433.11, 433.21, 433.31, 433.81, 433.91, 434.01, 434.11, 434.91, 436) or ICD10 code (G46.3, G46.4, I63.*, I67.81, I67.82). Individuals previously included in the Stroke Genetics Network (SiGN) study were excluded (n=84). Controls were defined by absence of ischemic stroke codes. We aimed to include stroke-free individuals with the most up-to-date EHR data to prevent the inclusion of subjects that had a stroke outside the MGB system since consent to the Biobank. For this reason, we included only individuals with at least one encounter in the last 12 months, and at least two encounters with the MGB system in total.

The genotypes for cases and controls were extracted from each batch and quality control was performed using PLINK v.1.9. Samples with high missing rates (call rate < 97%) and sex mismatches were excluded. A pruned subset of autosomal markers not in linkage disequilibrium (r^2^ > 0.1) was generated. These markers were used to remove one individual from each related pair (IBD sharing > 0.25) and to generate the top 10 principal components (PCs) for selection of individuals of European ancestry.

3,745 ischemic stroke cases and 28,281 stroke-free controls resulted from the steps outlined above. Logistic regression models were run separately on each batch of imputed genotypes for each target SNP selected from the discovery cohort using PLINK v1.9. Sex, age, and the first 10 PCs were included as covariates in the models. To combine the results obtained from the separate batches, we performed a fixed-effect meta-analysis using METAL (STDERR scheme on Betas and SE), after selecting high quality top/tag SNPs (MAF > 0.01, R2 > 0.4, Hardy-Weinberg equilibrium p-value > 1e-6, SNP call rate > 97%).

**Stroke Investigative Research and Educational Network (SIREN)**

The SIREN study is a multicenter case-control study involving 15 medical centers in Ghana and Nigeria initiated in 2014.^[111](#_ENREF_111),[112](#_ENREF_112)^ It is the largest study of stroke in Africa with the overall goal of evaluating the premier genetic and environmental risk factors for stroke in Africa. The study enrolled consented stroke patients who were ≥18 years old with first clinical stroke within 8 days of current symptom onset confirmed by neuroimaging with CT or MRI scan within 10 days of symptom onset. Controls were stroke-free individuals from the same catchment area as the stroke cases, identified and recruited through the community engagement core and matched to stroke cases by sex, age (±5 years) and ethnicity. Consented stroke cases and controls were monitored and followed up for 1 year after enrolment.

**Helsinki Ischemic Stroke Genetics Study**

Helsinki Ischemic Stroke Genetics Study was designed for investigating genetic factors underlying ischemic stroke in the Finnish population and in the long-term to be incorporated to multicenter multinational similar datasets. Ischemic stroke cases were recruited from 2012 to 2017 from the Helsinki University Hospital, Department of Neurology which is the only neurological emergency unit for a population of 1.7 million inhabitants. 1848 patients with positive neuroimaging findings for a new-onset brain infarction were included. Stroke subtyping was performed according to the Trial of Org 10172 in Acute Stroke Treatment (TOAST) classification.

Control samples were obtained from the national FINRISK study 2012 cohort.^[113](#_ENREF_113)^ Only participants residing in the same geographic area (Greater Helsinki region) and without history of ischemic or hemorrhagic stroke based on the National Hospital Discharge Register and the National Causes-of-Death Register (ICD-10 codes I61, I63.0-I63.5, I63.7-I63.9, I64 or respective in the earlier ICD versions) were included. All case and control subjects are of white, Caucasian origin.

The genotyping was done on the Illumina HumanCoreExome or Illumina Global Screening array. After initial quality control, the data was imputed with Sequencing Initiative Suomi (SISu) v3 imputation reference panel following the FIMM Sequencing Informatics genotype imputation workflow.^[114](#_ENREF_114)^

The Helsinki Ischemic Stroke Genetics study and FINRISK study have been approved by the Ethics Committee of Medicine, Helsinki University Hospital and the Coordinating Ethics Committee of Helsinki University Hospital, respectively. All the participants or their legal representative have provided a written informed consent.

**BioVU**

BioVU is Vanderbilt University Medical Center’s DNA repository linked to the synthetic derivative – a database of de-identified electronic health records.[^115^](#_ENREF_115) The samples included in this study were genotyped on multi-ethnic genotyping array (MEGA) platform from Illumina. We used supervised learning mode in Admixture to calculate ancestry coefficients for our MEGA dataset (94489 samples). 1000 genome phase 3 data, 2504 reference individuals (5 ancestries), is used as training samples. The 5 ancestry fractions (K_EUR ,K_AFR ,K_AMR ,K_EAS , and K_SAS) are produced and we used a threshold of K_EUR>= 0.9 for EUR and K_AFR >=0.8 for AFR. However, only European ancestry partcipants were included in this analysis. Genotyped dataset was cleaned initially using cut off of sample calling rate >=95% and variants calling rate>=95%. Before imputation, variants with MAF<0.1% and variants with allele frequency inconsistent with gnomAD database were removed. Imputation was performed using Minimac4 and 1000 Genomes Phase 3 v5 reference panel with the Michigan Imputation Server (https://www.nature.com/articles/ng.3656). Variants with R2 >= 0.3 were selected for downstream analysis.

**FinnGen study**

See description above. For follow-up, data from autumn 2021 (data freeze 8) was used, with 124,217 genotyped individuals from hospital biobanks.

**Clinical Research Collaboration for Stroke in Korea (CRCS-K) and Korea Biobank Array (KBA) project**

***Clinical Research Collaboration for Stroke in Korea (CRCS-K)***

The Clinical Research Collaboration for Stroke in Korea (CRCS-K) is a collaborative stroke research group and its key component is a multicenter prospective registry of acute stroke patients who admitted to 18 academic and regional stroke centers in Korea.^[116](#_ENREF_116),[117](#_ENREF_117)^ CRCS-K was first established in 2006 as a fifth division of Clinical Research Center for Stroke project (CRCS-5) with the purpose of conducting epidemiological researches and providing information of the status of stroke care in Korea.

Investigators started to collect information on consecutive acute stroke patients based on web-database system (http://www.strokedb.or.kr/ecrf/) in April 2008. In November 2009, first 5 centers initiated prospective capture of stroke outcomes and was extended to all the participating centers in January 2011, including event outcomes and modified Rankin Scale (mRS) scores at 3 months and 1 year after stroke. Outcome capture is conducted by dedicated stroke coordinators of individual participating centers, via review of medical record or structured face to face or telephone interview.

Total number of stroke patients who were registered to the CRCS-K registry exceeded 98,000 by March 2022. Data fields of the registry widely cover information for demographics, risk factors, stroke characteristics including etiology work-ups and laboratory tests, acute stroke management, secondary stroke prevention and post stroke outcomes.

From July 2017 to November 2019, blood samples of early onset stroke patients (younger than 55 years old) were deposited to the National Biobank of Korea (NBK) and a total of 1,120 early onset stroke patients were genotyped using the Korea Biobank Array, which is a SNP microarray optimized for the Korean population and comprises about 827K markers including >200K rare-frequency or functional variants.^[118](#_ENREF_118)^

***Korea Biobank Array (KBA) project***

Korea Biobank Array (KBA) project was initiated in 2014 to characterize genetic variation influencing complex traits such as T2D and obesity tin the Korean population.^[118](#_ENREF_118)^ The National Institute of Health, Republic of Korea designed the KBA, a fully customized SNP microarray, comprising approximately 830K markers with optimal tagging of common variants in East Asian populations along with > 200K functional variants. The project genotyped about 160,000 individuals from the Korean Genome and Epidemiology Study (KoGES),[^119^](#_ENREF_119) using the KBA. Among them, 77,583 individuals were used as control samples for CRCS-K and KBA case-control genome-wide association study (GWAS).

# 3. Study-specific acknowledgements

**GIGASTROKE studies previously included in MEGASTROKE**

**METASTROKE**

Australian population control data were derived from the Hunter Community Study. We also thank the University of Newcastle for funding and the men and women of the Hunter region who participated in this study. This research was funded by grants from the Australian National and Medical Health Research Council (NHMRC Project Grant ID: 569257), the Australian National Heart Foundation (NHF Project Grant ID: G 04S 1623), the University of Newcastle, the Gladys M Brawn Fellowship scheme, and the Vincent Fairfax Family Foundation in Australia. Elizabeth G Holliday was supported by a Fellowship from the National Heart Foundation and National Stroke Foundation of Australia (ID: 100071). Bio-Repository of DNA in Stroke (BRAINS) is partly funded by a Senior Fellowship from the Department of Health (UK) to P Sharma, the Henry Smith Charity and the UK-India Education Research Institutive (UKIERI) from the British Council. Genetics of Early Onset Stroke (GEOS) Study, Baltimore, USA was supported by the NIH Genes, Environment and Health Initiative (GEI) Grant U01 HG004436, as part of the GENEVA consortium under GEI, with additional support provided by the Mid-Atlantic Nutrition and Obesity Research Center (P30 DK072488), and the Office of Research and Development, Medical Research Service, and the Baltimore Geriatrics Research, Education, and Clinical Center of the Department of Veterans Affairs. Genotyping services were provided by the Johns Hopkins University Center for Inherited Disease Research (CIDR), which is fully funded through a federal contract from the NIH to the Johns Hopkins University (contract number HHSN268200782096C). Assistance with data cleaning was provided by the GENEVA Coordinating Center (U01 HG 004446; PI Bruce S Weir). Study recruitment and assembly of datasets were supported by a Cooperative Agreement with the Division of Adult and Community Health, Centers for Disease Control and Prevention and by grants from NINDS and the NIH Office of Research on Women's Health (R01 NS45012, U01 NS069208-01). Heart Protection Study (HPS) (ISRCTN48489393) was supported by the UK Medical Research Council (MRC), British Heart Foundation, Merck and Co (manufacturers of simvastatin), and Roche Vitamins Ltd (manufacturers of vitamins). Genotyping was supported by a grant to Oxford University and CNG from Merck and Co. Jemma C Hopewell acknowledges support from the British Heart Foundation (FS/14/55/30806). The Ischemic Stroke Genetics Study (ISGS) was supported by the NINDS (R01 NS42733; PI Dr Meschia). The Sibling with Ischemic Stroke Study (SWISS) was supported by the NINDS (R01 NS39987; PI Dr Meschia). Both SWISS and ISGS received additional support, in part, from the Intramural Research Program of the National Institute on Aging (Z01 AG000954-06; PI Andrew Singleton). SWISS and ISGS used samples and clinical data from the NIH-NINDS Human Genetics Resource Center DNA and Cell Line Repository (http://ccr.coriell.org/ninds), human subject protocol Nos. 2003-081 and 2004-147. SWISS and ISGS used stroke-free participants from the Baltimore Longitudinal Study of Aging (BLSA) as controls with the permission of Dr Luigi Ferrucci. The inclusion of BLSA samples was supported, in part, by the Intramural Research Program of the National Institute on Aging (Z01 AG000015-50), human subject protocol No. 2003-078. This study used the high-performance computational capabilities of the Biowulf Linux cluster at the NIH (http://biowulf.nih.gov). For SWISS and ISGS cases of African ancestry, a subset of the Healthy Aging in Neighborhoods of Diversity across the Life Span study (HANDLS) were used as stroke-free controls. HANDLS is funded by the National Institute of Aging (1Z01AG000513; PI Michele K. Evans). MGH Genes Affecting Stroke Risk and Outcome Study (MGH-GASROS) was supported by NINDS (U01 NS069208), the American Heart Association/Bugher Foundation Centers for Stroke Prevention Research 0775010N, the NIH and NHLBI's STAMPEED genomics research program (R01 HL087676), and a grant from the National Center for Research Resources. The Broad Institute Center for Genotyping and Analysis is supported by grant U54 RR020278 from the National Center for Research resources. MILANO: Milano - Besta Stroke Register Collection and genotyping of the Milan cases within CEDIR were supported by the Italian Ministry of Health (Grant Numbers: RC 2007/LR6, RC 2008/LR6; RC 2009/LR8; RC 2010/LR8; GR-2011-02347041). FP6 LSHM-CT-2007-037273 for the PROCARDIS control samples. Wellcome Trust Case-Control Consortium 2 (WTCCC2) was principally funded by the Wellcome Trust, as part of the Wellcome Trust Case Control Consortium 2 project (085475/B/08/Z and 085475/Z/08/Z and WT084724MA). The Stroke Association provided additional support for collection of some of the St George's, London cases. The Oxford cases were collected as part of the Oxford Vascular Study which is funded by the MRC, Stroke Association, Dunhill Medical Trust, National Institute of Health Research (NIHR) and the NIHR Biomedical Research Centre, Oxford. The Edinburgh Stroke Study was supported by the Wellcome Trust (clinician scientist award to C Sudlow), and the Binks Trust. Sample processing occurred in the Genetics Core Laboratory of the Wellcome Trust Clinical Research Facility, Western General Hospital, Edinburgh. Much of the neuroimaging occurred in the Scottish Funding Council Brain Imaging Research Centre (www.sbirc.ed.ac.uk), Division of Clinical Neurosciences, University of Edinburgh, a core area of the Wellcome Trust Clinical Research Facility and part of the SINAPSE (Scottish Imaging Network—A Platform for Scientific Excellence) collaboration (www.sinapse.ac.uk), funded by the Scottish Funding Council and the Chief Scientist Office. Collection of the Munich cases and data analysis was supported by the Vascular Dementia Research Foundation. M Farrall and A Helgadottir acknowledge support from the BHF Centre of Research Excellence in Oxford and the Wellcome Trust core award (090532/Z/09/Z). This project has received funding from the European Union’s Horizon 2020 research and innovation programme under grant agreements No 666881, SVDs@target (to M Dichgans) and No 667375, CoSTREAM (to M Dichgans); the DFG as part of the Munich Cluster for Systems Neurology (EXC 2145 SyNergy – ID 390857198) and the CRC 1123 (B3)(to M Dichgans); the Corona Foundation (to M Dichgans); the Fondation Leducq (Transatlantic Network of Excellence on the Pathogenesis of Small Vessel Disease of the Brain)(to M Dichgans); the e:Med program (e:AtheroSysMed) (to M Dichgans) and the FP7/2007-2103 European Union project CVgenes@target (grant agreement number Health-F2-2013-601456) (to M Dichgans). The GWAS component of the VISP study was supported by the United States National Human Genome Research Institute (NHGRI), Grant U01 HG005160 (PI Michèle Sale & Bradford Worrall), as part of the Genomics and Randomized Trials Network (GARNET). Genotyping services were provided by the Johns Hopkins University Center for Inherited Disease Research (CIDR), which is fully funded through a federal contract from the NIH to the Johns Hopkins University. Assistance with data cleaning was provided by the GARNET Coordinating Center (U01 HG005157; PI Bruce S Weir). Study recruitment and collection of datasets for the VISP clinical trial were supported by an investigator-initiated research grant (R01 NS34447; PI James Toole) from the United States Public Health Service, NINDS, Bethesda, Maryland. Control data for comparison with European ancestry VISP stroke cases were obtained through the database of genotypes and phenotypes (dbGAP) High Density SNP Association Analysis of Melanoma: Case-Control and Outcomes Investigation (phs000187.v1.p1; R01CA100264, 3P50CA093459, 5P50CA097007, 5R01ES011740, 5R01CA133996, HHSN268200782096C; PIs Christopher Amos, Qingyi Wei, Jeffrey E. Lee). For VISP stroke cases of African ancestry, a subset of the Healthy Aging in Neighborhoods of Diversity across the Life Span study (HANDLS) were used as stroke free controls. HANDLS is funded by the National Institute of Aging (1Z01AG000513; PI Michele K. Evans). Funding support for WHI-GARNET was provided through the NHGRI GARNET (Grant Number U01 HG005152). Assistance with phenotype harmonisation and genotype cleaning, as well as with general study coordination, was provided by the GARNET Coordinating Center (U01 HG005157). Funding support for genotyping, which was performed at the Broad Institute of MIT and Harvard, was provided by the NIH Genes, Environment, and Health Initiative (GEI; U01 HG004424).

**SiGN**

The SiGN study was funded by a cooperative agreement grant from the US National Institute of Neurological Disorders and Stroke, National Institutes of Health (U01 NS069208). The Base de Datos de Ictus del Hospital del Mar (BASICMAR) Genetic Study was supported by the Ministerio de Sanidad y Consumo de España, Instituto de Salud Carlos III (ISC III) with the grants: Registro BASICMAR Funding for Research in Health (PI051737); GWA Study of Leukoaraiosis (GWALA) project from Fondos de Investigación Sanitaria ISC III (PI10/02064) and (PI12/01238); Agència de Gestió Ajuts Universitaris de Recerca (2014 SGR 1213) and Fondos European Regional Development Funding (FEDER/EDRF) Red INVICTUS-PLUS (RD16/0019/0002). Additional support was provided by the Fundació la Marató TV3 with the grant GODS project. Genestroke Consortium (76/C/2011) Recercaixa’13 (JJ086116). Assistance with data cleaning was provided by the Research in Cardiovascular and Inflammatory Diseases Program of Institute Hospital del Mar of Medical Investigations, Hospital del Mar, and the Barcelona Biomedical Research Park. The Edinburgh Stroke Study was supported by the Wellcome Trust and the Binks Trust. Sample processing occurred in the Genetics Core Laboratory of the Wellcome Trust Clinical Research Facility, Western General Hospital, Edinburgh, UK. Much of the neuroimaging occurred in the Scottish Funding Council Brain Imaging Research Centre (www.sbirc.ed.ac.uk), University of Edinburgh, a core area of the Wellcome Trust Clinical Research Facility and part of the Scottish Imaging Network–A Platform for Scientific Excellence (SINAPSE) collaboration (www.sinapse.ac.uk), funded by the Scottish Funding Council and the Chief Scientist Office. Genotyping was performed at the Wellcome Trust Sanger Institute in the United Kingdom and funded by the Wellcome Trust as part of the Wellcome Trust Case Control Consortium 2 project (085475/B/08/Z and 085475/Z/08/Z and WT084724MA). The Greater Cincinnati/Northern Kentucky Stroke Study (GCNKSS) was supported by the NIH (NS030678). The Austrian Stroke Prevention Study was supported by the Austrian Science Fund (FWF) grant Nos. P20545-P05 and P13180 and I904-B13 (Era-Net). The Medical University of Graz supports the databases of the Graz Stroke Study and the Austrian Stroke Prevention Study. Phenotypic data and genetic specimens collection were funded by the grant from the Polish Ministry of Science and Higher Education for Leading National Research Centers (KNOW) and by the grants from the Jagiellonian University Medical College in Krakow, Poland: K/ZDS/002848, K/ZDS/003844. The Leuven Stroke genetics study was supported by personal research funds from the Department of Neurology of the University Hospitals Leuven. Dr Lemmens is a Senior Clinical Investigator of FWO Flanders (FWO 1841913N). The Lund Stroke Register was supported by the Swedish Research Council (K2010-61X-20378-04-3), The Swedish Heart-Lung Foundation, Region Skåne, Skåne University Hospital, the Freemasons Lodge of Instruction EOS in Lund, King Gustaf V’s and Queen Victoria’s Foundation, Lund University, and the Swedish Stroke Association. Biobank services were provided by Region Skåne Competence Centre (RSKC Malmö), Skåne University Hospital, Malmö, Sweden, and Biobank, Labmedicin Skåne, University and Regional Laboratories Region Skåne, Sweden. The Malmӧ Diet and Cancer Study was supported by the Swedish Research Council (Vetenskapsrådet), Heart and Lung Foundation (Hjärt och Lungfonden), and Swedish Stroke Foundation (Strokeförbundet). The Middlesex County Ischemic Stroke Study (MCISS) was supported by intramural funding from the New Jersey Neuroscience Institute/JFK Medical Center, Edison, NJ, and The Neurogenetics Foundation, Cranbury, NJ. We acknowledge Dr Souvik Sen for his advice and encouragement in the initiation and design of this study.The NOMAS and MIAMISR Cohorts are funded by the grants from the NIH/NINDS: NOMAS (R56NS029993) and SiGN (U01NS069208) and supported by grants from the NINDS (R37 NS029993, R01 NS27517). The Cerebrovascular Biorepository at University of Miami/Jackson Memorial Hospital (The Miami Stroke Registry, Institutional Review Board No. 20070386) was supported by the Department of Neurology at University of Miami Miller School of Medicine and Evelyn McKnight Brain Institute. Biorepository and DNA extraction services were provided by the Hussmann Institute for Human Genomics at the Miller School of Medicine. The Massachusetts General Hospital Stroke Genetics Group was supported by the NIH Genes Affecting Stroke Risks and Outcomes Study (GASROS) grant K23 NS042720, the American Heart Association/Bugher Foundation Centers for Stroke Prevention Research 0775010N, and NINDS K23NS042695, K23 NS064052, the Deane Institute for Integrative Research in Atrial Fibrillation and Stroke, and by the Keane Stroke Genetics Fund. Genotyping services were provided by the Broad Institute Center for Genotyping and Analysis, supported by grant U54 RR020278 from the National Center for Research Resources. The Nurses’ Health Study work on stroke is supported by grants from the NIH, including HL088521 and HL34594 from the National Heart, Lung, and Blood Institute, as well as grants from the National Cancer Institute funding the questionnaire follow-up and blood collection: CA87969 and CA49449. The Oxford Vascular Study was supported by the Wellcome Trust, Wolfson Foundation, Stroke Association, Medical Research Council, Dunhill Medical Trust, NIH Research (NIHR), and NIHR Oxford Biomedical Research Centre based at Oxford University Hospitals NHS Trust and University of Oxford. Dr Rothwell is in receipt of Senior Investigator Awards from the Wellcome Trust and the NIHR. The Reasons for Geographic and Racial Differences in Stroke (REGARDS) Study is supported by a cooperative agreement U01 NS041588 from the National Institute of Neurological Disorders and Stroke, National Institutes of Health, U.S. Department of Health and Human Services.  REGARDS genetics was funded by R01HL136666. A full list of participating REGARDS investigators and institutions can be found at http://www.regardsstudy.org. The Secondary Prevention of Small Subcortical Strokes trial was funded by the US National Institute of Health and Neurological Disorders and Stroke grant No. U01NS38529-04A1 (principal investigator, Oscar R. Benavente; coprincipal investigator, Robert G. Hart). The SPS3 Genetic Substudy (SPS3-GENES) was funded by R01 NS073346 (coprincipal investigators, Julie A. Johnson, Oscar R. Benavente, and Alan R. Shuldiner) and U01 GM074492-05S109 (principal investigator, Julie A. Johnson). The principal funding for this study was provided by the Wellcome Trust, as part of the Wellcome Trust Case Control Consortium 2 project (085475/B/08/Z and 085475/Z/08/Z and WT084724MA). Collection of some of the St George’s stroke cohort was supported by project grant support from the Stroke Association. Hugh Markus is supported by an NIHR Investigator award. Matthew Traylor was supported by project grant funding from the Stroke Association.(TSA 2013/01). The GWAS component of the VISP study was supported by the United States National Human Genome Research Institute (NHGRI), Grant U01 HG005160 (PI Michèle Sale & Bradford Worrall), as part of the Genomics and Randomized Trials Network (GARNET). Genotyping services were provided by the Johns Hopkins University Center for Inherited Disease Research (CIDR), which is fully funded through a federal contract from the NIH to the Johns Hopkins University. Assistance with data cleaning was provided by the GARNET Coordinating Center (U01 HG005157; PI Bruce S Weir). Study recruitment and collection of datasets for the VISP clinical trial were supported by an investigator-initiated research grant (R01 NS34447; PI James Toole) from the United States Public Health Service, NINDS, Bethesda, Maryland. Control data for comparison with European ancestry VISP stroke cases were obtained through the database of genotypes and phenotypes (dbGAP) High Density SNP Association Analysis of Melanoma: Case-Control and Outcomes Investigation (phs000187.v1.p1; R01CA100264, 3P50CA093459, 5P50CA097007, 5R01ES011740, 5R01CA133996, HHSN268200782096C; PIs Christopher Amos, Qingyi Wei, Jeffrey E. Lee). For VISP stroke cases of African ancestry, a subset of the Healthy Aging in Neighborhoods of Diversity across the Life Span study (HANDLS) were used as stroke free controls. HANDLS is funded by the National Institute of Aging (1Z01AG000513; PI Michele K. Evans). The Women’s Health Initiatives (WHI) program was funded by the National Heart, Lung, and Blood Institute, NIH, US Department of Health and Human Services through contracts N01WH22110, 24152, 32100-2, 32105-6, 32108-9, 32111-13, 32115, 32118 to 32119, 32122, 42107-26, 42129-32, and 44221. The Hormones and Biomarkers Predicting Stroke (HaBPS) was supported by a grant from the National Institutes of Neurological Disorders and Stroke (R01NS042618).The Washington University St. Louis Stroke Study (WUSTL): The collection, extraction of DNA from blood, and storage of specimens were supported by 2 NINDS NIH grants (P50 NS055977 and R01 NS8541901). Basic demographic and clinical characterization of stroke phenotype was prospectively collected in the Cognitive Rehabilitation and Recovery Group (CRRG) registry. The Recovery Genomics after Ischemic Stroke (ReGenesIS) study was supported by a grant from the Barnes-Jewish Hospital Foundation.

**CHARGE**

Infrastructure for the CHARGE Consortium is supported in part by the National Heart, Lung, and Blood Institute grant R01HL105756; the NeuroCHARGE working group is supported in part by National Institute on Aging grant R01AG033193, andpartially funded by AG033193, AG049505, AG052409, AG059421.

*AGES***:** Age, Gene/Environment Susceptibility (AGES) -Reykjavik This study has been funded by National Institutes of Health-contract N01- AG-1-2100, the National Institute on Aging Intramural Research Program, Hjartavernd, and the Icelandic Parliament.

*ARIC***:** The Atherosclerosis Risk in Communities study has been funded in whole or in part with Federal funds from the National Heart, Lung, and Blood Institute, National Institutes of Health, Department of Health and Human Services (contract numbers HHSN268201700001I, HHSN268201700002I, HHSN268201700003I, HHSN268201700004I and HHSN268201700005I), R01HL087641, R01HL059367 and R01HL086694; National Human Genome Research Institute contract U01HG004402; and National Institutes of Health contract HHSN268200625226C. The authors thank the staff and participants of the ARIC study for their important contributions. Infrastructure was partly supported by Grant Number UL1RR025005, a component of the National Institutes of Health and NIH Roadmap for Medical Research.This project was also partially supported by NIH R01 grant NS087541 to MF.

*CHS***:** Cardiovascular Health Study: This CHS research was supported by NHLBI contracts HHSN268201200036C, HHSN268200800007C, HHSN268201800001C, N01HC55222, N01HC85079, N01HC85080, N01HC85081, N01HC85082, N01HC85083, N01HC85086, 75N92021D00006; and NHLBI grants U01HL080295, R01HL087652, R01HL105756, R01HL103612, R01HL120393, R01HL085251, and U01HL130114 with additional contribution from the National Institute of Neurological Disorders and Stroke (NINDS). Additional support was provided through R01AG023629 from the National Institute on Aging (NIA). A full list of principal CHS investigators and institutions can be found at CHS-NHLBI.org.

The provision of genotyping data was supported in part by the National Center for Advancing Translational Sciences, CTSI grant UL1TR001881, and the National Institute of Diabetes and Digestive and Kidney Disease Diabetes Research Center (DRC) grant DK063491 to the Southern California Diabetes Endocrinology Research Center.

The content is solely the responsibility of the authors and does not necessarily represent the official views of the National Institutes of Health.

*FHS:* R01 NS017950 (PI Seshadri), contract with NHLBI: HHSN 268201500001I, 75N92019D00031, N01-HC-25195, R01 AG054076 and AG049607; Funding for SHARe Affymetrix genotyping was provided by NHLBI Contract N02-HL-64278. The computational work reported in this paper was performed on the Shared Computing Cluster which is administered by Boston University’s Research Computing Services. We thank the staff and participants of the Framingham Study.

*FINRISK:* TJN was Funded by the Emil Aaltonen Foundation, the Finnish Foundation for Cardiovascular Research, and the Academy of Finland (grant n:o 321351). ASH was funded by the Academy of Finland (grant no.: 321356).

*HEALTH ABC:* The Health ABC Study was supported by National Institute on Aging (NIA) Contracts N01-AG-6-2101; N01-AG-6-2103; N01-AG-6-2106; NIA grant R01-AG028050, and NINR grant R01-NR012459. This research was funded in part by the Intramural Research Program of the NIH, National Institute on Aging.

*Rotterdam Study:* The generation and management of GWAS genotype data for the Rotterdam Study is supported by the Netherlands Organisation of Scientific Research NWO Investments (nr. 175.010.2005.011, 911-03-012). This study is funded by the Research Institute for Diseases in the Elderly (014-93-015; RIDE2), the Netherlands Genomics Initiative (NGI)/Netherlands Organisation for Scientific Research (NWO) project nr. 050-060-810. The Rotterdam Study is funded by Erasmus Medical Center and Erasmus University, Rotterdam, Netherlands Organization for the Health Research and Development (ZonMw), the Research Institute for Diseases in the Elderly (RIDE), the Ministry of Education, Culture and Science, the Ministry for Health, Welfare and Sports, the European Commission (DG XII), and the Municipality of Rotterdam. MAI is supported by an NWO Veni grant (916.13.054).

*SHIP:* SHIP is part of the Community Medicine Research net of the University of Greifswald, Germany, which is funded by the Federal Ministry of Education and Research (grants no. 01ZZ9603, 01ZZ0103, and 01ZZ0403), the Ministry of Cultural Affairs as well as the Social Ministry of the Federal State of Mecklenburg-West Pomerania, and the network ‘Greifswald Approach to Individualized Medicine (GANI_MED)’ funded by the Federal Ministry of Education and Research (grant 03IS2061A). Genome-wide data have been supported by the Federal Ministry of Education and Research (grant no. 03ZIK012) and a joint grant from Siemens Healthineers, Erlangen, Germany and the Federal State of Mecklenburg- West Pomerania. The University of Greifswald is a member of the Caché Campus program of the InterSystems GmbH.

*WGHS:* The WGHS is supported by the National Heart, Lung, and Blood Institute (HL043851, HL080467, and HL099355) and by the National Cancer Institute (CA047988 and UM1CA182913) with funding for genotyping provided by Amgen.

*MESA:* MESA and the MESA SHARe project are conducted and supported by the National Heart, Lung, and Blood Institute (NHLBI) in collaboration with MESA investigators. Support for MESA is provided by contracts 75N92020D00001, HHSN268201500003I, N01-HC-95159, 75N92020D00005, N01-HC-95160, 75N92020D00002, N01-HC-95161, 75N92020D00003, N01-HC-95162, 75N92020D00006, N01-HC-95163, 75N92020D00004, N01-HC-95164, 75N92020D00007, N01-HC-95165, N01-HC-95166, N01-HC-95167, N01-HC-95168, N01-HC-95169, UL1-TR-000040, UL1-TR-001079, UL1-TR-001420, UL1-TR-001881, and DK063491.

*PROSPER:* The PROSPER study was supported by an investigator initiated grant obtained from Bristol-Myers Squibb. Prof. Dr. J. W. Jukema is an Established Clinical Investigator of the Netherlands Heart Foundation (grant 2001 D 032). Support for genotyping was provided by the seventh framework program of the European commission (grant 223004) and by the Netherlands Genomics Initiative (Netherlands Consortium for Healthy Aging grant 050-060-810).

*3C:* The Three City (3C) Study is conducted under a partnership agreement among the Institut National de la Santé et de la Recherche Médicale (INSERM), the University of Bordeaux, and Sanofi-Aventis. The Fondation pour la Recherche Médicale funded the preparation and initiation of the study. The 3C Study is also supported by the Caisse Nationale Maladie des Travailleurs Salariés, Direction Générale de la Santé, Mutuelle Générale de l’Education Nationale (MGEN), Institut de la Longévité, Conseils Régionaux of Aquitaine and Bourgogne, Fondation de France, and Ministry of Research–INSERM Programme “Cohortes et collections de données biologiques.” Christophe Tzourio and Stéphanie Debette have received investigator-initiated research funding from the French National Research Agency (ANR) and from the Fondation Leducq. We thank Dr. Anne Boland (CNG) for her technical help in preparing the DNA samples for analyses. This work was supported by the National Foundation for Alzheimer’s disease and related disorders, the Institut Pasteur de Lille, the labex DISTALZ and the Centre National de Génotypage. Quentin Le Grand benefited from the Digital Public Health Graduate Program (DPH), a PhD program supported by the French Investment for the Future Programme (17-EURE-0019).

**EPIC-CVD:**

EPIC was funded by the UK Medical Research Council (G0800270), British Heart Foundation (SP/09/002), UK National Institute for Health Research Cambridge Biomedical Research Centre, European Research Council (268834), European Commission Framework Programme 7 (HEALTH-F2-2012-279233).

**VHIR-FMT-Barcelona:**

The Barcelona GWAs Study was supported by the Genetic contribution to functional Outcome and Disability after Stroke (GODS) project, Fundació la Marató de TV3 and by the Miguel Servet grant (Pharmastroke project: CP12/03298). Neurovascular research Laboratory takes part in the INVICTUS network. I. F-C. is supported by the Miguel Servet programme (CP12/03298), Instituto de Salud Carlos III.

**Biobank Japan:**

This study was funded by the BioBank Japan project, which is supported by the Ministry of Education, Culture, Sports, Sciences and Technology (MEXT) of Japanese government and the Japan Agency for Medical Research and Development (AMED, grant ID JP21km0605001).

**CADISP**:

The Cervical Artery Dissections and Ischemic Stroke Patients (CADISP) study has been supported by Inserm, Lille 2 University, Institut Pasteur de Lille and Lille University Hospital and received funding from the ERDF (FEDER funds) and Région Nord-Pas de Calais in the frame of Contrat de Projets Etat-Region 2007-2013 Région Nord-Pas-de-Calais - Grant N°09120030, Centre National de Genotypage, Emil Aaltonen Foundation, Paavo Ilmari Ahvenainen Foundation, Helsinki University Central Hospital Research Fund, Helsinki University Medical Foundation, Päivikki and Sakari Sohlberg Foundation, Aarne Koskelo Foundation, Maire Taponen Foundation, Aarne and Aili Turunen Foundation, Lilly Foundation, Alfred Kordelin Foundation, Finnish Medical Foundation, Orion Farmos Research Foundation, Maud Kuistila Foundation, the Finnish Brain Foundation, Biomedicum Helsinki Foundation, Projet Hospitalier de Recherche Clinique Régional, Fondation de France, Génopôle de Lille, Adrinord, Basel Stroke-Funds, Käthe-Zingg-Schwichtenberg-Fonds of the Swiss Academy of Medical Sciences, Swiss Heart Foundation.

**COMPASS:**

The authors thank the staff and participants of the ARIC, CHS, VISP, HANDLS, INTERSTROKE, ISGS, JHS, SIGNET-REGARDS, GEOS, SiGN, SLESS, SWISS, and WHI studies for their dedication, and willingness to participate in the respective research studies, which made this work possible.

ARIC Study (Atherosclerosis Risk in Communities): supported in whole or in part with Federal funds from the NHLBI (National Heart, Lung, and Blood Institute), National Institutes of Health, Department of Health and Human Services (contract numbers HHSN268201700001I, HHSN268201700002I, HHSN268201700003I, HHSN268201700004I, and HHSN268201700005I), R01HL087641, R01HL059367 and R01HL086694; National Human Genome Research Institute contract U01HG004402; and National Institutes of Health contract HHSN268200625226C. We thank the staff and participants of the ARIC study for their important contributions. Infrastructure was partly supported by Grant Number UL1RR025005, a component of the National Institutes of Health and National Institutes of Health (NIH) Roadmap for Medical Research. CHS (Cardiovascular Health Study): supported by NHLBI contracts HHSN268201200036C, HHSN268200800007C, N01HC55222, N01HC85079, N01HC85080, N01HC85081, N01HC85082, N01HC85083, N01HC85086; and grants U01HL080295, R01HL087652, R01HL105756, R01HL103612, and R01HL120393 with contributions from the National Institute of Neurological Disorders and Stroke. Additional support was provided through R01AG023629 from the National Institute on Aging. A full list of principal CHS investigators and institutions can be found at [CHS-NHLBI.org](https://www.ahajournals.org/doi/10.1161/CHS-NHLBI.org). The provision of genotyping data was supported in part by the National Center for Advancing Translational Sciences, grant UL1TR000124, and the National Institute of Diabetes and Digestive and Kidney Disease Research Center grant DK063491 to the Southern California Diabetes Endocrinology Research Center. VISP (Vitamin Intervention for Stroke Prevention): funded by the National Institute of Neurological Disorders and Stroke (R01-NS34447). Genome-wide association study (GWAS) data for a subset of VISP participants were supported by the National Human Genome Research Institute (U01-HG005160), as part of the GARNET (Genomics and Randomized Trials Network; Principal Investigator [PI]: Drs Sale and Worrall). INTERSTROKE has received unrestricted grants from the Canadian Institutes of Health Research, Heart and Stroke Foundation of Canada, Canadian Stroke Network, Pfizer Cardiovascular Award, Merck, AstraZeneca, and Boehringer Ingelheim. JHS (Jackson Heart Study): supported and conducted in collaboration with Jackson State University (HHSN268201800013I), Tougaloo College (HHSN268201800014I), the Mississippi State Department of Health (HHSN268201800015I) and the University of Mississippi Medical Center (HHSN268201800010I, HHSN268201800011I, and HHSN268201800012I) contracts from the NHLBI and the National Institute for Minority Health and Health Disparities (NIMHD). SIGNET-REGARDS (Sea Islands Genetics Network–Reasons for Geographic and Racial Differences in Stroke: SIGNET was supported by R01 DK084350 (Dr Sale) and consists of data from the REGARDS cohort, (U01 NS041588; G Howard). GEOS Study (The Genetics of Early Onset Stroke): GEO Study was supported by the NIH Genes, Environment, and Health Initiative grant U01 HG004436, as part of the Gene Environment Association Studies consortium, with additional support provided by the Mid-Atlantic Nutrition and Obesity Research Center (P30 DK072488) and the Office of Research and Development, Medical Research Service, and the Baltimore Geriatrics Research, Education, and Clinical Center of the Department of Veterans Affairs. Genotyping services were provided by the Johns Hopkins University Center for Inherited Disease Research (CIDR), which is fully funded through a federal contract from the NIH to the Johns Hopkins University (contract No. HHSN268200782096C). Assistance with data cleaning was provided by the Gene Environment Association Studies Consortium Coordinating Center (U01 HG 004446; PI Bruce S. Weir). Study recruitment and assembly of data sets were supported by a Cooperative Agreement with the Division of Adult and Community Health, Centers for Disease Control and by grants from the National Institute of Neurological Disorders and Stroke (NINDS) and the NIH Office of Research on Women’s Health (R01 NS45012, U01 NS069208-01).

NINDS-SiGN Groups 4: The SiGN study was funded by a cooperative agreement grant from the NINDS U01 NS069208. Genotyping services were provided by the Johns Hopkins University CIDR, which is fully funded through a federal contract from the NIH to the Johns Hopkins University (contract No.HHSN268200782096C). The Biostatistics Department Genetics Coordinating Center at the University of Washington (Seattle) provided more extensive quality control of the genotype data through a subcontract with CIDR. Additional support to the Administrative Core of SiGN was provided by the Dean’s Office, University of Maryland School of Medicine.

SLESS (South London Ethnicity and Stroke Study). Recruitment to SLESS was supported by a program grant from the Stroke Association. This study represents independent research part-funded by the National Institute for Health Research Biomedical Research Centre at South London and Maudsley National Health Service (NHS) Foundation Trust and King’s College London, and the National Institute for Health Research Biomedical Research Centre at Guy’s and St Thomas’ NHS Foundation Trust and King’s College London. Hugh Markus is supported by a National Institute for Health Research (NIHR) Senior Investigator Award, and his work is supported by NIHR Comprehensive Biomedical Research Unit funding awarded to Cambridge University Hospitals Trust. ISGS (Ischemic Stroke Genetics Study) and SWISS (Siblings with Ischemic Stroke Study): ISGS and SWISS were supported by the National Institute of Neurological Disorders and Stroke grants (R01 NS42733; Dr Meschia) and (R01NS39987; Dr Meschia), respectively. Both studies received additional support, in part, from the Intramural Research Program of the National Institute of Aging (Z01 AG000954-06; PI Singleton), and used samples and clinical data from the NIH-NINDS Human Genetics Resource Center DNA and Cell Line Repository (<http://ccr.cori-ell.org/ninds>), human subjects protocol numbers 2003–081 and 2004–147. WHI (Women’s Health Initiative): supported by the NHLBI, NIH, and the US Department of Health and Human Services through contracts N01WH22110, 24152, 32100-2, 32105-6, 32108-9, 32111-13, 32115, 32118-32119, 32122, 42107-26, 42129-32, and 44221. The funders had no role in study design, data collection and analysis, decision to publish, or manuscript preparation. Dr Hyacinth is supported by NIH/NHLBI grants U01HL117721, R01HL138423, and R56HL136210. HANDLS (Healthy Aging in Neighborhoods of Diversity Across the Life Span): supported by the Intramural Research Program of the NIH, National Institute of Aging (project no. Z01-AG000513 and human subjects protocol no. 09AGN248).

**Glasgow Stroke Sample:**

The work was supported by NHS Greater Glasgow Endowment funds.

**Hisayama and Hisayama-FSR study:**

We thank Prof. Takanari Kitazono and Prof. Masahiro Kamouchi (Graduate School of Medical Sciences, Kyushu University, Fukuoka, Japan) for collecting clinical samples.

**HVH 1 & 2:**

The research of the Heart and Vascular Health Studies has been funded in part by NHLBI grants R01HL085251 and R01HL073410.

**INTERSTROKE:** We would like to acknowledge all of the investigators and participants of the INTERSTROKE study.

**MDC:** The Malmӧ Diet and Cancer Study was supported by the Swedish Research Council (Vetenskapsrådet), Heart and Lung Foundation (Hjärt och Lungfonden), and Swedish Stroke Foundation (Strokeförbundet).

**RACE:** Fieldwork in RACE was funded by the R-21 grant provided by the NINDS and the Fogarty International Center (1R21NS064908-01) and educational grants available to Dr. Saleheen at the

Center for Non-Communicable Diseases, Pakistan. We would also like to acknowledge the contributions made by Professor John Danesh, Dr. Ayeesha Kamal and Professor Panos Deloukas.

**SAHLSIS:** The Sahlgrenska Academy Study of Ischemic Stroke was supported by the Swedish Research Council (2021-01114), the Swedish Heart and Lung Foundation (20190203), the Swedish state under the agreement between the Swedish government and the county councils, the ALF agreement (ALFGBG-720081), the Swedish Stroke Association, the Rune and Ulla Amlöv Foundation for Neurologic Research, the John and Brit Wennerström Foundation for Neurologic Research, the Per-Olof Ahl Foundation for Neurological Research. and the Gothenburg Foundation for Neurological Research. The authors thank research nurse Ingrid Eriksson for her excellent work and assistance in recruiting the study participants and for conducting the follow-up study. Furthermore, we are grateful to our study participants without whom this work would not have been possible.

**SIFAP:** The sifap study (Stroke In Young Fabry Patients, http://www.sifap.eu; ClinicalTrials.gov: NCT00414583) has been supported partially by an unrestricted scientific grant from Shire Human Genetic Therapies. Funding for genotyping and analysis of samples were supported by the National Institutes of Health Genes, Environment and Health Initiative (GEI) Grant U01 HG004436, as part of the GENEVA consortium.

**UK - young lacunar stroke DNA resource:** Collection of the UK Young Lacunar Stroke DNA Study (DNA Lacunar) was primarily supported by the Wellcome Trust (WT072952) with additional support from the Stroke Association (TSA 2010/01). Additional sample collection and data analysis were supported by a BHF programme grant RG/16/4/32218. Dr Markus is supported by the National Institute for Health Research Cambridge University Hospitals Comprehensive Biomedical Research Centre and a National Institute for Health Research Senior Investigator award. Hugh Markus and Steven Bell’s research is supported by infrastructural support from the Cambridge BHF Centre of Research Excellence [RE/18/1/34212] and the NIHR Cambridge Biomedical Research Centre (BRC-1215-20014).

**ICH**

Funding provided as follows: GERFHS, NIH grants NS36695 and NS30678; GOCHA, NIH grant R01NS059727, the Keane Stroke Genetics Research Fund, the Edward and Maybeth Sonn Research Fund, and the University of Michigan General Clinical Research Center M01 RR000042; ERICH, NIH grant NS069763; HM-ICH, Instituto de Salud Carlos III with the grants “Registro BASICMAR” Funding for Research in Health (PI051737), “GWALA project” from Fondos de Investigación Sanitaria ISC III (PI10/02064), and Fondos FEDER/EDRF Red de Investigación Cardiovascular (RD12/0042); JUHSS, Polish Ministry of Education grant N402 083934; LSR, Lund University, Region Skåne, the Swedish Research Council (K2010-61X-20378-04-3), the Swedish Stroke Association, the Freemasons Lodge of Instruction EOS in Lund, and the King Gustaf V and Queen Victoria’s foundations; G.J.F. and H.B.B., NIH SPOTRIAS fellowship P50NS061343; C.D.A., NIH grants R01NS103924, R01NS069763, American Heart Association-Bugher Foundation Centers for Excellence in Hemorrhagic Stroke.

**GIGASTROKE new studies**

**Epidemiological Prevention Study of Zoetermeer (EPOZ)**

We are grateful to the contributions of the EPOZ study participants. We would like to thank dr. ir. Natalie Terzikhan for imputing the genetic data

**FinnGen study**

We want to acknowledge the participants and investigators of FinnGen study. The FinnGen project is funded by two grants from Business Finland (HUS 4685/31/2016 and UH 4386/31/2016) and the following industry partners: AbbVie Inc., AstraZeneca UK Ltd, Biogen MA Inc., Bristol Myers Squibb (and Celgene Corporation & Celgene International II Sàrl), Genentech Inc., Merck Sharp & Dohme LCC, Pfizer Inc., GlaxoSmithKline Intellectual Property Development Ltd., Sanofi US Services Inc., Maze Therapeutics Inc., Janssen Biotech Inc, Novartis AG, and Boehringer Ingelheim International GmbH. Following biobanks are acknowledged for delivering biobank samples to FinnGen: Auria Biobank (www.auria.fi/biopankki), THL Biobank (www.thl.fi/biobank), Helsinki Biobank (www.helsinginbiopankki.fi), Biobank Borealis of Northern Finland (<https://www.ppshp.fi/Tutkimus-ja-opetus/Biopankki/Pages/Biobank-Borealis-briefly-in-English.aspx>), Finnish Clinical Biobank Tampere (www.tays.fi/en-US/Research_and_development/Finnish_Clinical_Biobank_Tampere), Biobank of Eastern Finland (www.ita-suomenbiopankki.fi/en), Central Finland Biobank (www.ksshp.fi/fi-FI/Potilaalle/Biopankki), Finnish Red Cross Blood Service Biobank ([www.veripalvelu.fi/verenluovutus/biopankkitoiminta](http://www.veripalvelu.fi/verenluovutus/biopankkitoiminta)), Terveystalo Biobank ([www.terveystalo.com/fi/Yritystietoa/Terveystalo-Biopankki/Biopankki/](http://www.terveystalo.com/fi/Yritystietoa/Terveystalo-Biopankki/Biopankki/)) and Arctic Biobank (<https://www.oulu.fi/en/university/faculties-and-units/faculty-medicine/northern-finland-birth-cohorts-and-arctic-biobank>). All Finnish Biobanks are members of BBMRI.fi infrastructure ([www.bbmri.fi](http://www.bbmri.fi/)). Finnish Biobank Cooperative -FINBB (<https://finbb.fi/>) is the coordinator of BBMRI-ERIC operations in Finland. The Finnish biobank data can be accessed through the Fingenious^®^ services (<https://site.fingenious.fi/en/>) managed by FINBB.

**The Trøndelag Health Study (HUNT)**

The Trøndelag Health Study (HUNT) is a collaboration between HUNT Research Centre (Faculty of Medicine and Health Sciences, Norwegian University of Science and Technology NTNU), Trøndelag County Council, Central Norway Regional Health Authority, and the Norwegian Institute of Public Health. The genotyping was financed by the National Institute of health (NIH), University of Michigan, The Norwegian Research council, and Central Norway Regional Health Authority and the Faculty of Medicine and Health Sciences, Norwegian University of Science and Technology (NTNU). The genotype quality control and imputation has been conducted by the K.G. Jebsen center for genetic epidemiology, Department of public health and nursing, Faculty of medicine and health sciences, Norwegian University of Science and Technology (NTNU).

**UK BIOBANK**

This research has been conducted using the UK Biobank Resource under Application Number 2532

**Estonian Biobank (EstBB) and Estonian Young Stroke Registry (UTARTU-EstBB)**

EstBB thanks all participants and staff of the Estonian biobank for their contribution to this research and the analytical work of EstBB was carried out in part in the High Performance Computing Center of the University of Tartu. We acknowledge the work of the Estonian Biobank Research Team: Andres Metspalu, Mari Nelis, Reedik Mägi and Tõnu Esko. This research at EstBB was supported by the Estonian Research Council grant PUT (PRG184) and the European Union through the European Regional Development Fund (Project No. 2014-2020.4.01.15-0012), the PRECISE4Q project has received funding from the European Union’s Horizon 2020 Research and Innovation Programme under Grant agreement 777107.

**Geisinger Ischemic Stroke cohort in MyCode Biobank (GEISINGER)**

We thank the participants of the MyCode Community Health Initiative for the use of their genomic and electronic health information. Enrollment of MyCode participants and exome sequencing was supported in part by the Regeneron Genetics Center. We thank the Geisinger-Regeneron DiscovEHR collaboration for making the genotype and phenotype data available for this project.

**Copenhagen City Heart Study (CCHS)**

The CCHS is funded by the Danish Heart Foundation and private foundations. We thank staff and participants in the Copenhagen City Heart Study for their important contributions.

**Danish Twin Registry**

DTR is supported by grants from The National Program for Research Infrastructure 2007 from the Danish Agency for Science, Technology and Innovation (09-063256) and the US National Institutes of Health (P01 AG08761). Genotyping was supported by NIH R01 AG037985 (Pedersen).

**SMART**

Netherlands CardioVascular Research Initiative of the Netherlands Heart Foundation (CVON 2011/B019 and CVON 2017-20: Generating the best evidence-based pharmaceutical targets for atherosclerosis [GENIUS I&II]). We are thankful for the support of the ERA-CVD program ‘druggable-MI-targets’ (grant number: 01KL1802), the EU H2020 TO_AITION (grant number: 848146), and the Leducq Fondation ‘PlaqOmics’.

**ASPirin in Reducing Events in the Elderly (ASPREE)**

The ASPREE study and Healthy Ageing Biobank was supported by an ASPREE Flagship cluster grant (including the Commonwealth Scientific and Industrial Research Organisation, Monash University, Menzies Research Institute, Australian National University, University of Melbourne); and grants (U01AG029824 and U19AG062682) from the National Institute on Aging and the National Cancer Institute at the National Institutes of Health, by grants (334047 and 1127060) from the National Health and Medical Research Council of Australia, and by Monash University and the Victorian Cancer Agency. Paul Lacaze is supported by a National Heart Foundation Future Leader Fellowship (ID 102604).

**German stroke GWAS**

*PROSCIS-B:*

PROSCIS-B study was funded by the Federal Ministry of Education and Research via the grant Center for Stroke Research Berlin (01 EO 0801).; M.E. received funding from DFG under Germany´s Excellence Strategy – EXC-2049 – 390688087, BMBF, DZNE, DZHK, EU, Corona Foundation, and Fondation Leducq.

*SICFAIL:*

The SICFAIL study was supported by the German Ministry of Research and Education within the Comprehensive Heart Failure Centre Würzburg (grant numbers BMBF 01EO1004 and01EO1504).

*MONICA/KORA Augsburg Study:*

The KORA study was initiated and financed by the Helmholtz Zentrum München – German Research Center for Environmental Health, which is funded by the German Federal Ministry of Education and Research (BMBF) and by the State of Bavaria. Furthermore, KORA research was supported within the Munich Center of Health Sciences (MC-Health), Ludwig-Maximilians-Universität, as part of LMUinnovativ. Funded by the Bavarian State Ministry of Health and Care through the research project DigiMed Bayern (www.digimed-bayern.de). Supported by the DZHK (German Centre for Cardiovascular Research) and by the BMBF (German Ministry of Education and Research).

**Dutch Stroke cohorts**

*FUTURE Study – Radboudumc, Nijmegen, the Netherlands*

AMT has received a grant from the Junior Staff Member Dutch Heart Foundation (2016T044). FE de Leeuw has received a VIDI grant (016-126-351), the Clinical established investigator Dutch Heart Foundation grant (2014 T060) and funding from Bike4Brains.

*The Dutch Parelsnoer initiative (PSI) Cerebrovascular Disease Study*

PSI-CVA was co-financed by the Dutch Government, the Dutch Federation of University Medical Centers and the eight participating UMC’s (from 2007-2011). The continuation of the PSI is financed by the UMC’s. Y.M. Ruigrok received funding from the European Research Council (ERC) under the European Union's Horizon 2020 research and innovation program (grant agreement No. 852173).

**China Kadoorie Biobank (CKB)**

The most important acknowledgement is to the participants in the study and the members of the survey teams in each of the 10 regional centres, and to the project development and management teams based at Beijing, Oxford and the 10 regional centres. China’s National Health Insurance provides electronic linkage to all hospital treatments.

The CKB baseline survey and the first re-survey were supported by the Kadoorie Charitable Foundation in Hong Kong. Long-term follow-up was supported by the Wellcome Trust (212946/Z/18/Z, 202922/Z/16/Z, 104085/Z/14/Z, 088158/Z/09/Z), the National Key Research and Development Program of China (2016YFC0900500, 2016YFC0900501, 2016YFC0900504, 2016YFC1303904), and the National Natural Science Foundation of China (91843302). DNA extraction and genotyping was funded by GlaxoSmithKline, and the UK Medical Research Council (MC-PC-13049, MC-PC-14135). The project is supported by core funding from the UK Medical Research Council (MC_UU_00017/1,MC_UU_12026/2, MC_U137686851), Cancer Research UK (C16077/A29186; C500/A16896), and the British Heart Foundation (CH/1996001/9454) to the Clinical Trial Service Unit and Epidemiological Studies Unit and to the MRC Population Health Research Unit at Oxford University.

**The Korean Cancer Prevention Study-II (KCPS2) Biobank**

The authors wish to thank the Korean Central Cancer Registry and the National Insurance Data Service for their assistance in disease and mortality data linkage. Moreover, we would also like to thank the study participants who agreed to provide their data.

**JSB BRAZIL**

The JSB was supported by the Research Support Fund of the University of Region of Joinville. The genotyping of the samples was supported by National Council for Scientific and Technological Development (CNPq Project Grant: 402396/2013-8). The authors are grateful to the Joinville Stroke Registry Team by recruitment and sample collection, particularly Vivian Nagel, the JSB Laboratory staff and, all participants included in the JSB. Also, special thanks to Professor Dr. Norberto Cabral (in memorian) for his encouragement and initiative of JSB conception.

**GENERACION Project.**

This study has been funded by Carlos III Institute PI15/01978, PI17/02089, PI18/01338, PI20/00678, and RETICS INVICTUS PLUS RD16/0019), by Marató TV3 support of the Epigenesis study by the Fundació Docència i Recerca FMT grant for the Epigenesis project, by Eranet-Neuron of the Ibiostroke project (AC19/00106), NIH R01NS085419, Barnes-Jewish Hospital Foundation and by Boehringer Ingelheim of the SEDMAN Study. This study uses data generated by the GCAT | Genomes for Life. Cohort study of the Genomes of Catalonia, IGTP (GCAT Cession Research Project PI-2018-01). A full list of the investigators who contributed to the generation of the data is available from http://www.genomesforlife.com/.

**AIIMS-DELHI (Indian Stroke GWAS)**

The present study was funded by the Department of Biotechnology, Ministry of Science and Technology, Government of India (grant BT/01/COE/06/02/09-II). The funding support for sample collection from South India and East India was provided by the UK-India Education Research Initiative. Ganesh Chauhan is supported by DBT-Ramalingaswami Re-Entry Fellowship. The UK Biobank datasets used in this study were obtained under application No. 37489.

**Tohoku Medical MEGABANK**

Tohoku Medical MEGABANK (TMM) has been supported in part by MEXT-JST and AMED; most recent grant numbers are JP20km0105001, JP20km0105002, JP20km0105003 and JP20km0105004

**Multi-tissue pQTL resource**

This work was supported by grants from the National Institutes of Health (R01AG044546 (Carlos Cruchaga)), P01AG003991(CC, JCM), RF1AG053303 (CC), RF1AG058501 (CC), U01AG058922 (CC)), and the Chuck Zuckerberg Initiative (CZI). This work was supported by access to equipment made possible by the Hope Center for Neurological Disorders, the Neurogenomics and Informatics Center (NGI: https://neurogenomics.wustl.edu/)and the Departments of Neurology and Psychiatry at Washington University School of Medicine.

**Other acknowledgements:**

M.I. is supported by the Munz Chair of Cardiovascular Prediction and Prevention and the NIHR Cambridge Biomedical Research Centre (BRC-1215-20014) [*]. *The views expressed are those of the author(s) and not necessarily those of the NIHR or the Department of Health and Social Care.

S.N. was supported by Takeda Science Foundation. Y.O. was supported by JSPS KAKENHI (19H01021, 20K21834), and AMED (JP21km0405211, JP21ek0109413, JP21ek0410075, JP21gm4010006, and JP21km0405217), JST Moonshot R&D (JPMJMS2021, JPMJMS2024), Takeda Science Foundation, and Bioinformatics Initiative of Osaka University Graduate School of Medicine, Osaka University.

N.H is a Goren Khazzam senior lecturer in neuroscience, supported by Myers Foundation (N.H.), Israel Science Foundation (ISF) research grants no. 1709/19 (N.H.), The European Research Council grant 853409 (N.H.).

S.D. is supported by a grant overseen by the French National Research Agency (ANR) as part of the “Investment for the Future Programme” ANR-18-RHUS-0002, by European Union’s Horizon 2020 research and innovation programme under grant agreement No 640643 and 754517

Computations were performed on the Bordeaux Bioinformatics Center (CBiB) computer resources, University of Bordeaux. Funding support for additional computer resources has been provided to S.D. by the Fondation Claude Pompidou.

**GIGASTROKE follow-up studies**

**Million Veteran Program**

This research is based on data from the Million Veteran Program and is supported by funding from the Department of Veterans Affairs Office of Research and Development, Million Veteran Program Awards I01-BX004821(PIs: Wilson/Cho). We are grateful to the Million Veteran Program participants and staff (see supporting information for full acknowledgement). The views and opinions expressed in this manuscript do not represent those of the Department of Veterans Affairs, the National Institutes of Health, or the United States Government.

**Penn Medicine Biobank**

We acknowledge the Penn Medicine BioBank (PMBB) for providing data and thank the patient-participants of Penn Medicine who consented to participate in this research program. We would also like to thank the Penn Medicine BioBank team and Regeneron Genetics Center for providing genetic variant data for analysis.  The PMBB is approved under IRB protocol# 813913 and supported by Perelman School of Medicine at University of Pennsylvania, a gift from the Smilow family, and the National Center for Advancing Translational Sciences of the National Institutes of Health under CTSA award number UL1TR001878. SMD is supported by the US Departemnt of Veterans Affairs Award IK2-CX001780. This publication does not represent the views of the Department of Veterans Affairs or the United States Government.

**Mass General Brigham Biobank**

We thank Mass General Brigham Biobank for providing samples, genomic data, and health information data.

**Stroke Investigative Research and Educational Network (SIREN)**

The National Institutes of Health grants supported the study and investigators as follows: SIREN (U54HG007479), SIBS Genomics (R01NS107900), SIBS Gen Gen (R01NS107900‐02S1), ARISES (R01NS115944‐01), H3Africa CVD Supplement (3U24HG009780‐03S5), CaNVAS (1R01NS114045-01), Sub-Saharan Africa Conference on Stroke (SSACS) 1R13NS115395-01A1 and Training Africans to Lead and Execute Neurological Trials & Studies (TALENTS) D43TW012030.

**Helsinki Ischemic Stroke Genetics Study**

The study was supported by the Finnish Medical Foundation and the Helsinki University Central Hospital governmental subsidiary funds for clinical research. We thank Marja Metso, RN for her long-lasting support of the study. The data used for the research was imputed with the SISu v3 Imputation reference panel. We thank the Sequencing Informatics Team, FIMM Human Genomics, University of Helsinki for the work done in preparation of the reference panel data. We thank all study participants for their generous participation.

**Taiwan Biobank**

In the follow-up study and polygenic score analyses summary statistics and data derived from Taiwan Biobank and the Taiwanese National Health Insurance Research Database were used. We thank "Taiwan Biobank", the "Health and Welfare Data Science Center", and the "Ministry of Science and Technology" for the grant support (MOST 110-2121-M-040-002)**.**

**BioVU**

This work is supported by National Library of Medicine R01-LM010685-11.

**Clinical Research Collaboration for Stroke in Korea (CRCS-K) and Korea Biobank Array (KBA) project**

This work was supported by funds by Research of Korea Centers for Disease Control and Prevention, Republic of Korea (2017ER620101#) and Korea Healthcare Technology R&D Project, Ministry of Health, Republic of Korea (HI10C2020), intramural grants from the National Institute of Health, Republic of Korea (2020-NI-026-02, 2022-NI-067-00). Genotype data were provided by the Collaborative Genome Program for Fostering New Post-Genome Industry (3000-3031b).

# 4. Other members of participating consortia

**Members of the China Kadoorie Biobank Collaborative Group**

**International Steering Committee:** Junshi Chen, Zhengming Chen (PI), Robert Clarke, Rory Collins, Yu Guo, Liming Li (PI), Chen Wang, Jun Lv, Richard Peto, Robin Walters.

**International Co-ordinating Centre, Oxford:** Daniel Avery, Derrick Bennett, Ruth Boxall, Ka Hung Chan, Yumei Chang, Yiping Chen, Zhengming Chen, Johnathan Clarke; Robert Clarke, Huaidong Du, Zammy Fairhurst-Hunter, Hannah Fry, Simon Gilbert, Alex Hacker, Mike Hill, Michael Holmes, Pek Kei Im, Andri Iona, Maria Kakkoura, Christiana Kartsonaki, Rene Kerosi, Kuang Lin, Mohsen Mazidi, Iona Millwood, Qunhua Nie, Alfred Pozarickij, Paul Ryder, Saredo Said, Sam Sansome, Dan Schmidt, Paul Sherliker, Rajani Sohoni, Becky Stevens, Iain Turnbull, Robin Walters, Lin Wang, Neil Wright, Ling Yang, Xiaoming Yang, Pang Yao.

**National Co-ordinating Centre, Beijing:** Yu Guo, Xiao Han, Can Hou, Chun Li, Chao Liu, Jun Lv, Pei Pei, Canqing Yu.

**Regional Co-ordinating Centres:**

**Gansu:** Gansu Provincial CDC – Caixia Dong, Pengfei Ge, Xiaolan Ren. Maiji CDC – Zhongxiao Li, Enke Mao, Tao Wang, Hui Zhang, Xi Zhang. **Haikou:** Hainan Provincial CDC – Jinyan Chen, Ximin Hu, Xiaohuan Wang. Meilan CDC – Zhendong Guo, Huimei Li, Yilei Li, Min Weng, Shukuan Wu. **Harbin:** Heilongjiang Provincial CDC – Shichun Yan, Mingyuan Zou, Xue Zhou. Nangang CDC – Ziyan Guo, Quan Kang, Yanjie Li, Bo Yu, Qinai Xu. **Henan:** Henan Provincial CDC – Liang Chang, Lei Fan, Shixian Feng, Ding Zhang, Gang Zhou. Huixian CDC – Yulian Gao, Tianyou He, Pan He, Chen Hu, Huarong Sun, Xukui Zhang. **Hunan:** Hunan Provincial CDC – Biyun Chen, Zhongxi Fu, Yuelong Huang, Huilin Liu, Qiaohua Xu, Li Yin. Liuyang CDC – Huajun Long, Xin Xu, Hao Zhang, Libo Zhang. **Liuzhou:** Guangxi Provincial CDC – Naying Chen, Duo Liu, Zhenzhu Tang. Liuzhou CDC – Ningyu Chen, Qilian Jiang, Jian Lan, Mingqiang Li, Yun Liu, Fanwen Meng, Jinhuai Meng, Rong Pan, Yulu Qin, Ping Wang, Sisi Wang, Liuping Wei, Liyuan Zhou. **Qingdao:** Qingdao CDC – Liang Cheng, Ranran Du, Ruqin Gao, Feifei Li, Shanpeng Li, Yongmei Liu, Feng Ning, Zengchang Pang, Xiaohui Sun, Xiaocao Tian, Shaojie Wang, Yaoming Zhai, Hua Zhang, Licang CDC – Wei Hou, Silu Lv, Junzheng Wang. **Sichuan:** Sichuan Provincial CDC – Xiaofang Chen, Xianping Wu, Ningmei Zhang, Weiwei Zhou. Pengzhou CDC – Xiaofang Chen, Jianguo Li, Jiaqiu Liu, Guojin Luo, Qiang Sun, Xunfu Zhong. **Suzhou:** Jiangsu Provincial CDC – Jian Su, Ran Tao, Ming Wu, Jie Yang, Jinyi Zhou, Yonglin Zhou. Suzhou CDC – Yihe Hu, Yujie Hua, Jianrong Jin Fang Liu, Jingchao Liu, Yan Lu, Liangcai Ma, Aiyu Tang, Jun Zhang. **Zhejiang**: Zhejiang Provincial CDC – Weiwei Gong, Ruying Hu, Hao Wang, Meng Wang, Min Yu. Tongxiang CDC – Lingli Chen, Qijun Gu, Dongxia Pan, Chunmei Wang, Kaixu Xie, Xiaoyi Zhang.

**VA Million Veteran Program**

**MVP Executive Committee**

- Co-Chair: J. Michael Gaziano, M.D., M.P.H.

VA Boston Healthcare System, 150 S. Huntington Avenue, Boston, MA 02130

- Co-Chair: Sumitra Muralidhar, Ph.D.

US Department of Veterans Affairs, 810 Vermont Avenue NW, Washington, DC 20420

- Rachel Ramoni, D.M.D., Sc.D., Chief VA Research and Development Officer

US Department of Veterans Affairs, 810 Vermont Avenue NW, Washington, DC 20420

- Jean Beckham, Ph.D.

Durham VA Medical Center, 508 Fulton Street, Durham, NC 27705

- Kyong-Mi Chang, M.D.

Philadelphia VA Medical Center, 3900 Woodland Avenue, Philadelphia, PA 19104

- Philip S. Tsao, Ph.D.

VA Palo Alto Health Care System, 3801 Miranda Avenue, Palo Alto, CA 94304

- James Breeling, M.D., Ex-Officio

US Department of Veterans Affairs, 810 Vermont Avenue NW, Washington, DC 20420

- Grant Huang, Ph.D., Ex-Officio

US Department of Veterans Affairs, 810 Vermont Avenue NW, Washington, DC 20420

- Juan P. Casas, M.D., Ph.D., Ex-Officio

VA Boston Healthcare System, 150 S. Huntington Avenue, Boston, MA 02130

**MVP Program Office**

- Sumitra Muralidhar, Ph.D.

US Department of Veterans Affairs, 810 Vermont Avenue NW, Washington, DC 20420

- Jennifer Moser, Ph.D.

US Department of Veterans Affairs, 810 Vermont Avenue NW, Washington, DC 20420

**MVP Recruitment/Enrollment**

- MVP Cohort Management Director/Recruitment/Enrollment Director, Boston – Stacey B. Whitbourne, Ph.D.; Jessica V. Brewer, M.P.H.

VA Boston Healthcare System, 150 S. Huntington Avenue, Boston, MA 02130

- VA Central Biorepository, Boston – Mary T. Brophy M.D., M.P.H.; Donald E. Humphries, Ph.D.; Luis E. Selva, Ph.D.

VA Boston Healthcare System, 150 S. Huntington Avenue, Boston, MA 02130

- MVP Informatics, Boston – Nhan Do, M.D.; Shahpoor (Alex) Shayan, M.S.

VA Boston Healthcare System, 150 S. Huntington Avenue, Boston, MA 02130

- MVP Data Operations/Analytics, Boston – Kelly Cho, M.P.H., Ph.D.

VA Boston Healthcare System, 150 S. Huntington Avenue, Boston, MA 02130

- Director of Regulatory Affairs – Lori Churby, B.S.

VA Palo Alto Health Care System, 3801 Miranda Avenue, Palo Alto, CA 94304

- MVP Coordinating Centers

- Cooperative Studies Program Clinical Research Pharmacy Coordinating Center, Albuquerque –Todd Connor, Pharm.D.; Dean P. Argyres, B.S., M.S. New Mexico VA Health Care System, 1501 San Pedro Drive SE, Albuquerque, NM 87108
- Genomics Coordinating Center, Palo Alto – Philip S. Tsao, Ph.D. VA Palo Alto Health Care System, 3801 Miranda Avenue, Palo Alto, CA 94304
- MVP Boston Coordinating Center, Boston - J. Michael Gaziano, M.D., M.P.H. VA Boston Healthcare System, 150 S. Huntington Avenue, Boston, MA 02130
- MVP Information Center, Canandaigua – Brady Stephens, M.S. Canandaigua VA Medical Center, 400 Fort Hill Avenue, Canandaigua, NY 14424

**MVP Science**

Saiju Pyarajan Ph.D.

VA Boston Healthcare System, 150 S. Huntington Avenue, Boston, MA 02130

Philip S. Tsao, Ph.D.

VA Palo Alto Health Care System, 3801 Miranda Avenue, Palo Alto, CA 94304

- Data Core - Kelly Cho, M.P.H, Ph.D.

VA Boston Healthcare System, 150 S. Huntington Avenue, Boston, MA 02130

- VA Informatics and Computing Infrastructure (VINCI) – Scott L. DuVall, Ph.D.

VA Salt Lake City Health Care System, 500 Foothill Drive, Salt Lake City, UT 84148

- Data and Computational Sciences – Saiju Pyarajan, Ph.D.

VA Boston Healthcare System, 150 S. Huntington Avenue, Boston, MA 02130

- Statistical Genetics – Elizabeth Hauser, Ph.D.

Durham VA Medical Center, 508 Fulton Street, Durham, NC 27705

Yan Sun, Ph.D.

Atlanta VA Medical Center, 1670 Clairmont Road, Decatur, GA 30033

Hongyu Zhao, Ph.D.

West Haven VA Medical Center, 950 Campbell Avenue, West Haven, CT 06516

**Current MVP Local Site Investigators**

- Atlanta VA Medical Center (Peter Wilson, M.D.)

1670 Clairmont Road, Decatur, GA 30033

- Bay Pines VA Healthcare System (Rachel McArdle, Ph.D.)

10,000 Bay Pines Blvd Bay Pines, FL 33744

- Birmingham VA Medical Center (Louis Dellitalia, M.D.)

700 S. 19th Street, Birmingham AL 35233

- Central Western Massachusetts Healthcare System (Kristin Mattocks, Ph.D., M.P.H.)

421 North Main Street, Leeds, MA 01053

- Cincinnati VA Medical Center (John Harley, M.D., Ph.D.)

3200 Vine Street, Cincinnati, OH 45220

- Clement J. Zablocki VA Medical Center (Jeffrey Whittle, M.D., M.P.H.)

5000 West National Avenue, Milwaukee, WI 53295

- VA Northeast Ohio Healthcare System (Frank Jacono, M.D.)

10701 East Boulevard, Cleveland, OH 44106

- Durham VA Medical Center (Jean Beckham, Ph.D.)

508 Fulton Street, Durham, NC 27705

- Edith Nourse Rogers Memorial Veterans Hospital (John Wells., Ph.D.)

200 Springs Road, Bedford, MA 01730

- Edward Hines, Jr. VA Medical Center (Salvador Gutierrez, M.D.)

5000 South 5th Avenue, Hines, IL 60141

- Veterans Health Care System of the Ozarks (Kathrina Alexander, M.D.)

1100 North College Avenue, Fayetteville, AR 72703

- Fargo VA Health Care System (Kimberly Hammer, Ph.D.)

2101 N. Elm, Fargo, ND 58102

- VA Health Care Upstate New York (James Norton, Ph.D.)

113 Holland Avenue, Albany, NY 12208

- New Mexico VA Health Care System (Gerardo Villareal, M.D.)

1501 San Pedro Drive, S.E. Albuquerque, NM 87108

- VA Boston Healthcare System (Scott Kinlay, M.B.B.S., Ph.D.)

150 S. Huntington Avenue, Boston, MA 02130

- VA Western New York Healthcare System (Junzhe Xu, M.D.)

3495 Bailey Avenue, Buffalo, NY 14215-1199

- Ralph H. Johnson VA Medical Center (Mark Hamner, M.D.)

109 Bee Street, Mental Health Research, Charleston, SC 29401

- Columbia VA Health Care System (Roy Mathew, M.D.)

6439 Garners Ferry Road, Columbia, SC 29209

- VA North Texas Health Care System (Sujata Bhushan, M.D.)

4500 S. Lancaster Road, Dallas, TX 75216

- Hampton VA Medical Center (Pran Iruvanti, D.O., Ph.D.)

100 Emancipation Drive, Hampton, VA 23667

- Richmond VA Medical Center (Michael Godschalk, M.D.)

1201 Broad Rock Blvd., Richmond, VA 23249

- Iowa City VA Health Care System (Zuhair Ballas, M.D.)

601 Highway 6 West, Iowa City, IA 52246-2208

- Eastern Oklahoma VA Health Care System (River Smith, Ph.D.)

1011 Honor Heights Drive, Muskogee, OK 74401

- James A. Haley Veterans’ Hospital (Stephen Mastorides, M.D.)

13000 Bruce B. Downs Blvd, Tampa, FL 33612

- James H. Quillen VA Medical Center (Jonathan Moorman, M.D., Ph.D.)

Corner of Lamont & Veterans Way, Mountain Home, TN 37684

- John D. Dingell VA Medical Center (Saib Gappy, M.D.)

4646 John R Street, Detroit, MI 48201

- Louisville VA Medical Center (Jon Klein, M.D., Ph.D.)

800 Zorn Avenue, Louisville, KY 40206

- Manchester VA Medical Center (Nora Ratcliffe, M.D.)

718 Smyth Road, Manchester, NH 03104

- Miami VA Health Care System (Ana Palacio, M.D., M.P.H.)

1201 NW 16th Street, 11 GRC, Miami FL 33125

- Michael E. DeBakey VA Medical Center (Olaoluwa Okusaga, M.D.)

2002 Holcombe Blvd, Houston, TX 77030

- Minneapolis VA Health Care System (Maureen Murdoch, M.D., M.P.H.)

One Veterans Drive, Minneapolis, MN 55417

- N. FL/S. GA Veterans Health System (Peruvemba Sriram, M.D.)

1601 SW Archer Road, Gainesville, FL 32608

- Northport VA Medical Center (Shing Shing Yeh, Ph.D., M.D.)

79 Middleville Road, Northport, NY 11768

- Overton Brooks VA Medical Center (Neeraj Tandon, M.D.)

510 East Stoner Ave, Shreveport, LA 71101

- Philadelphia VA Medical Center (Darshana Jhala, M.D.)

3900 Woodland Avenue, Philadelphia, PA 19104

- Phoenix VA Health Care System (Samuel Aguayo, M.D.)

650 E. Indian School Road, Phoenix, AZ 85012

- Portland VA Medical Center (David Cohen, M.D.)

3710 SW U.S. Veterans Hospital Road, Portland, OR 97239

- Providence VA Medical Center (Satish Sharma, M.D.)

830 Chalkstone Avenue, Providence, RI 02908

- Richard Roudebush VA Medical Center (Suthat Liangpunsakul, M.D., M.P.H.)

1481 West 10th Street, Indianapolis, IN 46202

- Salem VA Medical Center (Kris Ann Oursler, M.D.)

1970 Roanoke Blvd, Salem, VA 24153

- San Francisco VA Health Care System (Mary Whooley, M.D.)

4150 Clement Street, San Francisco, CA 94121

- South Texas Veterans Health Care System (Sunil Ahuja, M.D.)

7400 Merton Minter Boulevard, San Antonio, TX 78229

- Southeast Louisiana Veterans Health Care System (Joseph Constans, Ph.D.)

2400 Canal Street, New Orleans, LA 70119

- Southern Arizona VA Health Care System (Paul Meyer, M.D., Ph.D.)

3601 S 6th Avenue, Tucson, AZ 85723

- Sioux Falls VA Health Care System (Jennifer Greco, M.D.)

2501 W 22nd Street, Sioux Falls, SD 57105

- St. Louis VA Health Care System (Michael Rauchman, M.D.)

915 North Grand Blvd, St. Louis, MO 63106

- Syracuse VA Medical Center (Richard Servatius, Ph.D.)

800 Irving Avenue, Syracuse, NY 13210

- VA Eastern Kansas Health Care System (Melinda Gaddy, Ph.D.)

4101 S 4th Street Trafficway, Leavenworth, KS 66048

- VA Greater Los Angeles Health Care System (Agnes Wallbom, M.D., M.S.)

11301 Wilshire Blvd, Los Angeles, CA 90073

- VA Long Beach Healthcare System (Timothy Morgan, M.D.)

5901 East 7th Street Long Beach, CA 90822

- VA Maine Healthcare System (Todd Stapley, D.O.)

1 VA Center, Augusta, ME 04330

- VA New York Harbor Healthcare System (Peter Liang, M.D., M.P.H.)

423 East 23rd Street, New York, NY 10010

- VA Pacific Islands Health Care System (Daryl Fujii, Ph.D.)

459 Patterson Rd, Honolulu, HI 96819

- VA Palo Alto Health Care System (Philip Tsao, Ph.D.)

3801 Miranda Avenue, Palo Alto, CA 94304-1290

- VA Pittsburgh Health Care System (Patrick Strollo, Jr., M.D.)

University Drive, Pittsburgh, PA 15240

- VA Puget Sound Health Care System (Edward Boyko, M.D.)

1660 S. Columbian Way, Seattle, WA 98108-1597

- VA Salt Lake City Health Care System (Jessica Walsh, M.D.)

500 Foothill Drive, Salt Lake City, UT 84148

- VA San Diego Healthcare System (Samir Gupta, M.D., M.S.C.S.)

3350 La Jolla Village Drive, San Diego, CA 92161

- VA Sierra Nevada Health Care System (Mostaqul Huq, Pharm.D., Ph.D.)

975 Kirman Avenue, Reno, NV 89502

- VA Southern Nevada Healthcare System (Joseph Fayad, M.D.)

6900 North Pecos Road, North Las Vegas, NV 89086

- VA Tennessee Valley Healthcare System (Adriana Hung, M.D., M.P.H.)

1310 24th Avenue, South Nashville, TN 37212

- Washington DC VA Medical Center (Jack Lichy, M.D., Ph.D.)

50 Irving St, Washington, D. C. 20422

- W.G. (Bill) Hefner VA Medical Center (Robin Hurley, M.D.)

1601 Brenner Ave, Salisbury, NC 28144

- White River Junction VA Medical Center (Brooks Robey, M.D.)

163 Veterans Drive, White River Junction, VT 05009

- William S. Middleton Memorial Veterans Hospital (Prakash Balasubramanian, M.D.)

2500 Overlook Terrace, Madison, WI 53705

**Regeneron Genetics Center**

**RGC Management and Leadership Team**

Goncalo Abecasis, D.Phil. , Aris Baras, M.D. , Michael Cantor, M.D. , Giovanni Coppola, M.D. , Andrew Deubler , Aris Economides, Ph.D. , Katia Karalis, Ph.D. , Luca A. Lotta, M.D., Ph.D. , John D. Overton, Ph.D. , Jeffrey G. Reid, Ph.D. , Katherine Siminovitch, M.D. , Alan Shuldiner, M.D.

**Sequencing and Lab Operations**

Christina Beechert , Caitlin Forsythe, M.S. , Erin D. Fuller , Zhenhua Gu, M.S. , Michael Lattari , Alexander Lopez, M.S., John D. Overton, Ph.D. , Maria Sotiropoulos Padilla, M.S. , Manasi Pradhan, M.S. , Kia Manoochehri, B.S. , Thomas D. Schleicher, M.S. , Louis Widom , Sarah E. Wolf, M.S. , Ricardo H. Ulloa, B.S.

**Clinical Informatics**

Amelia Averitt, Ph.D. , Nilanjana Banerjee, Ph.D. , Michael Cantor, M.D. , Dadong Li, Ph.D. , Sameer Malhotra, M.D. , Deepika Sharma, MHI , Jeffrey Staples , Ph.D.

**Genome Informatics**

Xiaodong Bai, Ph.D. , Suganthi Balasubramanian, Ph.D. , Suying Bao, Ph.D. , Boris Boutkov, Ph.D. , Siying Chen, Ph.D. , Gisu Eom, B.S. , Lukas Habegger, Ph.D. , Alicia Hawes, B.S. , Shareef Khalid , Olga Krasheninina, M.S. , Rouel Lanche, B.S. , Adam J. Mansfield, B.A. , Evan K. Maxwell, Ph.D. , George Mitra, B.A. , Mona Nafde, M.S. , Sean O’Keeffe, Ph.D. , Max Orelus, B.B.A. , Razvan Panea, Ph.D. , Tommy Polanco, B.A. , Ayesha Rasool, M.S. , Jeffrey G. Reid, Ph.D. , William Salerno, Ph.D. , Jeffrey C. Staples, Ph.D. , Kathie Sun, Ph.D. , Jiwen Xin, Ph.D.

**Analytical Genomics and Data Science**

Goncalo Abecasis, D.Phil. , Joshua Backman, Ph.D. , Amy Damask, Ph.D. , Lee Dobbyn, Ph.D. , Manuel Allen Revez Ferreira, Ph.D. , Arkopravo Ghosh, M.S. , Christopher Gillies, Ph.D. , Lauren Gurski, B.S. , Eric Jorgenson, Ph.D. , Hyun Min Kang, Ph.D. , Michael Kessler, Ph.D. , Jack Kosmicki, Ph.D. , Alexander Li , Ph.D. , Nan Lin, Ph.D. , Daren Liu, M.S. , Adam Locke, Ph.D. , Jonathan Marchini, Ph.D. , Anthony Marcketta, M.S. , Joelle Mbatchou, Ph.D. , Arden Moscati, Ph.D. , Charles Paulding, Ph.D. , Carlo Sidore, Ph.D. , Eli Stahl, Ph.D. , Kyoko Watanabe, Ph.D. , Bin Ye, Ph.D. , Blair Zhang, Ph.D. , Andrey Ziyatdinov, Ph.D.

**Research Program Management & Strategic Initiatives**

Marcus B. Jones, Ph.D. , Jason Mighty, Ph.D. , Lyndon J. Mitnaul, Ph.D.

**List of Cardiovascular PIs for each EPIC Centre**:

Elisabete Weiderpass, IARC Director

Sherry Morris, EPIC Manager

**Denmark**

Aarhus: Kim Overvad, Christina Dahm

Copenhagen: Anne Tjønneland

**France**

Marie-Christine Boutron-Ruault

**Germany**

Heidelberg: Rudolf Kakks

Potsdam: Matthias Schulze

**Greece**

Antonia Trichopoulou

**IARC**

Pietro Ferrari

**Italy**

Florence: Giovanna Masala

Milan: Vittorio Krogh

Naples: Salvatore Panico

Ragusa: Rosario Tumino

Turin: Carlotta Sacerdote, Giuseppe Matullo

**Netherlands**

Bilthoven: Jolanda Boer

Utrecht: Yvonne van der Schouw

**Norway**

Guri Skeie

**Spain**

Asturias: J. Ramón Quirós

Barcelona: Granada Maria José Sánchez Pérez

Murcia: María Dolores Chirlaque

Navarra: Conchi Moreno

San Sebastian: Pilar Amiano, Liher Imaz

**Sweden**

Malmö: Olle Melander

Umeå: Patrik Wennberg

**United Kingdom**

EPIC-CVD: John Danesh

EPIC-InterAct: Nick Wareham

Imperial: Elio Riboli

Oxford: Tim Key

**FinnGen Contributors**

**Steering Committee**

Aarno Palotie Institute for Molecular Medicine Finland, HiLIFE, University of Helsinki, Finland

Mark Daly Institute for Molecular Medicine Finland, HiLIFE, University of Helsinki, Finland

**Pharmaceutical companies**

Bridget Riley-Gills Abbvie, Chicago, IL, United States

Howard Jacob Abbvie, Chicago, IL, United States

Dirk Paul Astra Zeneca, Cambridge, United Kingdom

Heiko Runz Biogen, Cambridge, MA, United States

Sally John Biogen, Cambridge, MA, United States

Robert Plenge Celgene, Summit, NJ, United States/Bristol Myers Squibb, New York, NY, United States

Mark McCarthy Genentech, San Francisco, CA, United States

Julie Hunkapiller Genentech, San Francisco, CA, United States Meg Ehm GlaxoSmithKline, Brentford, United Kingdom

Kirsi Auro GlaxoSmithKline, Brentford, United Kingdom Caroline Fox Merck, Kenilworth, NJ, United States

Anders Mälarstig Pfizer, New York, NY, United States Katherine Klinger Sanofi, Paris, France

Deepak Raipal Sanofi, Paris, France

Tim Behrens Maze Therapeutics, San Francisco, CA, United States Robert Yang Janssen Biotech, Beerse, Belgium

Richard Siegel Novartis, Basel, Switzerland University of Helsinki & Biobanks

Tomi Mäkelä HiLIFE, University of Helsinki, Finland, Finland

Jaakko Kaprio Institute for Molecular Medicine Finland, HiLIFE, Helsinki, Finland, Finland

Petri Virolainen Auria Biobank / University of Turku / Hospital District of Southwest Finland, Turku, Finland

Antti Hakanen Auria Biobank / University of Turku / Hospital District of Southwest Finland, Turku, Finland

Terhi Kilpi THL Biobank / The National Institute of Health and Welfare Helsinki, Finland Markus Perola THL Biobank / The National Institute of Health and Welfare Helsinki, Finland

Jukka Partanen Finnish Red Cross Blood Service / Finnish Hematology Registry and Clinical Biobank, Helsinki, Finland

Anne Pitkäranta Helsinki Biobank / Helsinki University and Hospital District of Helsinki and Uusimaa, Helsinki

Juhani Junttila Northern Finland Biobank Borealis / University of Oulu / Northern Ostrobothnia Hospital District, Oulu, Finland

Raisa Serpi Northern Finland Biobank Borealis / University of Oulu / Northern Ostrobothnia Hospital District, Oulu, Finland

Tarja Laitinen Finnish Clinical Biobank Tampere / University of Tampere / Pirkanmaa Hospital District, Tampere, Finland

Johanna Mäkelä Finnish Clinical Biobank Tampere / University of Tampere / Pirkanmaa Hospital District, Tampere, Finland

Veli-Matti Kosma Biobank of Eastern Finland / University of Eastern Finland / Northern Savo Hospital District, Kuopio, Finland

Urho Kujala Central Finland Biobank / University of Jyväskylä / Central Finland Health Care District, Jyväskylä, Finland

**Other Experts/ Non-Voting Members**

Outi Tuovila Business Finland, Helsinki, Finland

Raimo Pakkanen Business Finland, Helsinki, Finland

**Scientific Committee Pharmaceutical companies**

Jeffrey Waring Abbvie, Chicago, IL, United States

Ali Abbasi Abbvie, Chicago, IL, United States

Mengzhen Liu Abbvie, Chicago, IL, United States

Ioanna Tachmazidou Astra Zeneca, Cambridge, United Kingdom

Chia-Yen Chen Biogen, Cambridge, MA, United States Heiko Runz Biogen, Cambridge, MA, United States

Shameek Biswas Celgene, Summit, NJ, United States/Bristol Myers Squibb, New York, NY, United States Julie Hunkapiller Genentech, San Francisco, CA, United States

Meg Ehm GlaxoSmithKline, Brentford, United Kingdom

Neha Raghavan Merck, Kenilworth, NJ, United States

Adriana Huertas-Vazquez Merck, Kenilworth, NJ, United States Anders Mälarstig Pfizer, New York, NY, United States

Xinli Hu Pfizer, New York, NY, United States Katherine Klinger Sanofi, Paris, France

Matthias Gossel Sanofi, Paris, France

Robert Graham Maze Therapeutics, San Francisco, CA, United States

Tim Behrens Maze Therapeutics, San Francisco, CA, United States

Beryl Cummings Maze Therapeutics, San Francisco, CA, United States

Wilco Fleuren Janssen Biotech, Beerse, Belgium

Dawn Waterworth Janssen Biotech, Beerse, Belgium

Nicole Renaud Novartis, Basel, Switzerland

Aviv Madar Novartis, Basel, Switzerland

Maen Obeidat Novartis, Basel, Switzerland

**University of Helsinki & Biobanks**

Samuli Ripatti Institute for Molecular Medicine Finland, HiLIFE, Helsinki, Finland

Johanna Schleutker Auria Biobank / Univ. of Turku / Hospital District of Southwest Finland, Turku, Finland

Markus Perola THL Biobank / The National Institute of Health and Welfare Helsinki, Finland

Mikko Arvas Finnish Red Cross Blood Service / Finnish Hematology Registry and Clinical Biobank, Helsinki, Finland

Olli Carpén Helsinki Biobank / Helsinki University and Hospital District of Helsinki and Uusimaa, Helsinki

Reetta Hinttala Northern Finland Biobank Borealis / University of Oulu / Northern Ostrobothnia Hospital District, Oulu, Finland

Johannes Kettunen Northern Finland Biobank Borealis / University of Oulu / Northern Ostrobothnia Hospital District, Oulu, Finland

Johanna Mäkelä Finnish Clinical Biobank Tampere / University of Tampere / Pirkanmaa Hospital District, Tampere, Finland

Arto Mannermaa Biobank of Eastern Finland / University of Eastern Finland / Northern Savo Hospital District, Kuopio, Finland

Jari Laukkanen Central Finland Biobank / University of Jyväskylä / Central Finland Health Care District, Jyväskylä, Finland

Urho Kujala Central Finland Biobank / University of Jyväskylä / Central Finland Health Care District, Jyväskylä, Finland

**Clinical Groups**

***Neurology Group***

Reetta Kälviäinen Northern Savo Hospital District, Kuopio, Finland

Valtteri Julkunen Northern Savo Hospital District, Kuopio, Finland

Hilkka Soininen Northern Savo Hospital District, Kuopio, Finland

Anne Remes Northern Ostrobothnia Hospital District, Oulu, Finland

Mikko Hiltunen Northern Savo Hospital District, Kuopio, Finland

Jukka Peltola Pirkanmaa Hospital District, Tampere, Finland

Pentti Tienari Hospital District of Helsinki and Uusimaa, Helsinki, Finland

Juha Rinne Hospital District of Southwest Finland, Turku, Finland

Roosa Kallionpää Hospital District of Southwest Finland, Turku, Finland

Ali Abbasi Abbvie, Chicago, IL, United States

Adam Ziemann Abbvie, Chicago, IL, United States

Jeffrey Waring Abbvie, Chicago, IL, United States

Sahar Esmaeeli Abbvie, Chicago, IL, United States

Nizar Smaoui Abbvie, Chicago, IL, United States

Anne Lehtonen Abbvie, Chicago, IL, United States

Susan Eaton Biogen, Cambridge, MA, United States

Heiko Runz Biogen, Cambridge, MA, United States

Sanni Lahdenperä Biogen, Cambridge, MA, United States

Janet van Adelsberg Celgene, Summit, NJ, United States/ Bristol Myers Squibb, New York, NY, United States

Shameek Biswas Celgene, Summit, NJ, United States/ Bristol Myers Squibb, New York, NY, United States

Julie Hunkapiller Genentech, San Francisco, CA, United States

Natalie Bowers Genentech, San Francisco, CA, United States

Edmond Teng Genentech, San Francisco, CA, United States

Sarah Pendergrass Genentech, San Francisco, CA, United States

Onuralp Soylemez Merck, Kenilworth, NJ, United States

Kari Linden Pfizer, New York, NY, United States

Fanli Xu GlaxoSmithKline, Brentford, United Kingdom

David Pulford GlaxoSmithKline, Brentford, United Kingdom

Kirsi Auro GlaxoSmithKline, Brentford, United Kingdom

Laura Addis GlaxoSmithKline, Brentford, United Kingdom

John Eicher GlaxoSmithKline, Brentford, United Kingdom

Minna Raivio Hospital District of Helsinki and Uusimaa, Helsinki, Finland

Sarah Pendergrass Genentech, San Francisco, CA, United States

Beryl Cummings Maze Therapeutics, San Francisco, CA, United States

Juulia Partanen Institute for Molecular Medicine Finland, HiLIFE, University of Helsinki, Finland

***Gastroenterology Group***

Martti Färkkilä Hospital District of Helsinki and Uusimaa, Helsinki, Finland

Jukka Koskela Hospital District of Helsinki and Uusimaa, Helsinki, Finland

Sampsa Pikkarainen Hospital District of Helsinki and Uusimaa, Helsinki, Finland

Airi Jussila Pirkanmaa Hospital District, Tampere, Finland

Katri Kaukinen Pirkanmaa Hospital District, Tampere, Finland

Timo Blomster Northern Ostrobothnia Hospital District, Oulu, Finland

Mikko Kiviniemi Northern Savo Hospital District, Kuopio, Finland

Markku Voutilainen Hospital District of Southwest Finland, Turku, Finland

Ali Abbasi Abbvie, Chicago, IL, United States

Graham Heap Abbvie, Chicago, IL, United States

Jeffrey Waring Abbvie, Chicago, IL, United States

Nizar Smaoui Abbvie, Chicago, IL, United States

Fedik Rahimov Abbvie, Chicago, IL, United States

Anne Lehtonen Abbvie, Chicago, IL, United States

Keith Usiskin Celgene, Summit, NJ, United States/ Bristol Myers Squibb, New York, NY, United States

Tim Lu Genentech, San Francisco, CA, United States

Natalie Bowers Genentech, San Francisco, CA, United States

Danny Oh Genentech, San Francisco, CA, United States

Sarah Pendergrass Genentech, San Francisco, CA, United States

Kirsi Kalpala Pfizer, New York, NY, United States

Melissa Miller Pfizer, New York, NY, United States

Xinli Hu Pfizer, New York, NY, United States

Linda McCarthy GlaxoSmithKline, Brentford, United Kingdom

Onuralp Soylemez Merck, Kenilworth, NJ, United States

Mark Daly Institute for Molecular Medicine Finland, HiLIFE, University of Helsinki, Finland

**Rheumatology Group**

Kari Eklund Hospital District of Helsinki and Uusimaa, Helsinki, Finland

Antti Palomäki Hospital District of Southwest Finland, Turku, Finland

Pia Isomäki Pirkanmaa Hospital District, Tampere, Finland

Laura Pirilä Hospital District of Southwest Finland, Turku, Finland

Oili Kaipiainen-Seppänen Northern Savo Hospital District, Kuopio, Finland

Johanna Huhtakangas Northern Ostrobothnia Hospital District, Oulu, Finland

Ali Abbasi Abbvie, Chicago, IL, United States

Jeffrey Waring Abbvie, Chicago, IL, United States

Fedik Rahimov Abbvie, Chicago, IL, United States

Apinya Lertratanakul Abbvie, Chicago, IL, United States Nizar Smaoui Abbvie, Chicago, IL, United States

Anne Lehtonen Abbvie, Chicago, IL, United States

David Close Astra Zeneca, Cambridge, United Kingdom

Marla Hochfeld Celgene, Summit, NJ, United States/ Bristol Myers Squibb, New York, NY, United States

Natalie Bowers Genentech, San Francisco, CA, United States

Sarah Pendergrass Genentech, San Francisco, CA, United States

Onuralp Soylemez Merck, Kenilworth, NJ, United States

Kirsi Kalpala Pfizer, New York, NY, United States

Nan Bing Pfizer, New York, NY, United States

Xinli Hu Pfizer, New York, NY, United States

Jorge Esparza Gordillo GlaxoSmithKline, Brentford, United Kingdom

Kirsi Auro GlaxoSmithKline, Brentford, United Kingdom

Dawn Waterworth Janssen Biotech, Beerse, Belgium

Nina Mars Institute for Molecular Medicine Finland, HiLIFE, Helsinki, Finland

***Pulmonology Group***

Tarja Laitinen Pirkanmaa Hospital District, Tampere, Finland

Margit Pelkonen Northern Savo Hospital District, Kuopio, Finland

Paula Kauppi Hospital District of Helsinki and Uusimaa, Helsinki, Finland

Hannu Kankaanranta Pirkanmaa Hospital District, Tampere, Finland

Terttu Harju Northern Ostrobothnia Hospital District, Oulu, Finland

Riitta Lahesmaa Hospital District of Southwest Finland, Turku, Finland

Nizar Smaoui Abbvie, Chicago, IL, United States

Alex Mackay Astra Zeneca, Cambridge, United Kingdom

Glenda Lassi Astra Zeneca, Cambridge, United Kingdom

Susan Eaton Biogen, Cambridge, MA, United States

Steven Greenberg Celgene, Summit, NJ, United States/ Bristol Myers Squibb, New York, NY, United States

Hubert Chen Genentech, San Francisco, CA, United States

Sarah Pendergrass Genentech, San Francisco, CA, United States

Natalie Bowers Genentech, San Francisco, CA, United States

Joanna Betts GlaxoSmithKline, Brentford, United Kingdom

Soumitra Ghosh GlaxoSmithKline, Brentford, United Kingdom

Kirsi Auro GlaxoSmithKline, Brentford, United Kingdom

Rajashree Mishra GlaxoSmithKline, Brentford, United Kingdom

Sina Rüeger Institute for Molecular Medicine Finland, HiLIFE, University of Helsinki, Finland

***Cardiometabolic Diseases Group***

Teemu Niiranen The National Institute of Health and Welfare Helsinki, Finland

Felix Vaura The National Institute of Health and Welfare Helsinki, Finland

Veikko Salomaa The National Institute of Health and Welfare Helsinki, Finland

Markus Juonala Hospital District of Southwest Finland, Turku, Finland

Kaj Metsärinne Hospital District of Southwest Finland, Turku, Finland

Mika Kähönen Pirkanmaa Hospital District, Tampere, Finland

Juhani Junttila Northern Ostrobothnia Hospital District, Oulu, Finland

Markku Laakso Northern Savo Hospital District, Kuopio, Finland

Jussi Pihlajamäki Northern Savo Hospital District, Kuopio, Finland

Daniel Gordin Hospital District of Helsinki and Uusimaa, Helsinki, Finland

Juha Sinisalo Hospital District of Helsinki and Uusimaa, Helsinki, Finland

Marja-Riitta Taskinen Hospital District of Helsinki and Uusimaa, Helsinki, Finland

Tiinamaija Tuomi Hospital District of Helsinki and Uusimaa, Helsinki, Finland

Jari Laukkanen Central Finland Health Care District, Jyväskylä, Finland

Benjamin Challis Astra Zeneca, Cambridge, United Kingdom

Dirk Paul Astra Zeneca, Cambridge, United Kingdom

Julie Hunkapiller Genentech, San Francisco, CA, United States

Natalie Bowers Genentech, San Francisco, CA, United States

Sarah Pendergrass Genentech, San Francisco, CA, United States

Onuralp Soylemez Merck, Kenilworth, NJ, United States

Jaakko Parkkinen Pfizer, New York, NY, United States

Melissa Miller Pfizer, New York, NY, United States

Russell Miller Pfizer, New York, NY, United States

Audrey Chu GlaxoSmithKline, Brentford, United Kingdom

Kirsi Auro GlaxoSmithKline, Brentford, United Kingdom

Keith Usiskin Celgene, Summit, NJ, United States/ Bristol Myers Squibb, New York, NY, United States

Amanda Elliott Institute for Molecular Medicine Finland, HiLIFE, University of Helsinki, Finland / Broad Institute, Cambridge, MA, United States

Joel Rämö Institute for Molecular Medicine Finland, HiLIFE, University of Helsinki, Finland

Samuli Ripatti Institute for Molecular Medicine Finland, HiLIFE, University of Helsinki, Finland

Mary Pat Reeve Institute for Molecular Medicine Finland, HiLIFE, University of Helsinki, Finland

Sanni Ruotsalainen Institute for Molecular Medicine Finland, HiLIFE, University of Helsinki, Finland

***Oncology Group***

Tuomo Meretoja Hospital District of Helsinki and Uusimaa, Helsinki, Finland

Heikki Joensuu Hospital District of Helsinki and Uusimaa, Helsinki, Finland

Olli Carpén Hospital District of Helsinki and Uusimaa, Helsinki, Finland

Lauri Aaltonen Hospital District of Helsinki and Uusimaa, Helsinki, Finland

Johanna Mattson Hospital District of Helsinki and Uusimaa, Helsinki, Finland

Annika Auranen Pirkanmaa Hospital District , Tampere, Finland

Peeter Karihtala Northern Ostrobothnia Hospital District, Oulu, Finland

Saila Kauppila Northern Ostrobothnia Hospital District, Oulu, Finland

Päivi Auvinen Northern Savo Hospital District, Kuopio, Finland

Klaus Elenius Hospital District of Southwest Finland, Turku, Finland

Johanna Schleutker Hospital District of Southwest Finland, Turku, Finland

Relja Popovic Abbvie, Chicago, IL, United States

Jeffrey Waring Abbvie, Chicago, IL, United States Bridget Riley-Gillis Abbvie, Chicago, IL, United States

Anne Lehtonen Abbvie, Chicago, IL, United States

Jennifer Schutzman Genentech, San Francisco, CA, United States

Julie Hunkapiller Genentech, San Francisco, CA, United States

Natalie Bowers Genentech, San Francisco, CA, United States

Sarah Pendergrass Genentech, San Francisco, CA, United States

Andrey Loboda Merck, Kenilworth, NJ, United States

Aparna Chhibber Merck, Kenilworth, NJ, United States

Heli Lehtonen Pfizer, New York, NY, United States

Stefan McDonough Pfizer, New York, NY, United States

Marika Crohns Sanofi, Paris, France

Sauli Vuoti Sanofi, Paris, France

Diptee Kulkarni GlaxoSmithKline, Brentford, United Kingdom

Kirsi Auro GlaxoSmithKline, Brentford, United Kingdom

Esa Pitkänen Institute for Molecular Medicine Finland, HiLIFE, University of Helsinki, Finland

Nina Mars Institute for Molecular Medicine Finland, HiLIFE, University of Helsinki, Finland

Mark Daly Institute for Molecular Medicine Finland, HiLIFE, University of Helsinki, Finland

***Opthalmology Group***

Kai Kaarniranta Northern Savo Hospital District, Kuopio, Finland

Joni A Turunen Hospital District of Helsinki and Uusimaa, Helsinki, Finland

Terhi Ollila Hospital District of Helsinki and Uusimaa, Helsinki, Finland

Sanna Seitsonen Hospital District of Helsinki and Uusimaa, Helsinki, Finland

Hannu Uusitalo Pirkanmaa Hospital District, Tampere, Finland

Vesa Aaltonen Hospital District of Southwest Finland, Turku, Finland

Hannele Uusitalo-Järvinen Pirkanmaa Hospital District, Tampere, Finland

Marja Luodonpää Northern Ostrobothnia Hospital District, Oulu, Finland

Nina Hautala Northern Ostrobothnia Hospital District, Oulu, Finland

Mengzhen Liu Abbvie, Chicago, IL, United States

Heiko Runz Biogen, Cambridge, MA, United States

Stephanie Loomis Biogen, Cambridge, MA, United States

Erich Strauss Genentech, San Francisco, CA, United States

Natalie Bowers Genentech, San Francisco, CA, United States

Hao Chen Genentech, San Francisco, CA, United States

Sarah Pendergrass Genentech, San Francisco, CA, United States

Anna Podgornaia Merck, Kenilworth, NJ, United States

Juha Karjalainen Institute for Molecular Medicine Finland, HiLIFE, University of Helsinki, Finland / Broad Institute, Cambridge, MA, United States

Esa Pitkänen Institute for Molecular Medicine Finland, HiLIFE, University of Helsinki, Finland

***Dermatology Group***

Kaisa Tasanen Northern Ostrobothnia Hospital District, Oulu, Finland

Laura Huilaja Northern Ostrobothnia Hospital District, Oulu, Finland

Katariina Hannula-Jouppi Hospital District of Helsinki and Uusimaa, Helsinki, Finland

Teea Salmi Pirkanmaa Hospital District, Tampere, Finland

Sirkku Peltonen Hospital District of Southwest Finland, Turku, Finland

Leena Koulu Hospital District of Southwest Finland, Turku, Finland

Kirsi Kalpala Pfizer, New York, NY, United States

Ying Wu Pfizer, New York, NY, United States

David Choy Genentech, San Francisco, CA, United States

Sarah Pendergrass Genentech, San Francisco, CA, United States

Nizar Smaoui Abbvie, Chicago, IL, United States

Fedik Rahimov Abbvie, Chicago, IL, United States

Anne Lehtonen Abbvie, Chicago, IL, United States

Dawn Waterworth Janssen Biotech, Beerse, Belgium

***Odontology Group***

Pirkko Pussinen Hospital District of Helsinki and Uusimaa, Helsinki, Finland

Aino Salminen Hospital District of Helsinki and Uusimaa, Helsinki, Finland

Tuula Salo Hospital District of Helsinki and Uusimaa, Helsinki, Finland

David Rice Hospital District of Helsinki and Uusimaa, Helsinki, Finland

Pekka Nieminen Hospital District of Helsinki and Uusimaa, Helsinki, Finland

Ulla Palotie Hospital District of Helsinki and Uusimaa, Helsinki, Finland

Juha Sinisalo Hospital District of Helsinki and Uusimaa, Helsinki, Finland

Maria Siponen Northern Savo Hospital District, Kuopio, Finland

Liisa Suominen Northern Savo Hospital District, Kuopio, Finland

Päivi Mäntylä Northern Savo Hospital District, Kuopio, Finland

Ulvi Gursoy Hospital District of Southwest Finland, Turku, Finland

Vuokko Anttonen Northern Ostrobothnia Hospital District, Oulu, Finland

Kirsi Sipilä Northern Ostrobothnia Hospital District, Oulu, Finland

Sarah Pendergrass Genentech, San Francisco, CA, United States

***Women’s Health and Reproduction Group***

Hannele Laivuori Institute for Molecular Medicine Finland, HiLIFE, University of Helsinki, Finland Venla Kurra Pirkanmaa Hospital District, Tampere, Finland

Oskari Heikinheimo Hospital District of Helsinki and Uusimaa, Helsinki, Finland

Ilkka Kalliala Hospital District of Helsinki and Uusimaa, Helsinki, Finland

Laura Kotaniemi-Talonen Pirkanmaa Hospital District, Tampere, Finland

Kari Nieminen Pirkanmaa Hospital District, Tampere, Finland

Päivi Polo Hospital District of Southwest Finland, Turku, Finland

Kaarin Mäkikallio Hospital District of Southwest Finland, Turku, Finland

Eeva Ekholm Hospital District of Southwest Finland, Turku, Finland

Marja Vääräsmäki Northern Ostrobothnia Hospital District, Oulu, Finland

Outi Uimari Northern Ostrobothnia Hospital District, Oulu, Finland

Laure Morin-Papunen Northern Ostrobothnia Hospital District, Oulu, Finland

Marjo Tuppurainen Northern Savo Hospital District, Kuopio, Finland

Katja Kivinen Institute for Molecular Medicine Finland, HiLIFE, University of Helsinki, Finland

Elisabeth Widen Institute for Molecular Medicine Finland, HiLIFE, University of Helsinki, Finland

Taru Tukiainen Institute for Molecular Medicine Finland, HiLIFE, University of Helsinki, Finland

Mary Pat Reeve Institute for Molecular Medicine Finland, HiLIFE, University of Helsinki, Finland

Mark Daly Institute for Molecular Medicine Finland, HiLIFE, University of Helsinki, Finland

Liu Aoxing Institute for Molecular Medicine Finland, HiLIFE, University of Helsinki, Finland

Eija Laakkonen University of Jyväskylä, Jyväskylä, Finland

Niko Välimäki University of Helsinki, Helsinki, Finland

Lauri Aaltonen Hospital District of Helsinki and Uusimaa, Helsinki, Finland

Johannes Kettunen Northern Ostrobothnia Hospital District, Oulu, Finland

Mikko Arvas Finnish Red Cross Blood Service, Helsinki, Finland

Jeffrey Waring Abbvie, Chicago, IL, United States

Bridget Riley-Gillis Abbvie, Chicago, IL, United States

Mengzhen Liu Abbvie, Chicago, IL, United States

Janet Kumar GlaxoSmithKline, Brentford, United Kingdom

Kirsi Auro GlaxoSmithKline, Brentford, United Kingdom

Andrea Ganna Institute for Molecular Medicine Finland, HiLIFE, University of Helsinki, Finland

Sarah Pendergrass Genentech, San Francisco, CA, United States

**FinnGen Analysis working group**

Justin Wade Davis Abbvie, Chicago, IL, United States

Bridget Riley-Gillis Abbvie, Chicago, IL, United States

Danjuma Quarless Abbvie, Chicago, IL, United States

Fedik Rahimov Abbvie, Chicago, IL, United States

Sahar Esmaeeli Abbvie, Chicago, IL, United States

Slavé Petrovski Astra Zeneca, Cambridge, United Kingdom

Eleonor Wigmore Astra Zeneca, Cambridge, United Kingdom

Adele Mitchell Biogen, Cambridge, MA, United States

Benjamin Sun Biogen, Cambridge, MA, United States

Ellen Tsai Biogen, Cambridge, MA, United States

Denis Baird Biogen, Cambridge, MA, United States

Paola Bronson Biogen, Cambridge, MA, United States

Ruoyu Tian Biogen, Cambridge, MA, United States

Stephanie Loomis Biogen, Cambridge, MA, United States

Yunfeng Huang Biogen, Cambridge, MA, United States

Joseph Maranville Celgene, Summit, NJ, United States/ Bristol Myers Squibb, New York, NY, United States

Shameek Biswas Celgene, Summit, NJ, United States/ Bristol Myers Squibb, New York, NY, United States

Elmutaz Mohammed Celgene, Summit, NJ, United States/ Bristol Myers Squibb, New York, NY, United States

Samir Wadhawan Celgene, Summit, NJ, United States/ Bristol Myers Squibb, New York, NY, United States

Erika Kvikstad Celgene, Summit, NJ, United States/ Bristol Myers Squibb, New York, NY, United States

Minal Caliskan Celgene, Summit, NJ, United States/ Bristol Myers Squibb, New York, NY, United States

Diana Chang Genentech, San Francisco, CA, United States

Julie Hunkapiller Genentech, San Francisco, CA, United States

Tushar Bhangale Genentech, San Francisco, CA, United States

Natalie Bowers Genentech, San Francisco, CA, United States

Sarah Pendergrass Genentech, San Francisco, CA, United States

Kirill Shkura Merck, Kenilworth, NJ, United States

Victor Neduva Merck, Kenilworth, NJ, United States

Xing Chen Pfizer, New York, NY, United States

Åsa Hedman Pfizer, New York, NY, United States

Karen S King GlaxoSmithKline, Brentford, United Kingdom

Padhraig Gormley GlaxoSmithKline, Brentford, United Kingdom

Jimmy Liu GlaxoSmithKline, Brentford, United Kingdom

Clarence Wang Sanofi, Paris, France

Ethan Xu Sanofi, Paris, France

Franck Auge Sanofi, Paris, France Clement Chatelain Sanofi, Paris, France

Deepak Rajpal Sanofi, Paris, France

Dongyu Liu Sanofi, Paris, France

Katherine Call Sanofi, Paris, France

Tai-He Xia Sanofi, Paris, France

Beryl Cummings Maze Therapeutics, San Francisco, CA, United States

Matt Brauer Maze Therapeutics, San Francisco, CA, United States

Huilei Xu Novartis, Basel, Switzerland

Amy Cole Novartis, Basel, Switzerland

Jonathan Chung Novartis, Basel, Switzerland

Jaison Jacob Novartis, Basel, Switzerland

Katrina de Lange Novartis, Basel, Switzerland

Jonas Zierer Novartis, Basel, Switzerland

Mitja Kurki Institute for Molecular Medicine Finland, HiLIFE, University of Helsinki, Finland / Broad Institute, Cambridge, MA, United States

Samuli Ripatti Institute for Molecular Medicine Finland, HiLIFE, University of Helsinki, Finland

Mark Daly Institute for Molecular Medicine Finland, HiLIFE, University of Helsinki, Finland

Juha Karjalainen Institute for Molecular Medicine Finland, HiLIFE, University of Helsinki, Finland/ Broad Institute, Cambridge, MA, United States

Aki Havulinna Institute for Molecular Medicine Finland, HiLIFE, University of Helsinki, Finland

Juha Mehtonen Institute for Molecular Medicine Finland, HiLIFE, University of Helsinki, Finland

Priit Palta Institute for Molecular Medicine Finland, HiLIFE, University of Helsinki, Finland

Shabbeer Hassan Institute for Molecular Medicine Finland, HiLIFE, University of Helsinki, Finland

Pietro Della Briotta Parolo Institute for Molecular Medicine Finland, HiLIFE, University of Helsinki, Finland

Wei Zhou Broad Institute, Cambridge, MA, United States

Mutaamba Maasha Broad Institute, Cambridge, MA, United States

Shabbeer Hassan Institute for Molecular Medicine Finland, HiLIFE, University of Helsinki, Finland

Susanna Lemmelä Institute for Molecular Medicine Finland, HiLIFE, University of Helsinki, Finland

Manuel Rivas University of Stanford, Stanford, CA, United States

Aarno Palotie Institute for Molecular Medicine Finland, HiLIFE, University of Helsinki, Finland

Arto Lehisto Institute for Molecular Medicine Finland, HiLIFE, University of Helsinki, Finland

Andrea Ganna Institute for Molecular Medicine Finland, HiLIFE, University of Helsinki, Finland

Vincent Llorens Institute for Molecular Medicine Finland, HiLIFE, University of Helsinki, Finland

Hannele Laivuori Institute for Molecular Medicine Finland, HiLIFE, University of Helsinki, Finland

Mari E Niemi Institute for Molecular Medicine Finland, HiLIFE, University of Helsinki, Finland

Taru Tukiainen Institute for Molecular Medicine Finland, HiLIFE, University of Helsinki, Finland

Mary Pat Reeve Institute for Molecular Medicine Finland, HiLIFE, University of Helsinki, Finland

Henrike Heyne Institute for Molecular Medicine Finland, HiLIFE, University of Helsinki, Finland

Nina Mars Institute for Molecular Medicine Finland, HiLIFE, University of Helsinki, Finland

Kimmo Palin University of Helsinki, Helsinki, Finland

Javier Garcia-Tabuenca University of Tampere, Tampere, Finland

Harri Siirtola University of Tampere, Tampere, Finland

Tuomo Kiiskinen Institute for Molecular Medicine Finland, HiLIFE, University of Helsinki, Finland

Jiwoo Lee Institute for Molecular Medicine Finland, HiLIFE, University of Helsinki, Finland / Broad Institute, Cambridge, MA, United States

Kristin Tsuo Institute for Molecular Medicine Finland, HiLIFE, University of Helsinki, Finland / Broad Institute, Cambridge, MA, United States

Amanda Elliott Institute for Molecular Medicine Finland, HiLIFE, University of Helsinki, Finland / Broad Institute, Cambridge, MA, United States

Kati Kristiansson THL Biobank / The National Institute of Health and Welfare Helsinki, Finland

Mikko Arvas Finnish Red Cross Blood Service / Finnish Hematology Registry and Clinical Biobank, Helsinki, Finland

Kati Hyvärinen Finnish Red Cross Blood Service, Helsinki, Finland

Jarmo Ritari Finnish Red Cross Blood Service, Helsinki, Finland

Miika Koskinen Helsinki Biobank / Helsinki University and Hospital District of Helsinki and Uusimaa, Helsinki

Olli Carpén Helsinki Biobank / Helsinki University and Hospital District of Helsinki and Uusimaa, Helsinki

Johannes Kettunen Northern Finland Biobank Borealis / University of Oulu / Northern Ostrobothnia Hospital District, Oulu, Finland

Katri Pylkäs University of Oulu, Oulu, Finland

Marita Kalaoja University of Oulu, Oulu, Finland

Minna Karjalainen University of Oulu, Oulu, Finland

Tuomo Mantere Northern Finland Biobank Borealis / University of Oulu / Northern Ostrobothnia Hospital District, Oulu, Finland

Eeva Kangasniemi Finnish Clinical Biobank Tampere / University of Tampere / Pirkanmaa Hospital District, Tampere, Finland

Sami Heikkinen University of Eastern Finland, Kuopio, Finland

Arto Mannermaa Biobank of Eastern Finland / University of Eastern Finland / Northern Savo Hospital District, Kuopio, Finland

Eija Laakkonen University of Jyväskylä, Jyväskylä, Finland

Samuel Heron University of Turku, Turku, Finland

Dhanaprakash Jambulingam University of Turku, Turku, Finland

Venkat Subramaniam Rathinakannan University of Turku, Turku, Finland

Nina Pitkänen Auria Biobank / University of Turku / Hospital District of Southwest Finland, Turku, Finland

**Biobank directors**

Lila Kallio Auria Biobank / University of Turku / Hospital District of Southwest Finland, Turku, Finland

Sirpa Soini THL Biobank / The National Institute of Health and Welfare Helsinki, Finland

Jukka Partanen Finnish Red Cross Blood Service / Finnish Hematology Registry and Clinical Biobank, Helsinki, Finland

Eero Punkka Helsinki Biobank / Helsinki University and Hospital District of Helsinki and Uusimaa, Helsinki

Raisa Serpi Northern Finland Biobank Borealis / University of Oulu / Northern Ostrobothnia Hospital District, Oulu, Finland

Johanna Mäkelä Finnish Clinical Biobank Tampere / University of Tampere / Pirkanmaa Hospital District, Tampere, Finland

Veli-Matti Kosma Biobank of Eastern Finland / University of Eastern Finland / Northern Savo Hospital District, Kuopio, Finland

Teijo Kuopio Central Finland Biobank / University of Jyväskylä / Central Finland Health Care District, Jyväskylä, Finland

**FinnGen Teams Administration**

Anu Jalanko Institute for Molecular Medicine Finland, HiLIFE, University of Helsinki, Finland Huei-Yi Shen Institute for Molecular Medicine Finland, HiLIFE, University of Helsinki, Finland Risto Kajanne Institute for Molecular Medicine Finland, HiLIFE, University of Helsinki, Finland Mervi Aavikko Institute for Molecular Medicine Finland, HiLIFE, University of Helsinki, Finland

**Analysis**

Mitja Kurki Institute for Molecular Medicine Finland, HiLIFE, University of Helsinki, Finland / Broad Institute, Cambridge, MA, United States

Juha Karjalainen Institute for Molecular Medicine Finland, HiLIFE, University of Helsinki, Finland / Broad Institute, Cambridge, MA, United States

Pietro Della Briotta Parolo Institute for Molecular Medicine Finland, HiLIFE, University of Helsinki, Finland

Arto Lehisto Institute for Molecular Medicine Finland, HiLIFE, University of Helsinki, Finland

Juha Mehtonen Institute for Molecular Medicine Finland, HiLIFE, University of Helsinki, Finland

Wei Zhou Broad Institute, Cambridge, MA, United States

Masahiro Kanai Broad Institute, Cambridge, MA, United States

Mutaamba Maasha Broad Institute, Cambridge, MA, United States

**Clinical Endpoint Development**

Hannele Laivuori Institute for Molecular Medicine Finland, HiLIFE, University of Helsinki, Finland

Aki Havulinna Institute for Molecular Medicine Finland, HiLIFE, University of Helsinki, Finland

Susanna Lemmelä Institute for Molecular Medicine Finland, HiLIFE, University of Helsinki, Finland

Tuomo Kiiskinen Institute for Molecular Medicine Finland, HiLIFE, University of Helsinki, Finland

L. Elisa Lahtela Institute for Molecular Medicine Finland, HiLIFE, University of Helsinki, Finland

Matti Peura Institute for Molecular Medicine Finland, HiLIFE, University of Helsinki, Finland

**Communication**

Mari Kaunisto Institute for Molecular Medicine Finland, HiLIFE, University of Helsinki, Finland

**Data Management and IT Infrastructure**

Elina Kilpeläinen Institute for Molecular Medicine Finland, HiLIFE, University of Helsinki, Finland

Timo P. Sipilä Institute for Molecular Medicine Finland, HiLIFE, University of Helsinki, Finland

Georg Brein Institute for Molecular Medicine Finland, HiLIFE, University of Helsinki, Finland

Oluwaseun A. Dada Institute for Molecular Medicine Finland, HiLIFE, University of Helsinki, Finland

Awaisa Ghazal Institute for Molecular Medicine Finland, HiLIFE, University of Helsinki, Finland

Anastasia Shcherban Institute for Molecular Medicine Finland, HiLIFE, University of Helsinki, Finland

**Genotyping**

Kati Donner Institute for Molecular Medicine Finland, HiLIFE, University of Helsinki, Finland Timo P. Sipilä Institute for Molecular Medicine Finland, HiLIFE, University of Helsinki, Finland

**Sample Collection Coordination**

Anu Loukola Helsinki Biobank / Helsinki University and Hospital District of Helsinki and Uusimaa, Helsinki

**Sample Logistics**

Päivi Laiho THL Biobank / The National Institute of Health and Welfare Helsinki, Finland

Tuuli Sistonen THL Biobank / The National Institute of Health and Welfare Helsinki, Finland

Essi Kaiharju THL Biobank / The National Institute of Health and Welfare Helsinki, Finland Markku Laukkanen THL Biobank / The National Institute of Health and Welfare Helsinki, Finland Elina Järvensivu THL Biobank / The National Institute of Health and Welfare Helsinki, Finland Sini Lähteenmäki THL Biobank / The National Institute of Health and Welfare Helsinki, Finland Lotta Männikkö THL Biobank / The National Institute of Health and Welfare Helsinki, Finland Regis Wong THL Biobank / The National Institute of Health and Welfare Helsinki, Finland

**Registry Data Operations**

Hannele Mattsson THL Biobank / The National Institute of Health and Welfare Helsinki, Finland

Kati Kristiansson THL Biobank / The National Institute of Health and Welfare Helsinki, Finland

Susanna Lemmelä Institute for Molecular Medicine Finland, HiLIFE, University of Helsinki, Finland

Sami Koskelainen THL Biobank / The National Institute of Health and Welfare Helsinki, Finland

Tero Hiekkalinna THL Biobank / The National Institute of Health and Welfare Helsinki, Finland

Teemu Paajanen THL Biobank / The National Institute of Health and Welfare Helsinki, Finland

**Sequencing Informatics**

Priit Palta Institute for Molecular Medicine Finland, HiLIFE, University of Helsinki, Finland

Kalle Pärn Institute for Molecular Medicine Finland, HiLIFE, University of Helsinki, Finland

Shuang Luo Institute for Molecular Medicine Finland, HiLIFE, University of Helsinki, Finland

Vishal Sinha Institute for Molecular Medicine Finland, HiLIFE, University of Helsinki, Finland

**Trajectory Team**

Tarja Laitinen Pirkanmaa Hospital District, Tampere, Finland

Harri Siirtola University of Tampere, Tampere, Finland

Javier Gracia-Tabuenca University of Tampere, Tampere, Finland

Mika Helminen University of Tampere, Tampere, Finland

Tiina Luukkaala University of Tampere, Tampere, Finland

Iida Vähätalo University of Tampere, Tampere, Finland

**Data protection officer**

Tero Jyrhämä Institute for Molecular Medicine Finland, HiLIFE, University of Helsinki, Finland

**FinBB - Finnish biobank cooperative**

Marco Hautalahti

Laura Mustaniemi

Mirkka Koivusalo

Sarah Smith

Tom Southerington

**Stroke Investigative Research and Educational Network (SIREN) Contributors**

Hemant K. Tiwari^1^, Rufus Akinyemi^2,3^, Vinodh Srinivasasainagendra^1^, Onoja Akpa^4,5^, Fred S. Sarfo^6^, Albert Akpalu^7^, Reginald Obiako^8^, Kolawole Wahab^9^, Godwin Osaigbovo^10^, Lukman Owolabi^11^, Morenikeji Komolafe^12^, Carolyn Jenkins^13^, Oyedunni Arulogun^14^, Godwin Ogbole^15^, Abiodun M. Adeoye^16^, Joshua Akinyemi^17^, Atinuke Agunloye^16^, Adekunle G. Fakunle^16^, Ezinne Uvere^16^, Abimbola Olalere^16^, Olayinka J. Adebajo^16^, Donna Arnett^18^, Bruce Ovbiagele^19^, Mayowa O. Owolabi^2,16^

1 School of Public Health, University of Alabama at Birmingham, USA

2 Center for Genomic and Precision Medicine, College of Medicine, University of Ibadan, Nigeria

3 Neuroscience and Ageing Research Unit Institute for Advanced Medical Research and Training, College of Medicine, University of Ibadan, Nigeria

4 Department of Epidemiology and Medical Statistics, University of Ibadan

5 Institute of Cardiovascular Diseases, University of Ibadan, Nigeria

6 Department of Medicine, Kwame Nkrumah University of Science and Technology, Ghana.

7 Department of Medicine, University of Ghana Medical School, Accra, Ghana

8 Department of Medicine, Ahmadu Bello University, Zaria, Nigeria

9 Department of Medicine, University of Ilorin Teaching Hospital, Ilorin, Nigeria

10 Jos University Teaching Hospital Jos, Nigeria

11 Department of Medicine, Aminu Kano Teaching Hospital, Kano, Nigeria

12 Department of Medicine, Obafemi Awolowo University Teaching Hospital, Ile-Ife, Nigeria

13 Medical University of South Carolina, South Carolina, USA

14 Department of Health Promotion and Education, University of Ibadan, Nigeria

15 Department of Radiology, College of Medicine, University of Ibadan, Nigeria

16 Department of Medicine, University of Ibadan, Nigeria

17 MRC Epidemiology Unit, University of Cambridge School of Clinical Medicine, Institute of Metabolic Science, Cambridge, UK.

18 Provost, University of South Carolina, USA

19 Weill Institute for Neurosciences, University of California San-Francisco, USA

# 5. References

1 Kawai, Y. *et al.* Japonica array: improved genotype imputation by designing a population-specific SNP array with 1070 Japanese individuals. *J Hum Genet* **60**, 581-587, doi:10.1038/jhg.2015.68 (2015).

2 Chang, C. C. *et al.* Second-generation PLINK: rising to the challenge of larger and richer datasets. *Gigascience* **4**, 7, doi:10.1186/s13742-015-0047-8 (2015).

3 Loh, P. R., Palamara, P. F. & Price, A. L. Fast and accurate long-range phasing in a UK Biobank cohort. *Nat Genet* **48**, 811-816, doi:10.1038/ng.3571 (2016).

4 Das, S. *et al.* Next-generation genotype imputation service and methods. *Nat Genet* **48**, 1284-1287, doi:10.1038/ng.3656 (2016).

5 Genomes Project, C. *et al.* A global reference for human genetic variation. *Nature* **526**, 68-74, doi:10.1038/nature15393 (2015).

6 Loh, P. R., Kichaev, G., Gazal, S., Schoech, A. P. & Price, A. L. Mixed-model association for biobank-scale datasets. *Nat Genet* **50**, 906-908, doi:10.1038/s41588-018-0144-6 (2018).

7 Choi, S. W. & O'Reilly, P. F. PRSice-2: Polygenic Risk Score software for biobank-scale data. *GigaScience* **8**, giz082, doi:10.1093/gigascience/giz082 (2019).

8 Vilhjálmsson, B. J. *et al.* Modeling Linkage Disequilibrium Increases Accuracy of Polygenic Risk Scores. *American journal of human genetics* **97**, 576-592, doi:10.1016/j.ajhg.2015.09.001 (2015).

9 Dashti, H. S. *et al.* Genome-wide association study identifies genetic loci for self-reported habitual sleep duration supported by accelerometer-derived estimates. *Nat Commun* **10**, 1100, doi:10.1038/s41467-019-08917-4 (2019).

10 Malik, R. *et al.* Multiancestry genome-wide association study of 520,000 subjects identifies 32 loci associated with stroke and stroke subtypes. *Nat Genet* **50**, 524-537, doi:10.1038/s41588-018-0058-3 (2018).

11 Traylor, M. *et al.* Genetic risk factors for ischaemic stroke and its subtypes (the METASTROKE collaboration): a meta-analysis of genome-wide association studies. *Lancet Neurol* **11**, 951-962, doi:10.1016/s1474-4422(12)70234-x (2012).

12 Neurology Working Group of the Cohorts for Heart and Aging Research in Genomic Epidemiology (CHARGE) Consortium, t. S. G. N. S., and the International Stroke Genetics Consortium (ISGC);. Identification of additional risk loci for stroke and small vessel disease: a meta-analysis of genome-wide association studies. *Lancet Neurol* **15**, 695-707, doi:10.1016/s1474-4422(16)00102-2 (2016).

13 Psaty, B. M. *et al.* Cohorts for Heart and Aging Research in Genomic Epidemiology (CHARGE) Consortium: Design of prospective meta-analyses of genome-wide association studies from 5 cohorts. . *Circ Cardiovasc Genet* **2**, 273-280 (2009).

14 Harris, T. B. *et al.* Age, Gene/Environment Susceptibility-Reykjavik Study: multidisciplinary applied phenomics. *Am J Epidemiol* **165**, 1076-1087 (2007).

15 Fried, L. P. *et al.* The Cardiovascular Health Study: design and rationale. *Ann Epidemiol* **1**, 263-276, doi:10.1016/1047-2797(91)90005-w (1991).

16 Longstreth, W. T., Jr. *et al.* Frequency and predictors of stroke death in 5,888 participants in the Cardiovascular Health Study. *Neurology* **56**, 368-375 (2001).

17 Price, T. R., Psaty, B., O'Leary, D., Burke, G. & Gardin, J. Assessment of cerebrovascular disease in the Cardiovascular Health Study. *Ann Epidemiol* **3**, 504-507 (1993).

18 SHEP_Cooperative_Research_Group. Prevention of stroke by antihypertensive drug treatment in older persons with isolated systolic hypertension. Final results of the Systolic Hypertension in the Elderly Program (SHEP). SHEP Cooperative Research Group. *JAMA* **265**, 3255-3264 (1991).

19 Adams, H. P. *et al.* Classification of subtype of acute ischemic stroke. Definitions for use in a multicenter clinical trial. TOAST. Trial of Org 10172 in Acute Stroke Treatment. *Stroke* **24**, 35-41, doi:10.1161/01.str.24.1.35 (1993).

20 Dawber, T. R. & Kannel, W. B. The Framingham study. An epidemiological approach to coronary heart disease. *Circulation* **34**, 553-555 (1966).

21 Feinleib, M., Kannel, W. B., Garrison, R. J., McNamara, P. M. & Castelli, W. P. The Framingham Offspring Study. Design and preliminary data. *Prev Med* **4**, 518-525 (1975).

22 Splansky, G. L. *et al.* The Third Generation Cohort of the National Heart, Lung, and Blood Institute's Framingham Heart Study: design, recruitment, and initial examination. *Am J Epidemiol* **165**, 1328-1335 (2007).

23 Carandang, R. *et al.* Trends in incidence, lifetime risk, severity, and 30-day mortality of stroke over the past 50 years. *JAMA* **296**, 2939-2946 (2006).

24 Seshadri, S. *et al.* The lifetime risk of stroke: estimates from the Framingham Study. *Stroke* **37**, 345-350 (2006).

25 Wolf, P. A., Kannel, W. B. & Dawber, T. R. Prospective investigations: the Framingham study and the epidemiology of stroke. *Adv Neurol* **19**, 107-120 (1978).

26 Vartiainen, E. *et al.* Thirty-five-year trends in cardiovascular risk factors in Finland. *Int J Epidemiol* **39**, 504-518, doi:10.1093/ije/dyp330 (2010).

27 Tolonen, H. *et al.* The validation of the Finnish Hospital Discharge Register and Causes of Death Register data on stroke diagnoses. *Eur J Cardiovasc Prev Rehabil* **14**, 380-385, doi:10.1097/01.hjr.0000239466.26132.f2 (2007).

28 Hofman, A. Recent trends in cardiovascular epidemiology. *Eur J Epidemiol* **24**, 721-723 (2009).

29 Pahor, M. & Kritchevsky, S. Research hypotheses on muscle wasting, aging, loss of function and disability. *J Nutr Health Aging* **2**, 97-100 (1998).

30 Cesari, M. *et al.* Inflammatory markers and onset of cardiovascular events: results from the Health ABC study. *Circulation* **108**, 2317-2322, doi:10.1161/01.cir.0000097109.90783.fc (2003).

31 Houston, D. K. *et al.* Dietary fat and cholesterol and risk of cardiovascular disease in older adults: the Health ABC Study. *Nutr Metab Cardiovasc Dis* **21**, 430-437, doi:10.1016/j.numecd.2009.11.007 (2011).

32 Hofman, A. *et al.* The Rotterdam Study: objectives and design update. *Eur J Epidemiol* **22**, 819-829 (2007).

33 Hofman, A. *et al.* The Rotterdam Study: 2010 objectives and design update. *Eur J Epidemiol* **24**, 553-572 (2009).

34 Hofman, A., Grobbee, D. E., de Jong, P. T. & van den Ouweland, F. A. Determinants of disease and disability in the elderly: the Rotterdam Elderly Study. *Eur J Epidemiol* **7**, 403-422 (1991).

35 Bots, M. L. *et al.* Prevalence of stroke in the general population. The Rotterdam Study. *Stroke* **27**, 1499-1501 (1996).

36 Hollander, M. *et al.* Incidence, risk, and case fatality of first ever stroke in the elderly population. The Rotterdam Study. *J Neurol Neurosurg Psychiatry* **74**, 317-321 (2003).

37 Volzke, H. *et al.* Cohort profile: the study of health in Pomerania. *Int J Epidemiol* **40**, 294-307.

38 Szentkiralyi, A., Volzke, H., Hoffmann, W., Happe, S. & Berger, K. A time sequence analysis of the relationship between cardiovascular risk factors, vascular diseases and restless legs syndrome in the general population. *J Sleep Res* **22**, 434-442, doi:10.1111/jsr.12040 (2013).

39 Ridker, P. M. *et al.* Rationale, design, and methodology of the Women's Genome Health Study: a genome-wide association study of more than 25,000 initially healthy american women. *Clin Chem* **54**, 249-255, doi:10.1373/clinchem.2007.099366 (2008).

40 Bild, D. E. *et al.* Multi-ethnic study of atherosclerosis: objectives and design. *Am J Epidemiol* **156**, 871-881 (2002).

41 Kawasaki, R. *et al.* Retinal microvascular signs and risk of stroke: the Multi-Ethnic Study of Atherosclerosis (MESA). *Stroke* **43**, 3245-3251, doi:10.1161/strokeaha.112.673335 (2012).

42 Shepherd, J. *et al.* Pravastatin in elderly individuals at risk of vascular disease (PROSPER): a randomised controlled trial. *Lancet* **360**, 1623-1630 (2002).

43 Shepherd, J. *et al.* The design of a prospective study of Pravastatin in the Elderly at Risk (PROSPER). PROSPER Study Group. PROspective Study of Pravastatin in the Elderly at Risk. *Am J Cardiol* **84**, 1192-1197 (1999).

44 3C-Study-Group. Vascular factors and risk of dementia: design of the Three-City Study and baseline characteristics of the study population. *Neuroepidemiology* **22**, 316-325 (2003).

45 Riboli, E. *et al.* European Prospective Investigation into Cancer and Nutrition (EPIC): study populations and data collection. *Public Health Nutr* **5**, 1113-1124, doi:10.1079/PHN2002394 (2002).

46 Danesh, J. *et al.* EPIC-Heart: the cardiovascular component of a prospective study of nutritional, lifestyle and biological factors in 520,000 middle-aged participants from 10 European countries. *Eur J Epidemiol* **22**, 129-141, doi:10.1007/s10654-006-9096-8 (2007).

47 InterAct, C. *et al.* Design and cohort description of the InterAct Project: an examination of the interaction of genetic and lifestyle factors on the incidence of type 2 diabetes in the EPIC Study. *Diabetologia* **54**, 2272-2282, doi:10.1007/s00125-011-2182-9 (2011).

48 Debette, S. *et al.* Common variation in PHACTR1 is associated with susceptibility to cervical artery dissection. *Nat Genet* **47**, 78-83, doi:10.1038/ng.3154 (2015).

49 Ay, H. *et al.* A computerized algorithm for etiologic classification of ischemic stroke: the Causative Classification of Stroke System. *Stroke* **38**, 2979-2984 (2007).

50 Carty, C. L. *et al.* Meta-Analysis of Genome-Wide Association Studies Identifies Genetic Risk Factors for Stroke in African Americans. *Stroke* **46**, 2063-2068, doi:10.1161/strokeaha.115.009044 (2015).

51 Taylor, H. A., Jr. The Jackson Heart Study: an overview. *Ethn Dis* **15**, S6-1-3 (2005).

52 Carpenter, M. A. *et al.* Laboratory, reading center, and coordinating center data management methods in the Jackson Heart Study. *Am J Med Sci* **328**, 131-144 (2004).

53 Fuqua, S. R. *et al.* Recruiting African-American research participation in the Jackson Heart Study: methods, response rates, and sample description. *Ethn Dis* **15**, S6-18-29 (2005).

54 Payne, T. J. *et al.* Sociocultural methods in the Jackson Heart Study: conceptual and descriptive overview. *Ethn Dis* **15**, S6-38-48 (2005).

55 Wyatt, S. B. *et al.* Prevalence, awareness, treatment, and control of hypertension in the Jackson Heart Study. *Hypertension* **51**, 650-656, doi:10.1161/HYPERTENSIONAHA.107.100081 (2008).

56 Keku, E. *et al.* Cardiovascular disease event classification in the Jackson Heart Study: methods and procedures. *Ethn Dis* **15**, S6-62-70 (2005).

57 Rosamond, W. D. *et al.* Stroke incidence and survival among middle-aged adults - 9-year follow-up of the Atherosclerosis Risk in Communities (ARIC) cohort. *Stroke* **30**, 736-743, doi:10.1161/01.str.30.4.736 (1999).

58 Power, C. & Elliott, J. Cohort profile: 1958 British birth cohort (National Child Development Study). *Int J Epidemiol* **35**, 34-41, doi:10.1093/ije/dyi183 (2006).

59 Kubo, M. *et al.* A nonsynonymous SNP in PRKCH (protein kinase C eta) increases the risk of cerebral infarction. *Nat Genet* **39**, 212-217, doi:10.1038/ng1945 (2007).

60 Psaty, B. M. *et al.* The risk of myocardial infarction associated with the combined use of estrogens and progestins in postmenopausal women. *Arch Intern Med* **154**, 1333-1339 (1994).

61 Klungel, O. H. *et al.* Antihypertensive drug therapies and the risk of ischemic stroke. *Arch Intern Med* **161**, 37-43 (2001).

62 Psaty, B. M. *et al.* The risk of myocardial infarction associated with antihypertensive drug therapies. *JAMA* **274**, 620-625 (1995).

63 O'Donnell, M. *et al.* Rationale and design of INTERSTROKE: a global case-control study of risk factors for stroke. *Neuroepidemiology* **35**, 36-44, doi:10.1159/000306058 (2010).

64 Melander, O. *et al.* Novel and conventional biomarkers for prediction of incident cardiovascular events in the community. *JAMA* **302**, 49-57, doi:10.1001/jama.2009.943 (2009).

65 Saleheen, D. *et al.* The Pakistan Risk of Myocardial Infarction Study: a resource for the study of genetic, lifestyle and other determinants of myocardial infarction in South Asia. *Eur J Epidemiol* **24**, 329-338, doi:10.1007/s10654-009-9334-y (2009).

66 Jood, K., Ladenvall, C., Rosengren, A., Blomstrand, C. & Jern, C. Family history in ischemic stroke before 70 years of age: the Sahlgrenska Academy Study on Ischemic Stroke. *Stroke* **36**, 1383-1387, doi:10.1161/01.STR.0000169944.46025.09 (2005).

67 Traylor, M. *et al.* Genetic Architecture of Lacunar Stroke. *Stroke* **46**, 2407-2412, doi:10.1161/STROKEAHA.115.009485 (2015).

68 Berberian, K. M., van Duijn, C. M., Hoes, A. W., Valkenburg, H. A. & Hofman, A. Alcohol and mortality. Results from the EPOZ (Epidemiologic Study of Cardiovascular Risk Indicators) follow-up study. *European Journal of Epidemiology* **10**, 587-593, doi:10.1007/BF01719577 (1994).

69 Mars, N. *et al.* Polygenic and clinical risk scores and their impact on age at onset and prediction of cardiometabolic diseases and common cancers. *Nat Med* **26**, 549-557, doi:10.1038/s41591-020-0800-0 (2020).

70 Tolonen, H. *et al.* The validation of the Finnish Hospital Discharge Register and Causes of Death Register data on stroke diagnoses. *Eur J Cardiovasc Prev Rehabil* **14**, 380-385, doi:10.1097/01.hjr.0000239466.26132.f2 (2007).

71 Krokstad, S. *et al.* Cohort Profile: the HUNT Study, Norway. *International Journal of Epidemiology* **42**, 968-977, doi:10.1093/ije/dys095 (2013).

72 Varmdal, T. *et al.* Comparison of the validity of stroke diagnoses in a medical quality register and an administrative health register. *Scandinavian Journal of Public Health* **44**, 143-149, doi:10.1177/1403494815621641 (2016).

73 Varmdal, T. *et al.* Inter-rater reliability of a national acute stroke register. *BMC research notes* **8**, 584, doi:10.1186/s13104-015-1556-3 (2015).

74 Leitsalu, L. *et al.* Cohort Profile: Estonian Biobank of the Estonian Genome Center, University of Tartu. *International Journal of Epidemiology* **44**, 1137-1147, doi:10.1093/ije/dyt268 (2015).

75 Vibo, R. *et al.* Estonian young stroke registry: High burden of risk factors and high prevalence of cardiomebolic and large-artery stroke. *Eur Stroke J* **6**, 262-267, doi:10.1177/23969873211040990 (2021).

76 Carey, D. J. *et al.* The Geisinger MyCode community health initiative: an electronic health record–linked biobank for precision medicine research. *Genetics in Medicine* **18**, 906-913, doi:10.1038/gim.2015.187 (2016).

77 Chaudhary, D. *et al.* Trends in ischemic stroke outcomes in a rural population in the United States. *Journal of the Neurological Sciences* **422**, 117339, doi:10.1016/j.jns.2021.117339 (2021).

78 Li, J. *et al.* Polygenic Risk Scores Augment Stroke Subtyping. *Neurology Genetics* **7**, e560, doi:10.1212/NXG.0000000000000560 (2021).

79 Frikke-Schmidt, R. *et al.* Association of loss-of-function mutations in the ABCA1 gene with high-density lipoprotein cholesterol levels and risk of ischemic heart disease. *JAMA* **299**, 2524-2532, doi:10.1001/jama.299.21.2524 (2008).

80 Jørgensen, A. B., Frikke-Schmidt, R., Nordestgaard, B. G. & Tybjærg-Hansen, A. Loss-of-function mutations in APOC3 and risk of ischemic vascular disease. *The New England Journal of Medicine* **371**, 32-41, doi:10.1056/NEJMoa1308027 (2014).

81 Pedersen, D. A. *et al.* The Danish Twin Registry: An Updated Overview. *Twin Research and Human Genetics: The Official Journal of the International Society for Twin Studies* **22**, 499-507, doi:10.1017/thg.2019.72 (2019).

82 Hess, D. T. The Danish National Patient Register. *Surgery for Obesity and Related Diseases: Official Journal of the American Society for Bariatric Surgery* **12**, 304, doi:10.1016/j.soard.2015.11.001 (2016).

83 Lynge, E., Sandegaard, J. L. & Rebolj, M. The Danish National Patient Register. *Scand J Public Health* **39**, 30-33, doi:10.1177/1403494811401482 (2011).

84 Group, A. I. Study design of ASPirin in Reducing Events in the Elderly (ASPREE): a randomized, controlled trial. *Contemporary Clinical Trials* **36**, 555-564, doi:10.1016/j.cct.2013.09.014 (2013).

85 McNeil, J. J. *et al.* Effect of Aspirin on Cardiovascular Events and Bleeding in the Healthy Elderly. *The New England Journal of Medicine* **379**, 1509-1518, doi:10.1056/NEJMoa1805819 (2018).

86 McNeil, J. J. *et al.* Effect of Aspirin on Disability-free Survival in the Healthy Elderly. *The New England Journal of Medicine* **379**, 1499-1508, doi:10.1056/NEJMoa1800722 (2018).

87 Neumann, J. T. *et al.* Predictive Performance of a Polygenic Risk Score for Incident Ischemic Stroke in a Healthy Older Population. *Stroke* **52**, 2882-2891, doi:10.1161/STROKEAHA.120.033670 (2021).

88 Stroke--1989. Recommendations on stroke prevention, diagnosis, and therapy. Report of the WHO Task Force on Stroke and other Cerebrovascular Disorders. *Stroke* **20**, 1407-1431, doi:10.1161/01.str.20.10.1407 (1989).

89 Liman, T. G. *et al.* Prediction of vascular risk after stroke - protocol and pilot data of the Prospective Cohort with Incident Stroke (PROSCIS). *Int J Stroke* **8**, 484-490, doi:10.1111/j.1747-4949.2012.00871.x (2013).

90 Heuschmann, P. U. *et al.* Prevalence and determinants of systolic and diastolic cardiac dysfunction and heart failure in acute ischemic stroke patients: The SICFAIL study. *ESC Heart Fail* **8**, 1117-1129, doi:10.1002/ehf2.13145 (2021).

91 Nederkoorn, P. J. *et al.* The Dutch String-of-Pearls Stroke Study: protocol of a large prospective multicenter genetic cohort study. *Int J Stroke* **10**, 120-122, doi:10.1111/ijs.12359 (2015).

92 Manniën, J. *et al.* The parelsnoer institute: A national network of standardized clinical biobanks in the Netherlands. *Open Journal of Bioresources* **4**, 3 (2017).

93 Huisman, M. H. *et al.* Population based epidemiology of amyotrophic lateral sclerosis using capture-recapture methodology. *J Neurol Neurosurg Psychiatry* **82**, 1165-1170, doi:10.1136/jnnp.2011.244939 (2011).

94 van Rheenen, W. *et al.* Common and rare variant association analyses in amyotrophic lateral sclerosis identify 15 risk loci with distinct genetic architectures and neuron-specific biology. *Nat Genet* **53**, 1636-1648, doi:10.1038/s41588-021-00973-1 (2021).

95 Chen, Z. *et al.* Cohort profile: the Kadoorie Study of Chronic Disease in China (KSCDC). *International Journal of Epidemiology* **34**, 1243-1249, doi:10.1093/ije/dyi174 (2005).

96 Sudlow, C. L. & Warlow, C. P. Comparable studies of the incidence of stroke and its pathological types: results from an international collaboration. International Stroke Incidence Collaboration. *Stroke* **28**, 491-499, doi:10.1161/01.str.28.3.491 (1997).

97 Ibanez, L. *et al.* Multi-ancestry genetic study in 5,876 patients identifies an association between excitotoxic genes and early outcomes after acute ischemic stroke. *medRxiv*, doi:10.1101/2020.10.29.20222257 (2020).

98 Mola-Caminal, M. *et al.* PATJ Low Frequency Variants Are Associated With Worse Ischemic Stroke Functional Outcome. *Circ Res* **124**, 114-120, doi:10.1161/circresaha.118.313533 (2019).

99 Domingues-Montanari, S. *et al.* KCNK17 genetic variants in ischemic stroke. *Atherosclerosis* **208**, 203-209, doi:10.1016/j.atherosclerosis.2009.07.023 (2010).

100 Obón-Santacana, M. *et al.* GCAT|Genomes for life: a prospective cohort study of the genomes of Catalonia. *BMJ Open* **8**, e018324, doi:10.1136/bmjopen-2017-018324 (2018).

101 Galván-Femenía, I. *et al.* Multitrait genome association analysis identifies new susceptibility genes for human anthropometric variation in the GCAT cohort. *J Med Genet* **55**, 765-778, doi:10.1136/jmedgenet-2018-105437 (2018).

102 Fernández-Cadenas, I. *et al.* GRECOS Project (Genotyping Recurrence Risk of Stroke): The Use of Genetics to Predict the Vascular Recurrence After Stroke. *Stroke* **48**, 1147-1153, doi:10.1161/strokeaha.116.014322 (2017).

103 Riba, I. *et al.* Cognitive assessment protocol design in the ISSYS (Investigating Silent Strokes in hYpertensives: a magnetic resonance imaging Study). *J Neurol Sci* **322**, 79-81, doi:10.1016/j.jns.2012.06.015 (2012).

104 Kumar, A. *et al.* Association of SUMOylation Pathway Genes With Stroke in a Genome-Wide Association Study in India. *Neurology* **97**, e345-e356, doi:10.1212/WNL.0000000000012258 (2021).

105 Jones, W. J., Williams, L. S. & Meschia, J. F. Validating the Questionnaire for Verifying Stroke-Free Status (QVSFS) by neurological history and examination. *Stroke* **32**, 2232-2236, doi:10.1161/hs1001.096191 (2001).

106 Kuriyama, S. *et al.* Cohort Profile: Tohoku Medical Megabank Project Birth and Three-Generation Cohort Study (TMM BirThree Cohort Study): rationale, progress and perspective. *Int J Epidemiol* **49**, 18-19m, doi:10.1093/ije/dyz169 (2020).

107 Hozawa, A. *et al.* Study Profile of the Tohoku Medical Megabank Community-Based Cohort Study. *J Epidemiol* **31**, 65-76, doi:10.2188/jea.JE20190271 (2021).

108 Gaziano, J. M. *et al.* Million Veteran Program: A mega-biobank to study genetic influences on health and disease. *Journal of clinical epidemiology* **70**, 214-223, doi:10.1016/j.jclinepi.2015.09.016 (2016).

109 Hunter-Zinck, H. *et al.* Genotyping Array Design and Data Quality Control in the Million Veteran Program. *American journal of human genetics* **106**, 535-548, doi:10.1016/j.ajhg.2020.03.004 (2020).

110 Fang, H. *et al.* Harmonizing Genetic Ancestry and Self-identified Race/Ethnicity in Genome-wide Association Studies. *American journal of human genetics* **105**, 763-772, doi:10.1016/j.ajhg.2019.08.012 (2019).

111 Akpalu, A. *et al.* Phenotyping Stroke in Sub-Saharan Africa: Stroke Investigative Research and Education Network (SIREN) Phenomics Protocol. *Neuroepidemiology* **45**, 73-82, doi:10.1159/000437372 (2015).

112 Owolabi, M. O. *et al.* Dominant modifiable risk factors for stroke in Ghana and Nigeria (SIREN): a case-control study. *Lancet Glob Health* **6**, e436-e446, doi:10.1016/S2214-109X(18)30002-0 (2018).

113 Salomaa, V. Genetic and Environmental Contributions to Cardiovascular Risk: Lessons From North Karelia and FINRISK. *Glob Heart* **11**, 229-233, doi:10.1016/j.gheart.2016.04.008 (2016).

114 Pärn, K. *et al.* in *protocols.io* (2019).

115 Roden, D. M. *et al.* Development of a large-scale de-identified DNA biobank to enable personalized medicine. *Clin Pharmacol Ther* **84**, 362-369, doi:10.1038/clpt.2008.89 (2008).

116 Kim, B. J. *et al.* Current status of acute stroke management in Korea: a report on a multicenter, comprehensive acute stroke registry. *Int J Stroke* **9**, 514-518, doi:10.1111/ijs.12199 (2014).

117 Kim, B. J. *et al.* Case characteristics, hyperacute treatment, and outcome information from the clinical research center for stroke-fifth division registry in South Korea. *J Stroke* **17**, 38-53, doi:10.5853/jos.2015.17.1.38 (2015).

118 Moon, S. *et al.* The Korea Biobank Array: Design and Identification of Coding Variants Associated with Blood Biochemical Traits. *Sci Rep* **9**, 1382, doi:10.1038/s41598-018-37832-9 (2019).

119 Kim, Y., Han, B. G. & Ko, G. E. S. g. Cohort Profile: The Korean Genome and Epidemiology Study (KoGES) Consortium. *Int J Epidemiol* **46**, 1350, doi:10.1093/ije/dyx105 (2017).
